# Conserved Accession–Lot Geometry but Limited Individual Spectral Retrieval: Bidirectional Transfer Across Two Hyperspectral Imaging Systems

**DOI:** 10.3390/s26216931

**Published:** 2026-11-02

**Authors:** Renan Falcioni, Roney Berti de Oliveira, Marcelo Luiz Chicati, José A. M. Demattê, Marcos Rafael Nanni

**Affiliations:** 1 Graduate Program in Agronomy, State University of Maringá, Av. Colombo 5790, Maringá 87020-900, PR, Brazil; rboliveira@uem.br (R.B.d.O.); mlchicati@uem.br (M.L.C.); mrnanni@uem.br (M.R.N.); 2 Department of Soil Science, Luiz de Queiroz College of Agriculture, University of São Paulo, Av. Pádua Dias, 11, Piracicaba 13418-260, SP, Brazil; jamdemat@usp.br

**Keywords:** calibration transfer, grain legumes, CORAL, seed phenotyping, spectral geometry

## Abstract

**Highlights:**

**What are the main findings?**

- Accession–lot spectral geometry was conserved after within-sensor wavelength standardisation, with distance correlations of 0.958–0.996.
- PDS and CORAL improved performance in all 12 specified fixed-split PCA–LDA comparisons with direct transfer; full-grid ridge DS achieved higher mean macro-F1 than both in all six representation–direction combinations.

**What is the implication of the main finding?**

- High within-sensor accuracy does not establish cross-system prediction; numerical wavelength alignment, adaptation, and pair-preserving validation address distinct requirements.
- The same 3200 physical seeds retained their positions and orientation during same-day imaging, providing paired spectra for cross-system comparison.

**Abstract:**

Cross-system portability of hyperspectral seed classifiers was evaluated using paired Headwall and AisaFENIX spectra from 3200 seeds representing 32 grain-legume accession–lots. The same seeds retained their positions and orientation during same-day acquisitions. Spectra were smoothed on their native axes and interpolated over 450–950 nm at 5 nm intervals. Accession-distance correlations after within-sensor wavelength standardisation were 0.958–0.996. In a separate within-accession diagnostic, correlation-based individual retrieval accuracy was 0.81–1.44% against a 1% random benchmark. Across 30 overlapping pair-preserving splits, direct-transfer principal component analysis–linear discriminant analysis (PCA–LDA) macro-F1 ranged from 0.367 to 0.646. Piecewise direct standardisation (PDS) and correlation alignment (CORAL) improved performance in all 12 fixed-split comparisons with direct transfer, with paired-bootstrap intervals excluding zero. Full-grid ridge direct standardisation (DS) achieved higher mean macro-F1 than both in all six representation–direction combinations. Within-accession calibration-pair derangement had small, setting-dependent effects, whereas accession mismatching strongly reduced PDS performance. Low-budget CORAL benefited from accession-balanced target sampling. These results demonstrate conserved relative accession–lot structure and calibration-dependent PCA–LDA transfer within the studied population. Validation across new lots, acquisition days and instrument units would extend their generality.

## 1. Introduction

Hyperspectral imaging combines spectroscopy with spatially resolved sensing, enabling non-destructive characterisation of individual seeds across contiguous wavelength channels. Visible–near-infrared (VNIR) and visible–near-infrared–shortwave-infrared (VIS–NIR–SWIR) measurements provide spectral information for seed accession classification [1,2]. Cross-system classification requires evaluating whether a decision rule trained in one acquisition domain remains valid in the other.

The seed-imaging literature shows both the analytical potential of hyperspectral measurements and the diversity of assessment pipelines. End-to-end frameworks such as HyperSeed formalise segmentation, spectral extraction, and seed-level analysis [3], while controlled perturbation studies show that assignment errors, spectral alteration, and training-set size can materially affect reported classification performance [4]. Variety discrimination has also been demonstrated in soybean and rice [5,6]. These results are crop and task specific and remain conditional on the sensor, sample set, preprocessing, and validation design.

Hyperspectral seed analysis also addresses physiological endpoints such as germination [7]. Recent studies have used rice-seed data with credibility-focused evaluation [8] and maize-seed data for open-set incremental recognition [9]. The methodological question is therefore no longer only whether spectra can classify seeds but whether models remain credible with new samples and open classes, under altered acquisition conditions and operationally relevant shifts.

Reproducibility and measurement control are central to this transition. Reviews of plant phenotyping and precision-agriculture hyperspectral imaging identify standardisation, robustness, and external validation as unresolved requirements [10,11], and instrument quality assurance can affect neural-network-supported phenotyping [12]. An optical spectrum is therefore jointly determined by the biological object and the measurement process.

Optical measurement context matters. Canopy observations from PRISMA and Sentinel-2 have been used to predict grain nutrient composition [13], while close-range hyperspectral spectroscopy has tracked leaf-water dynamics [14]. These studies provide optical measurement context at canopy and leaf scales. In seed imaging, detector response, illumination geometry, spectral bandwidth, wavelength calibration, radiometric processing, sample presentation, and region-of-interest (ROI) definition can all contribute to domain shift. High within-sensor accuracy is therefore a sensor-conditional benchmark, not evidence of instrument-independent accession classification.

Calibration transfer and domain adaptation provide complementary responses. Cross-facility plant-phenotyping studies have progressed from explicit spectral-space adjustment to shared latent spaces and adversarial representations [15,16,17]. Changing the imaging system can alter the domain in which a prediction model operates, motivating explicit treatment of cross-system differences.

Seed and grain calibration transfer has established precedents. NIR instrument networks have been standardised for rapeseed constituent analysis [18], and single-seed soybean trait calibrations have been transferred between sister NIR analysers [19]. Single-kernel rice work has addressed transfer between intact, dehusked and milled forms [20], while wheat and maize kernel studies have compared wavelength selection for inter-instrument discriminant transfer [21]. Hyperspectral maize- and wheat-seed studies also address cross-year recognition [22,23]. These precedents distinguish instrument, harvest-year and sample-form shifts, motivating separate assessment of accession geometry, individual-seed retrieval and classifier transfer.

The term *harmonisation* requires care. Satellite studies distinguish empirical spectral correspondence and cross-calibration from explicit treatment of spectral-response effects [24], and recent work estimates bandpass adjustments rather than relying on nominal wavelength labels alone [25]. Although satellite and close-range systems differ, the principle is transferable: interpolation to matching band centres does not equalise spectral-response functions, optical bandwidths, or spatial sampling.

We therefore used piecewise direct standardisation (PDS) and correlation alignment (CORAL) as two interpretable transfer baselines. Classical multivariate instrument standardisation introduced piecewise mappings between instruments [26], whereas CORAL aligns source and target second-order statistics without target labels in its objective [27]. PDS and CORAL represent distinct, explicit transfer mechanisms that can be compared within the same pair-preserving design.

Preprocessing and wavelength selection also require endpoint-specific justification. Competitive shrinkage and hybrid searches illustrate available strategies [28,29], but feature selection is valid only when embedded in a leakage-safe, classification-appropriate validation scheme. Validation must reflect the physical data hierarchy because leakage across training and test partitions can produce irreproducible claims [30]. Under domain shift, conformal inference beyond exchangeability distinguishes nominal coverage from observed target-domain behaviour [31]. Resampling, preprocessing, model selection, and final evaluation must therefore preserve the observational hierarchy and remain separated.

The paired design also permits structural analyses beyond accuracy. Generalised Procrustes analysis compares configurations after removing non-substantive transformations [32], whereas Mantel statistics are complementary matrix-association measures rather than causal tests [33].

The present study evaluates conservation of accession–lot spectral geometry and bidirectional transfer of accession classifiers based on principal component analysis, followed by linear discriminant analysis (PCA–LDA), with within-accession retrieval of individual seed spectra as a complementary diagnostic. Its contribution is the comparison of paired Headwall and AisaFENIX seed spectra under a common numerical wavelength support and pair-preserving partitions, including calibration-composition and pairing controls. The same 3200 physical seeds retained their positions and orientation during same-day acquisitions with both cameras.

Four questions structure the analysis: (Q1) How stable is accession–lot geometry across systems? (Q2) How does direct-transfer PCA–LDA macro-F1 compare with the corresponding source-sensor reference? (Q3) How much do the specified adaptation pipelines improve direct transfer? (Q4) What does individual seed pairing add to accession-matched calibration for PDS? Q1 is assessed through per-representation geometry statistics, seed-stratified bootstrap intervals and split-half benchmarks, with individual spectral retrieval as a supplementary diagnostic of within-accession similarity. Q2 uses source-reference-minus-direct differences on matched seed partitions. The formal inferential component of Q3 comprises 12 fixed-split PDS/CORAL-versus-direct contrasts with paired-bootstrap intervals and Holm adjustment; an interval wholly above zero supports a positive conditional gain. Q4 uses repeated within-accession derangements and a separate cross-accession negative control. Cross-direction rankings and supplementary calibration, spectral-support, panel and uncertainty analyses are descriptive.

## 2. Materials and Methods

### 2.1. Seed Material and Paired Study Design

The dataset comprised 3200 distinct dry seeds from 32 accessions, with 100 physical seeds per accession. Accessions A01–A26 and A29–A32 were *Phaseolus vulgaris* L.; A27 was *Vigna angularis* (Willd.) Ohwi and H. Ohashi; and A28 was *Cajanus cajan* (L.) Huth. The materials originated from a maintained germplasm bank with curated accession identities and documented provenance [1,2]. Each accession was represented by one experimental seed lot. Seeds were stored in a low-humidity chamber and had a moisture content of approximately 13% before imaging. Seeds were held in fixed Petri-dish supports, preserving their positions, faces and orientations, while the camera was exchanged on the same day. Groups of 100 distinct seeds were passed once under each camera, with the support arrangement and conveyor speed unchanged. These paired seed-level spectra were used to evaluate cross-system transfer.

The workflow for paired acquisition, numerical common-grid processing and leakage-safe evaluation is summarised in Figure 1. Supporting details and results are provided in Figures S1–S10 and Tables S1–S26.

### 2.2. Hyperspectral Acquisition and Extraction of Seed-Level Spectra

#### 2.2.1. Headwall VNIR Measurements

Headwall measurements were acquired with a Headwall Photonics A-Series push-broom system (Headwall Photonics, Inc., Bolton, MA, USA). The calibrated wavelength axis comprised 825 coordinates from 423.34 to 1022.07 nm, with a median numerical interval of approximately 0.73 nm. Four sessions were used for equipment calibration and illumination adjustments. For acquisition of the analysed spectra, each pass carried a group of 100 distinct seeds in fixed Petri-dish supports, arranged in a single layer on a low-reflectance background. The sensor-to-sample distance was 85 cm, and illumination was provided by twelve 20 W halogen lamps. Laboratory temperature was approximately 25 °C. Camera cooling and thermal stabilisation preceded acquisition. Dark-current frames and a 99% Spectralon reference (Labsphere, Inc., North Sutton, NH, USA) supported reflectance conversion. Acquisition used a 39 ms integration time, a 40 ms frame period and a platform translation speed of 50 mm/min [1]. Each group was scanned once with each camera on the same day, with seed position and orientation preserved during camera exchange.

Calibrated cubes were processed in ENVI Classic 5.4 (Harris Geospatial Solutions, Inc., Broomfield, CO, USA). One 10 × 10-pixel ROI was defined in the central interior seed-coat region of each seed, avoiding boundaries, background and evident specular artefacts. Reflectance was averaged wavelength-wise across the 100 ROI pixels to obtain one spectrum per physical seed [1].

#### 2.2.2. AisaFENIX VIS–NIR–SWIR Measurements

AisaFENIX measurements were acquired with a Specim AisaFENIX push-broom system (Specim Spectral Imaging Ltd., Oulu, Finland). Three sessions were used for equipment calibration and illumination adjustments. Spectral acquisition used a line rate of 12 frames/s. Each group of 100 distinct seeds underwent one passage under the camera, with the fixed Petri-dish arrangement, seed orientation and conveyor speed maintained during camera exchange. The sensor-to-sample distance was 85 cm. Illumination was provided by twelve 20 W halogen lamps. Measurements were acquired under controlled laboratory temperature, with dark and Spectralon white references. Internal cooling was activated, and the camera was stabilised for 30 min. Radiometric calibration used CaliGeoPRO 2.2 (Specim Spectral Imaging Ltd.), including sensor-specific calibration and lookup files, followed by reflectance conversion in ENVI Classic 5.4 [2].

A central 10 × 10-pixel sampling window was manually placed within the central interior seed-coat region wherever feasible. Ten representative interior pixels were selected after excluding evident highlights, shadows, contaminants and marginal pixels; their reflectance was then averaged wavelength-wise to obtain one spectrum per physical seed [2]. Corresponding central interior regions of the same fixed seeds were sampled with both cameras, with pixel selection adapted to each system’s imaging geometry and spatial resolution.

The paired spectra were compared over a common numerical wavelength range (Section 2.3). The ROI averaging above concerns spatial sampling of the seed surface; native channel counts and common-grid interpolation concern spectral sampling.

### 2.3. Spectral Measurements and Analytical Range

The AisaFENIX spectra comprised 625 wavelength coordinates spanning 377.27–2502.75 nm (median numerical interval, 1.72 nm). The Headwall spectra comprised 825 coordinates spanning 423.34–1022.07 nm (median interval, 0.73 nm). Each system contributed 3200 spectra from 32 accessions, with 100 spectra per accession. The native channels differ in both number and wavelength coverage; the primary cross-system analysis used the same 101 coordinates from 450 to 950 nm for both cameras (Table 1).

Within the primary 450–950 nm interval, all analysed reflectance values were finite and within 0–1. Values above 1 occurred only outside this interval: eight AisaFENIX values below 381 nm and 990 Headwall values from 950.13 to 1022.07 nm. Within each accession, the fixed seed position linked the corresponding ROI and its averaged spectrum in each camera image, defining the 3200 cross-sensor pairs (Table S1).

### 2.4. Native-Axis Smoothing and Numerical Common-Grid Alignment

Savitzky–Golay filtering [34] was applied before resampling so that smoothing respected each native numerical wavelength axis. A third-order polynomial and an approximately 15 nm target span were used with reflected edge padding. This produced a 9-point AisaFENIX window (window length × median spacing, approximately 15.48 nm) and a 21-point Headwall window (approximately 15.33 nm). Smoothed spectra were linearly interpolated to 101 wavelength coordinates from 450 to 950 nm at 5 nm spacing. The interval was a conservative common support below the Headwall region containing reflectance values above one; those excursions are a range diagnostic, not an empirical signal-to-noise ratio (SNR) measurement. Neither the smoothing-window width nor the 5 nm coordinate spacing measures optical full width at half maximum (FWHM) or equalises spectral-response functions. A sensitivity analysis varied the nominal smoothing target to 5 and 25 nm while retaining the same preprocessing order (Supplementary Method S1.16; Table S23).

Three representations were evaluated: smoothed reflectance; standard normal variate (SNV), calculated independently for each spectrum [35]; and the first derivative of the common-grid reflectance, calculated with a third-order, 7-point Savitzky–Golay filter and a 5 nm wavelength increment. All matrices contained 3200 rows and 101 finite features.

Formal definitions and implementation details are provided in Supplementary Method S1.1.

### 2.5. Accession–Lot Geometry and Individual Spectral Retrieval

Accession centroids were calculated separately for each sensor and representation. Wavelength features were centred and standardised within each sensor before Euclidean distances were calculated among the 32 centroids. This removes sensor-specific per-band location and scale, so the geometry measures relative accession organisation. Cross-sensor distance agreement was quantified with a Spearman Mantel statistic, interpreted as matrix association rather than validation of absolute spectra or a causal test [33], and symmetric Procrustes analysis [32], each with 999 permutations. Per-representation results are primary. A consensus distance matrix was formed within each sensor by rank-normalising and averaging distance vectors from reflectance, SNV and derivatives; it summarises the same measurements and supplies no independent replication.

Each accession contained 100 seeds, and the 32 accession centroids yielded 496 pairwise distances. Tied ranks were averaged. Before symmetric Procrustes analysis, each distance matrix was converted to a 10-dimensional classical multidimensional-scaling configuration with cmdscale (add = TRUE). Formal definitions are provided in Supplementary Method S1.2.

As an accession-geometry stability reference, 50 seeds per accession were assigned to each of two disjoint halves using the same division for both systems. Thirty divisions were used to compare within-sensor accession-distance matrices with matched-half cross-sensor matrices; this assesses centroid stability across different seeds, not same-seed repeatability. We also excluded the first principal component (PC1) from a common basis fitted to the 64 sensor-specific accession centroids and repeated the geometry analysis after excluding A27 and A28. A paired, accession-stratified bootstrap resampled 100 seed pairs with replacement within each accession in 999 replicates, giving conditional 95% percentile intervals. Further comparisons used 450–700 and 700–950 nm, with 700 nm included in both ranges. These checks quantify stability within the observed accession–lots and acquisition setting (Supplementary Methods S1.8, S1.11 and S1.12; Tables S11, S16 and S17).

Individual spectral retrieval was examined as a within-accession diagnostic using the paired seed spectra. Each seed’s spectrum from one camera was compared with the 100 spectra from its accession in the other camera, giving a 1% top-1 expectation for random selection. Similarities were calculated after row-wise standardisation for complete profiles and for profiles residualised by subtracting the accession-specific mean within each sensor. For each representation and direction, we recorded how often the spectrum from the same seed was the most similar target spectrum. Mean paired similarity was compared with the values obtained under 999 within-accession permutations. This correlation-based search preceded calibration transfer and was separate from the accession-classification experiments.

Pearson correlation was equivalent to cosine similarity after row standardisation. Ties were resolved by selecting the first index; full permutation details are provided in Supplementary Method S1.2.

As complementary diagnostics, cross-sensor correlations were calculated within each accession separately for seed mean reflectance and sensor-specific PC1 scores, without row-wise normalisation and using 9999 within-accession permutations. Offsets from −10 to +10 and reversed order were examined as sensitivity controls, with joint adjustment across 44 comparisons (Supplementary Method S1.17; Table S26).

### 2.6. Cross-Instrument Classification and Adaptation

The primary transfer benchmark modelled accession identity as a 32-class nominal outcome using a fixed PCA–LDA classifier family to compare transfer pipelines. Within each source training set, features were centred and standardised, principal component analysis was fitted, and the smallest number of components explaining 99.5% of the variance was retained, subject to a minimum of two and a maximum of 60 or the applicable rank limit. Linear discriminant analysis was fitted to the retained scores. All preprocessing parameters were estimated from the corresponding development data [30,36].

Formal definitions of PCA–LDA, classwise metrics, and distribution-shift summaries are provided in Supplementary Method S1.3; the corresponding results are reported in Table S3. With *n* training seeds, *p* features, and *C* = 32 classes, the maximum number of components was min (60, *n* − *C*, *p*); at least two components were retained. Balanced development partitions gave equal class priors. Non-finite or near-zero feature standard deviations (<10^−10^) were replaced by 1 before standardisation.

As a within-sensor comparator, pooled-covariance shrinkage LDA used all 101 coordinates, training-only centring and scaling, equal class priors, and a covariance matrix of (1 − *λ*) **Σ** + *λ*·tr (**Σ**) **I**/*p*. The fixed reference was *λ* = 0.1, and 0.01 and 0.5 were sensitivity settings, evaluated on the same 30 held-out partitions without tuning against test labels. This single-parameter comparator differs from the two-parameter regularised discriminant analysis (RDA) procedure [37] (Supplementary Method S1.13; Table S19). The transfer comparisons used PCA–LDA throughout; shrinkage LDA provided an additional within-sensor reference.

Predictive performance was evaluated on held-out physical seeds across 30 pair-preserving stratified splits. In each split, 70 seeds per accession (*n* = 2240) formed the development/calibration partition, and 30 per accession (*n* = 960) formed the test partition. The identical seed indices were used for both instruments, all representations, both directions, and all transfer scenarios. Neither spectrum of a paired test seed was used for fitting or adaptation. This provides seed-level out-of-sample validation within the studied accession–lots. Source development and target calibration originated from the same seed population.

For each direction, five conditions were evaluated: source-sensor and target-sensor conditional references, direct transfer, PDS, and CORAL. The within-sensor references used the same seed-index partitions. Direct transfer applied the source-trained classifier to target spectra. PDS [26] fitted local regressions between physical seed pairs from target to source coordinates (Equations (1)–(3)) and reused the source-trained PCA–LDA; its half-window was two 5 nm grid points, and its ridge penalty was 10^−6^. CORAL [27] scaled the source-development and accession-balanced target-calibration data using pooled statistics, aligned source covariance with target covariance using a ridge penalty of 10^−4^, and refitted PCA–LDA (Equations (4) and (5)). These pipelines differ in mapping direction, supervision and classifier refitting, so their numerical ranking does not isolate the effect of the alignment operator.

(1) $$N_{j}=\left\{\max(1,j-h),\ldots ,\min(p,j+h)\right\},Z_{j}=\left[1,X_{T,N_{j}}\right]$$

(2) $${\hat{\beta }}_{j}={\left({Z_{j}}^{T}Z_{j}+\gamma P\right)}^{-1}{Z_{j}}^{T}x_{S,j}$$

(3) $${\hat{x}}_{T\to S,\mathrm{ij}}=\left[1,x_{T,i,N_{j}}\right]{\hat{\beta }}_{j},P=\mathrm{diag}(0,1,\ldots ,1),h=2,\gamma =10^{-6}$$

**Z***_d_* = (**X***_d_* − **μ**_P_)⊘**σ**_P_,**C***_d_* = cov(**Z***_d_*) + *ε***I** (4)

(5) $$A={C_{S}}^{-\frac{1}{2}}{C_{T}}^{\frac{1}{2}},{Z_{S}}^{*}=\left(Z_{S}-\mu_{S}\right)A+\mu_{T},\varepsilon =10^{-4}$$

In Equations (1)–(3), *j* indexes the output wavelength, *p* is the number of spectral coordinates, *N_j_* is the local target-column neighbourhood, **Z***_j_* contains the paired target calibration values and an intercept, and the response is source-band *j*. **P** leaves the intercept unpenalised; *h* = 2 and windows are truncated at spectral boundaries. In Equations (4) and (5), pooled means and standard deviations are estimated from source-development and target-calibration spectra. The covariance matrices and domain means refer to these scaled data; the identity matrix supplies the covariance ridge, and the inverse square root whitens the source before target recolouring. Non-finite or near-zero pooled standard deviations (<10^−10^) are replaced by one.

Macro-F1 was the primary metric because each accession had equal importance; accuracy and balanced accuracy were secondary metrics. Means and sample standard deviations across the 30 overlapping splits were descriptive.

Classes receiving no predictions had precision and F1 set to zero. Mean-vector and normalised covariance gaps were calculated in a scenario-specific pooled z-space, with the covariance denominator bounded below by 10^−12^ (Supplementary Method S1.3). These development/calibration diagnostics summarise the fitted alignment; predictive generalisation was evaluated separately on held-out seeds. We additionally fitted a regularised linear domain classifier using development data and evaluated its area under the receiver operating characteristic curve (AUC) on held-out paired seeds in the appropriate raw, PDS or CORAL feature space (Supplementary Method S1.14; Table S22). Its ability to detect shift is limited to the fitted linear decision function.

### 2.7. Pairing Control and Calibration-Budget Analysis

To assess the incremental value of exact physical pairs for PDS, target-calibration spectra were deranged within accession. Each source seed was paired with a different target seed from the same accession, retaining zero exact pairs while preserving accession composition. The fixed-split comparison used one derangement; a sensitivity analysis used five fixed-point-free derangements within each of 30 overlapping pair-preserving splits. A separate negative control mismatched every source accession to a different target accession, retaining the complete calibration sample and using five draws per split. This second control deliberately destroys accession correspondence as well as individual pairing and is not an alternative estimate of the value of exact within-accession identity.

For both smoothed reflectance and SNV, nested target-calibration budgets of 1, 2, 5, 10, 20, 40, and 70 spectra per accession were evaluated within the 30 paired splits. PDS used exact physical pairs; CORAL used the same target spectra in a label-free covariance objective, but their selection into accession-balanced panels required known accession labels. At one target spectrum per accession, the centred target covariance has rank at most 31 on the 101-feature grid. Target-only and pooled source-plus-labelled-target PCA–LDA comparators used matching target-labelled subsets. Target-only PCA–LDA with one target seed per class was not estimable because 32 observations across 32 classes leave zero within-class residual degrees of freedom; these cells are reported as missing, not as zero performance.

For smoothed-reflectance CORAL, ten fixed outer split IDs were used to compare accession-balanced subsets with unbalanced subsets selected by simple random sampling. Subsets contained 32, 64 or 160 target-development seeds, with five draws per size and split. Numerical covariance rank and ridge-regularised condition number were recorded. With 32 balanced target seeds, a one-factor sensitivity analysis varied the covariance ridge and retained PCA variance. All target test pairs remained excluded (Supplementary Method S1.14; Table S21).

Source development spectra, target calibration size and held-out test pairs were fixed within each comparison. Each repeated control comprised 150 mappings per representation and direction. These algorithmic perturbations quantify sensitivity to calibration assignments within the studied material (Supplementary Methods S1.4, S1.9 and S1.13; Tables S12 and S18).

Additional calibration comparators were fitted without using held-out target seeds. For smoothed reflectance, a global gain/offset map and a per-band target-to-source location/scale map used development/calibration spectra. A paired ridge direct-standardisation (DS) comparator, motivated by the classical DS family [15,26], regressed the full source spectrum on the paired target spectrum with an unpenalised intercept and a ridge penalty of 10^−4^; it used all 70 physical pairs per accession and then reused the source-trained classifier. Ridge values 10^−6^ and 10^−2^ were examined descriptively. The DS map uses all 101 spectral coordinates jointly, whereas the primary PDS map uses a local ±10 nm window. Target-only and pooled labelled-target PCA–LDA were evaluated at the same nested target budgets for reflectance and SNV; their use of target labels differs fundamentally from CORAL’s alignment objective (Supplementary Method S1.10).

A one-factor-at-a-time sensitivity analysis used ten fixed pair-preserving split IDs and varied PDS half-window (1, 2 or 4 common-grid steps), PDS ridge (10^−8^, 10^−6^ or 10^−4^), CORAL ridge (10^−6^, 10^−4^ or 10^−2^), and PCA retained variance (99.0%, 99.5% or 99.9%). Transformations and classifiers were fitted on development/calibration spectra and then evaluated on held-out test seeds; test results were not used to choose a winning setting. The fixed reference settings provide the baseline for this sensitivity analysis (Supplementary Method S1.9; Table S13).

### 2.8. Fixed-Split Paired-Bootstrap Inference

One pair-preserving 70:30 seed-index split, generated with random seed 20,960,726, was applied across representations, directions, and the transfer methods. It contained 2240 development/calibration seeds (70 per accession) and 960 test seeds (30 per accession). Both spectra from each held-out physical seed were excluded from model fitting and adaptation, and target test labels were used only for evaluation. Inference is conditional on this fixed partition and the studied accession–lot and acquisition setting; repeated splits are descriptive and not external validation.

Uncertainty was estimated using 4999 paired stratified bootstrap samples [38]. Within each replicate, the 30 test seeds were sampled with replacement separately within each accession, using identical indices for the methods being compared. Scenario-specific macro-F1 intervals were 95% percentile intervals. Twelve fixed contrasts compared PDS and CORAL with direct transfer across three representations and two directions. Two-sided plus-one *p* values came from null-centred paired-bootstrap distributions; their minimum attainable value was 1/(4999 + 1) = 0.0002, and Holm adjustment covered the 12 contrasts. Exact values remain in Table S8; the main presentation emphasises effect sizes and intervals. The bootstrap is conditional on one calibration set and excludes calibration-set, scan, lot, day and instrument-unit variability. Separately, the differences across the 30 overlapping splits are reported as distributions with descriptive central 95% ranges (Table S20), not independent-sample confidence intervals.

The fixed-split pairing analysis used one fixed-point-free within-accession derangement of the 2240 calibration spectra. Paired-minus-deranged PDS differences were summarised with paired-bootstrap 90% confidence intervals for an exploratory comparison with a symmetric ±0.010 macro-F1 band, and 95% intervals were used for performance and transfer gains. The 90% level follows the interval convention for two one-sided 5% comparisons with a band; here, the band was an illustrative sensitivity threshold, not a prospectively justified scientific equivalence margin. Intervals wholly inside it indicate satisfaction of that illustrative threshold, not practical equivalence. The intervals are conditional on one development/calibration partition and one derangement and omit uncertainty due to differences in calibration sets, lots, days and instruments.

Bootstrap and derangement seeds were 20,960,727 and 20,960,728, respectively; exact formulae are provided in Supplementary Method S1.4.

### 2.9. Sensitivity to Numerical Support and Sampling Density

Smoothed-reflectance transfer was repeated on four numerical grids nested within the primary common interval: 450–950 nm at 5 nm (101 coordinates), 450–900 nm at 5 nm (91), 450–950 nm at 10 nm (51), and 450–900 nm at 10 nm (46). The same 30 main paired splits were used. For PDS, a half-window of two grid points on 5 nm grids and one grid point on 10 nm grids maintained an approximately ±10 nm local span. The 450–950 nm interval was a conservative overlap of the two measured wavelength ranges, and the 5 nm increment was a numerical analysis choice following approximately 15 nm native-axis smoothing. These tests assess sensitivity to numerical coordinate support and density. Optical spectral-response differences are considered separately in Section 4.4.

The numerical-grid reference was 450–950 nm at 5 nm spacing; full definitions and results are provided in Supplementary Method S1.4 and Table S9.

### 2.10. Compact Wavelength Panels

Sparse PLS-DA provides a supervised feature-selection approach for multiclass discrimination [39]. Compact-panel analyses used smoothed reflectance and separate pair-preserving 70:30 splits. Four feature sets were compared: all 101 bands; 16 approximately uniformly spaced bands; 16 source-discriminative bands ranked by accession eta-squared with at least 10 nm separation; and a 16-band transportability panel. The latter combines cross-sensor accession discrimination (Equation (6)), domain stability (Equation (7)) and non-negative accession-centroid correlation (Equation (8)) using an equal-weight harmonic mean (Equation (9)) and then selects the highest score within each of 16 equal spectral regions (Equation (10)). Sixteen bands are an exploratory compactness budget, and equal weights prevent one weak component from being hidden by a large arithmetic sum. Known target-calibration accession labels enter this score, whereas the source-ranked and uniform panels have less supervision. Target test labels are excluded, and PDS/CORAL are refitted using only selected bands. A classification-compatible minimum-redundancy maximum-relevance (mRMR) comparator [40] uses the same labelled source and target-calibration samples, and 8- and 32-band sensitivities supplement the primary 16-band comparison (Supplementary Method S1.15; Table S24).

Within-accession residual paired correlation and Lin’s concordance correlation coefficient were recorded as diagnostics but did not enter the transportability score.

The score components and wavelength-selection rule are defined as follows:

(6) $$D_{j}=\sqrt{\eta_{S,j}^{2}\eta_{T,j}^{2}}$$

(7) $$\Delta_{j}=\frac{\left|{\bar{x}}_{S,j}-{\bar{x}}_{T,j}\right|}{\sqrt{\frac{{s_{S,j}}^{2}+{s_{T,j}}^{2}}{2}}},U_{j}=e^{-\Delta_{j}}$$

*C_j_* = max(0,cor*_a_*(*μ*_S,*a*,*j*_,*μ*_T,*a*,*j*_)) (8)

(9) $$H_{j}=\frac{3}{{\max\left(D_{j},10^{-8}\right)}^{-1}+{\max\left(U_{j},10^{-8}\right)}^{-1}+{\max\left(C_{j},10^{-8}\right)}^{-1}}$$

(10) $$\omega^{m_{*}}=\underset{\omega_{j}\in R_{m}}{\mathrm{arg max}}H_{j},m=1,\ldots ,16$$

In Equations (6)–(10), S and T denote source development and target calibration, eta-squared is the between-accession fraction of spectral variance, and each score component is bounded below by 10^−8^ before harmonic aggregation. The marginal shift is scaled by the corresponding pooled standard deviation. *C_j_* is the non-negative correlation between the two sets of accession centroids at wavelength *j*, not an individual-seed correlation. In Equation (10), the selected wavelength maximises the score within spectral region *m*, for *m* = 1, …,16. For reduced-panel PDS, the *h* = 2 window is defined over adjacent selected columns rather than a fixed physical wavelength interval.

### 2.11. Probability Calibration, Prediction Sets, and Selective Classification

As an exploratory extension of Q3, we assessed whether adaptation gains were accompanied by more reliable probabilities and selective predictions. Smoothed-reflectance analyses used 30 pair-preserving 50/20/30 splits per accession: 50 source seeds for fitting and paired target adaptation, 20 source seeds for conformal calibration, and 30 seeds for evaluation. Calibration and test pairs were excluded from adaptation. This separate partition reserves data for probability calibration and is not pooled with the 70:30 classification results. We report expected calibration error (ECE), selective accuracy, 90% prediction-set coverage and an exploratory importance-weighted variant, with variability across splits (Supplementary Method S1.6; Tables S6 and S25). Coverage is empirical under domain shift, not a deployment guarantee [31,38,41].

### 2.12. Scope of Validation

Validation used held-out seed pairs from the studied accession–lots, with both spectra of each test seed excluded from fitting and adaptation. Each seed was imaged once per camera, so the split-half benchmark measures variation among different seeds rather than within-sensor repeatability. Optical sampling and radiometric processing were not varied independently. One lot per accession and same-day measurements with one unit of each camera delimit population, temporal and instrument-unit generalisation.

### 2.13. Software and Reproducibility

Analyses were performed in R 4.5.2 using RStudio 2026.04.0 (Posit Software, PBC, Boston, MA, USA). Packages included readxl, data.table, matrixStats, prospectr, vegan, MASS, glmnet, ggplot2 and their dependencies. Figures were generated in R and Python with Matplotlib. Package versions, parameter grids and random seeds are reported in Table S10 and Supplementary Methods S1.7–S1.17; Tables S11–S26 provide the additional comparisons and sensitivity results. Specified settings and contrasts describe the analytical choices used here.

## 3. Results

### 3.1. Numerical Alignment Did Not Remove Domain Separation

After smoothing and resampling, each sensor–representation matrix contained 3200 spectra and 101 finite features (Figure S1; Table S1).

The common numerical grid made the matrices dimensionally compatible but did not remove all observable domain separation. In smoothed reflectance, the held-out linear-domain classifier gave a mean AUC of 0.822 ± 0.006 before adaptation. After PDS or CORAL, AUC was exactly 0.500 because all fitted classifiers produced constant scores (Table S22). Alignment of development-domain means made this linear diagnostic uninformative; it does not establish the absence of nonlinear or class-conditional shift. The internal cross-validation values of 0.483–0.485 followed alignment fitted before the internal folds (Figure S3; Table S6) and are not independent estimates of held-out alignment quality.

### 3.2. Accession–Lot Geometry and Within-Accession Spectral Retrieval

**Figure 2:**
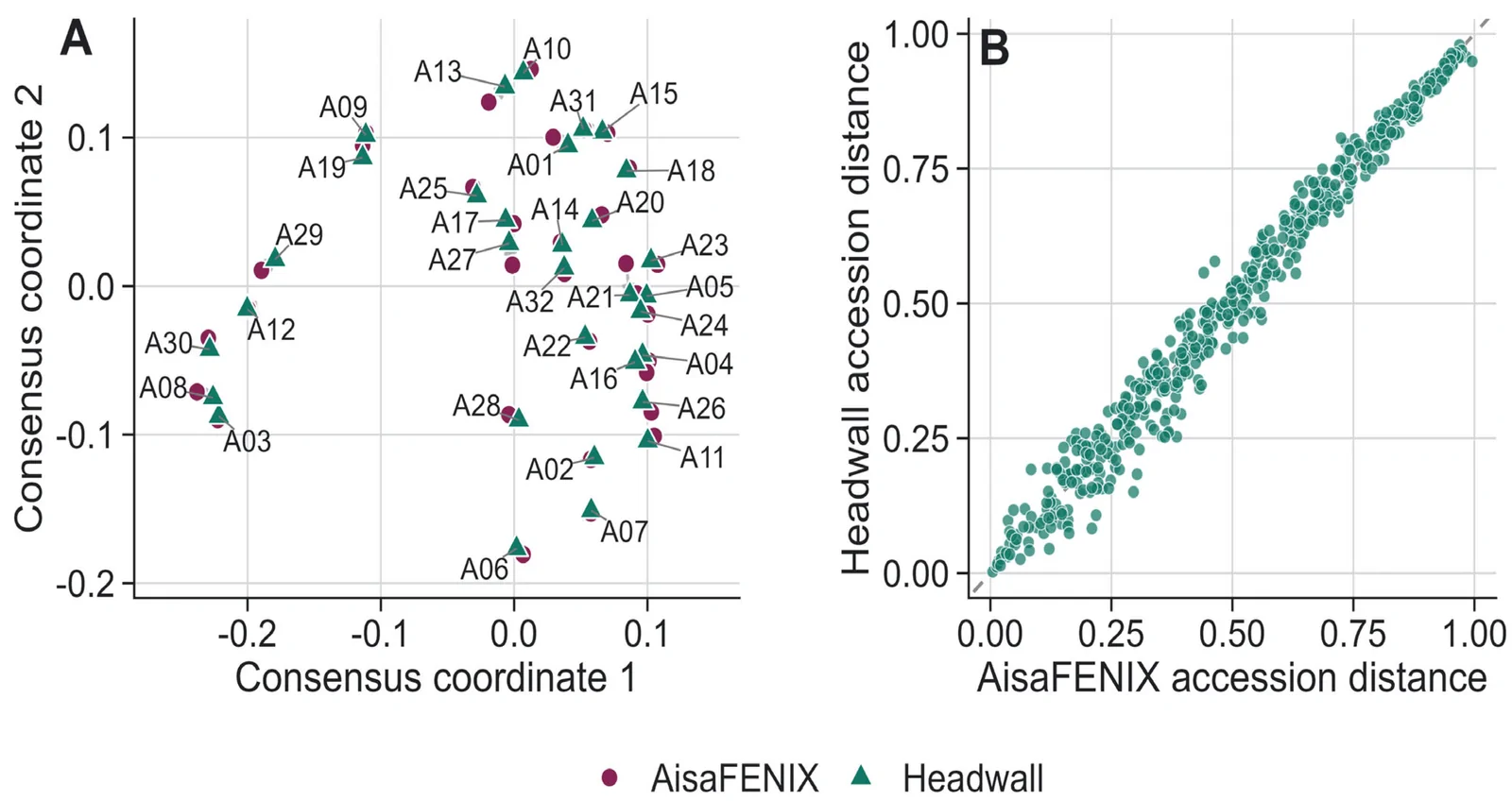
Conservation of accession-level spectral geometry across instruments. (**A**) Accession centroids after symmetric Procrustes alignment of consensus rank-distance configurations. All 32 accession–lot pairs are shown. Arrows connect AisaFENIX to Headwall centroids, and labels identify A01–A32 in the displayed two-dimensional projection. Each centroid summarises 100 seeds; the arrows are not individual-seed links. (**B**) Concordance of all 496 pairwise accession distances. Consensus rank-distance agreement was Mantel rho = 0.989 and Procrustes r = 0.981 (999 permutations).

Accession–lot organisation was highly similar between instruments (Figure 2; Table S2). Mantel rho and Procrustes r were 0.958 and 0.981 for smoothed reflectance, 0.996 and 0.997 for SNV, and 0.982 and 0.979 for first derivatives. Paired seed-bootstrap 95% intervals for Mantel rho were 0.943–0.964, 0.994–0.996 and 0.977–0.983, respectively (Table S17). These intervals are conditional on the observed accession–lots. The composite rank-distance analysis summarised the three representations, giving Mantel rho = 0.989 and Procrustes r = 0.981 (each permutation *p* = 0.001). Accession-centroid top-1 retrieval ranged from 31.25% to 81.25% across representations and directions, above the 3.125% chance expectation but well below perfect matching despite high global distance-matrix correlation (Table S2B).

The 30 split-half divisions supported stable accession-level geometry across disjoint groups of seeds (Table S11). Mean within-AisaFENIX, within-Headwall, and matched-half cross-sensor Mantel correlations were 0.985, 0.988, and approximately 0.953 for reflectance; 0.997, 0.997, and approximately 0.995 for SNV; and 0.995, 0.995, and approximately 0.980 for derivatives. In the separate common-centroid PCA sensitivity analysis, removing PC1 reduced cross-sensor Mantel correlation from 0.962 to 0.944 for reflectance, 0.996 to 0.987 for SNV, and 0.985 to 0.960 for derivatives. Excluding the two non-Phaseolus accessions left cross-sensor correlations of 0.956, 0.997, and 0.984, respectively (Tables S11 and S16). The PCA sensitivity analysis uses joint centroid scaling, whereas the primary geometry calculation uses within-sensor scaling.

Within-accession retrieval by spectral correlation ranged from 0.81% to 1.44% across the six representation-by-residualisation settings, compared with a 1% random-selection expectation (Figure S2). For smoothed reflectance, it was 0.84% from AisaFENIX to Headwall and 1.06% in reverse before residualisation, and 1.16% and 1.44% after residualisation. None of the six mean-paired-similarity permutation tests provided evidence against the within-accession null (*p* = 0.143–0.840). These results describe individual retrieval by the specified correlation criterion, distinct from predicting accession labels.

For seed mean reflectance and PC1 scores, median within-accession cross-sensor correlations were −0.016 and −0.008, respectively (permutation *p* = 0.4700 and 0.7084). The full results, including the offset and reversed-order sensitivity controls, are reported in Table S26.

### 3.3. Direct Transfer Reduced Macro-F1, and Recovery Depended on Direction and Representation

Across 30 pair-preserving but overlapping splits, mean within-sensor PCA–LDA macro-F1 ranged from 0.728 to 0.917, whereas mean direct-transfer macro-F1 ranged from 0.367 to 0.646 (Table 2). Relative to the source PCA–LDA reference, direct-transfer losses were 25.7, 17.6 and 53.8 macro-F1 percentage points for reflectance, SNV and derivatives from AisaFENIX to Headwall and 38.2, 22.3 and 24.3 points in reverse. The within-sensor values are held-out seed-level references within the studied accession–lots. For reflectance, direct-transfer macro-F1 was 0.471 from AisaFENIX to Headwall and 0.367 in reverse; PDS/CORAL increased these values to 0.674/0.686 and 0.522/0.674. For SNV, CORAL was numerically higher than PDS in the first direction (0.733 versus 0.707), whereas PDS was numerically higher in reverse (0.834 versus 0.770). First derivatives combined the highest AisaFENIX within-sensor reference (0.917) with the lowest AisaFENIX-to-Headwall direct result (0.379). Between-pipeline differences were not formally tested. SDs across overlapping splits were 0.008–0.018 and are descriptive.

**Figure 3:**
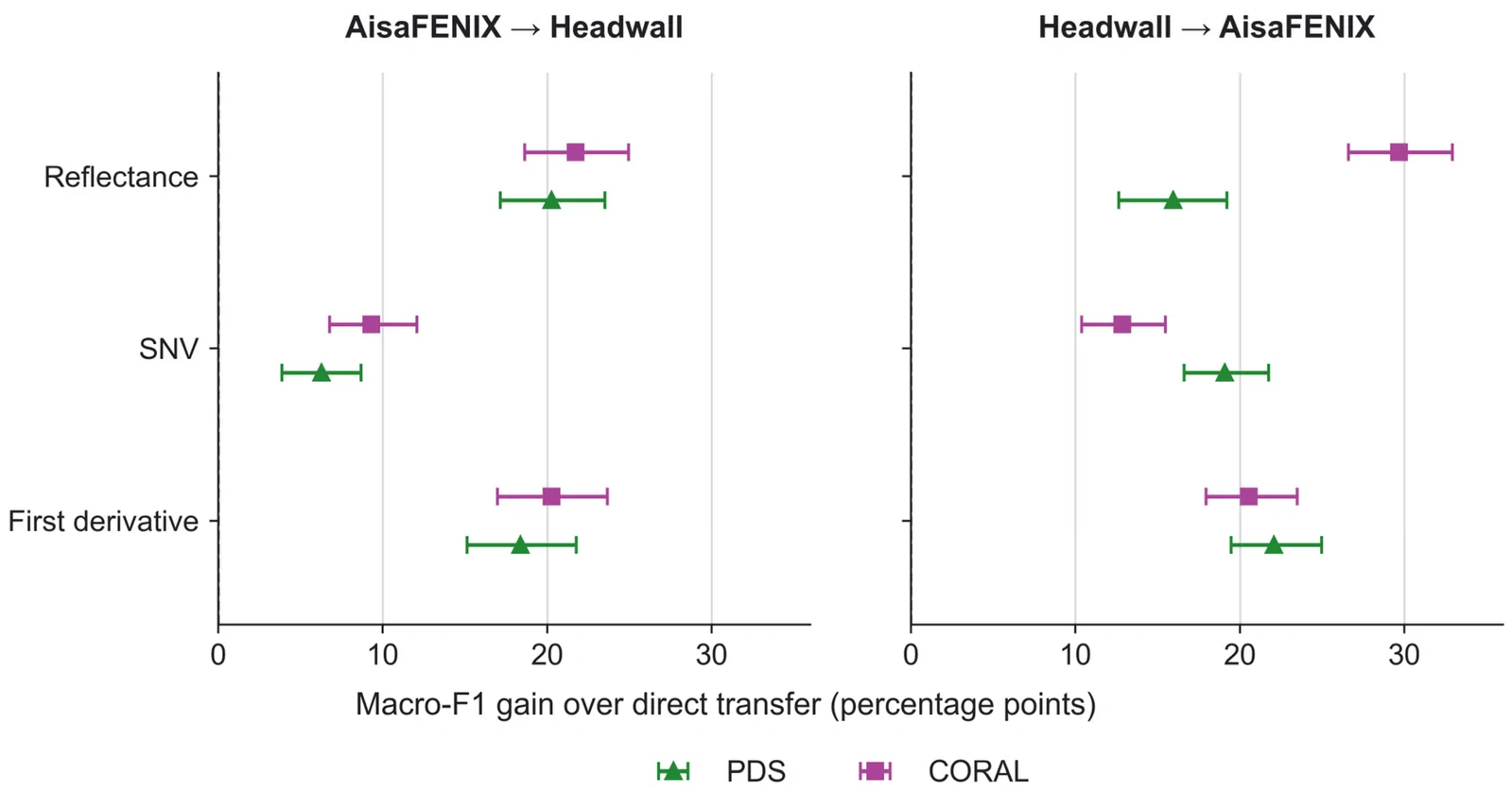
Fixed-split cross-sensor transfer gains. Points show the observed macro-F1 difference between PDS or CORAL and direct transfer in both directions for smoothed reflectance, SNV and first derivatives. Bars are stratified paired-bootstrap 95% confidence intervals based on 4999 resamples of the same 960 held-out test seeds. These intervals are conditional on one development/calibration partition and do not include variation from alternative calibration sets, scanning sessions, lots, days or instrument units. Absolute performance over the 30 overlapping splits is reported in Table 2.

On the fixed split, all 12 specified PDS/CORAL-versus-direct contrasts were positive (Figure 3; Table 3; Figure S8A). PDS-minus-direct gains ranged from 6.29 to 22.06 macro-F1 percentage points, and CORAL-minus-direct gains ranged from 9.30 to 29.67 points. Paired-bootstrap 95% confidence intervals excluded zero. With 4999 replicates, the observed raw plus-one *p* values reached the attainable minimum of 0.0002, and Holm-adjusted *p* values were 0.0024 (Table S8). These tests are conditional on the fixed seed-random partition and do not compare PDS with CORAL. Across 30 overlapping partitions, paired-gain distributions are reported separately as descriptive means, SDs and empirical central 95% ranges (Table S20).

Additional full-calibration comparators are reported in Table 2 and Table S14. A paired ridge DS map yielded mean macro-F1 of 0.718 and 0.755 for reflectance, 0.743 and 0.874 for SNV, and 0.752 and 0.867 for derivatives, with AisaFENIX-to-Headwall transfer first in each pair; all 70 paired standards per accession were used. DS achieved higher mean macro-F1 than PDS and CORAL in all six combinations. Its largest gain over PDS was 23.3 percentage points for reverse reflectance; the AisaFENIX-to-Headwall derivative gain was 17.15 points. In reflectance, per-band location/scale alignment achieved 0.669 and 0.659, whereas global gain/offset alignment achieved 0.579 and 0.512. Shrinkage LDA gave within-sensor reflectance references of 0.843 for AisaFENIX and 0.838 for Headwall, compared with 0.728 and 0.749 for PCA–LDA (Table S19). These descriptive comparisons contextualise the fixed PCA–LDA transfer benchmark; transfer with shrinkage LDA was not evaluated.

### 3.4. Calibration Demand and Pairing Value Were Setting Specific

Across 30 overlapping splits and five within-accession fixed-point-free derangements per split, mean exact-minus-deranged PDS macro-F1 differences ranged from −0.0012 to +0.0121 across the six representation–direction settings (Table S12). The fixed split with one derangement gave 90% intervals wholly within the illustrative ±1-percentage-point band in two settings, intervals crossing the band in three, and a negative derivative contrast in one (Figure S8B; Table S8). The illustrative band and dependence on one partition and derangement make this an exploratory comparison of calibration-pair sensitivity.

When every calibration accession was deliberately mismatched to another accession, mean PDS macro-F1 fell to 0.004–0.010 across the six representation–direction combinations (Table S18). This stronger negative control destroys class correspondence, unlike within-accession derangement. It demonstrates the dependence of PDS performance on accession-matched calibration.

**Figure 4:**
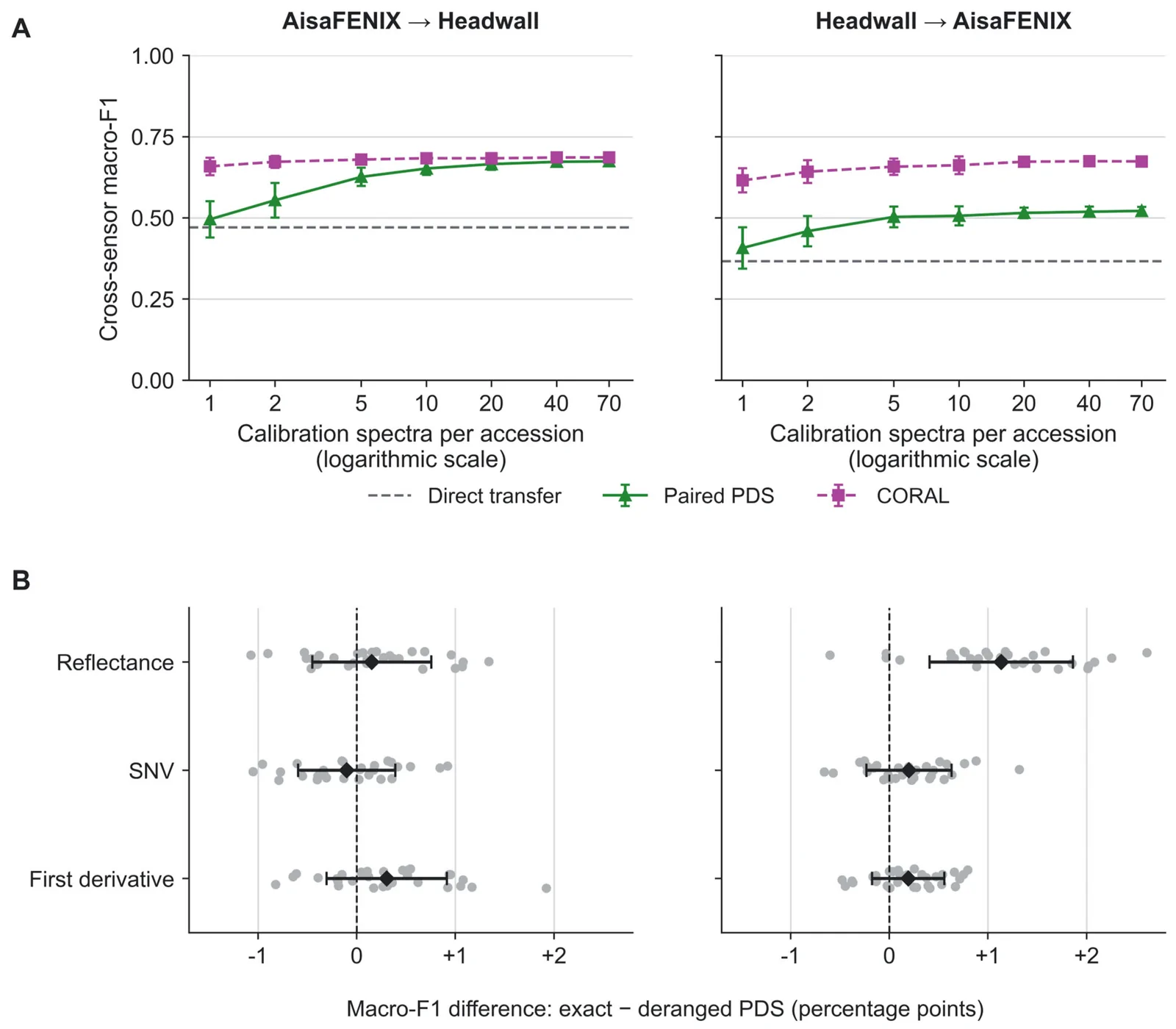
Reflectance calibration demand and effect of within-accession derangement. Columns consistently identify the transfer direction. (**A**) Mean macro-F1 as a function of the number of accession-balanced target-calibration spectra per accession on a logarithmic horizontal scale. Green triangles represent PDS, purple squares represent CORAL, and the dashed grey line represents direct transfer; bars show descriptive SD across 30 overlapping splits. Panel selection required known accession labels; PDS used physical pairs, whereas CORAL did not use pair identities. (**B**) Paired-minus-one-derangement PDS macro-F1 differences for the same splits. Grey points are split-specific estimates; black diamonds and bars show means and descriptive SD, not confidence intervals. Five-derangement-per-split and cross-accession controls are reported in Tables S12 and S18. In panel B, ‘exact’ denotes calibration using the same physical seeds measured by both cameras.

Calibration demand depended on the pipeline and available target supervision (Figure 4A; Tables S4 and S15). With one accession-balanced target reflectance spectrum per accession, CORAL macro-F1 rose from 0.471 to 0.659 for AisaFENIX-to-Headwall transfer and from 0.367 to 0.615 in reverse; PDS improved more gradually to 0.674 and 0.522 at 70 pairs per accession. This small panel was selected using known accession labels, although CORAL’s covariance objective did not use labels, and its centred covariance rank could not exceed 31. The extended SNV budget analysis and target-only/pooled labelled comparators are reported in Table S15. At 70 labelled target spectra per accession, target-only/pooled macro-F1 was 0.749/0.681 and 0.728/0.639 for reflectance and 0.852/0.831 and 0.822/0.871 for SNV in the two directions. No target-only value was estimated at one sample per accession because PCA–LDA had no within-class residual degrees of freedom.

In the additional composition experiment, 32 balanced target spectra gave CORAL macro-F1 of 0.655 and 0.618, compared with 0.510 and 0.496 for subsets of the same size selected by simple random sampling. The balanced estimates differ slightly from the 0.659/0.615 budget-curve values because this comparison used ten outer splits with five panel draws averaged within each split, whereas the budget curves used 30 splits with one draw. Random panels represented a mean of 20.4 of the 32 accessions. At 160 spectra, balanced/random values were 0.682/0.647 and 0.652/0.615 (Table S21). With 32 balanced spectra, changing retained PCA variance from 99.0% to 99.9% changed macro-F1 from 0.594 to 0.716 and from 0.596 to 0.692. The small-budget result therefore applies to accession-balanced panels under the evaluated regularisation settings.

A one-factor sensitivity analysis on ten specified splits showed that calibration settings affected some pipeline estimates (Table S13). For example, reflectance AisaFENIX-to-Headwall CORAL macro-F1 ranged from approximately 0.624 to 0.756 across its tested settings, reverse reflectance PDS ranged from 0.486 to 0.575, and first-derivative AisaFENIX-to-Headwall PDS ranged from 0.522 to 0.634. These ranges show that the reported performance depends on the chosen calibration and PCA settings.

### 3.5. Numerical Support and Compact Panels

Transfer performance depended on numerical wavelength support, sampling density and smoothing span, with direction- and method-specific effects (Supplementary Results S2.1; Tables S9 and S23; Figure S9).

Compact wavelength panels, including the transportability-oriented and mRMR selections, did not improve transfer consistently across directions and methods (Supplementary Results S2.2; Tables S5 and S24; Figure S10).

### 3.6. Probability Reliability Remained Conditional on the Measurement Domain

Across the 30 exploratory 50/20/30 reflectance splits, direct-transfer ECE was 0.296 ± 0.016 from AisaFENIX to Headwall and 0.365 ± 0.015 in reverse (Figure S6; Table S25). PDS reduced these means to 0.041 ± 0.011 and 0.166 ± 0.015, and CORAL reduced these means to 0.054 ± 0.009 and 0.082 ± 0.010. At 50% retention, selective accuracy for direct transfer, PDS and CORAL was 0.668 ± 0.020, 0.855 ± 0.015 and 0.865 ± 0.009 from AisaFENIX to Headwall and 0.503 ± 0.022, 0.656 ± 0.019 and 0.826 ± 0.013 in reverse. At a nominal coverage level of 90%, source-calibrated target coverage was 0.679 ± 0.023 and 0.559 ± 0.030 for direct transfer, 0.910 ± 0.015 and 0.751 ± 0.026 for PDS, and 0.900 ± 0.017 and 0.844 ± 0.019 for CORAL. Worst-accession mean coverage remained below 0.90 (Figure S7). For direct transfer, importance weighting changed coverage by −0.014 ± 0.015 from AisaFENIX to Headwall and +0.037 ± 0.054 in reverse; adapted weighted and unweighted values coincided because the fitted linear-domain scores were constant. These SDs and the empirical ranges in Table S25 summarise variation across overlapping partitions of the studied seeds.

### 3.7. Geometry, Calibration, and Taxonomic Sensitivities

Further sensitivity analyses examined accession composition, wavelength range and classifier choice (Tables S11–S25). Excluding A27 and A28 retained high geometric association and positive mean adaptation gains, but some classification estimates shifted by approximately 0.046. In the full collection, reflectance geometry correlation was 0.978 over 450–700 nm and 0.906 over 700–950 nm; SNV correlations were 0.995 and 0.993, and derivative correlations were 0.973 and 0.961 (Table S17). With a fixed shrinkage parameter λ = 0.1, within-sensor LDA macro-F1 was 0.843/0.838 for reflectance, 0.929/0.867 for SNV and 0.926/0.851 for derivatives, with AisaFENIX first in each pair (Table S19). Together, these descriptive sensitivities distinguish relative geometry, classifier choice and portability within the studied accession–lots and acquisition setting.

## 4. Discussion

### 4.1. Conserved Accession–Lot Structure Did Not Imply Seed-Level Spectral Retrieval or Model Portability

The results distinguish relative accession–lot geometry, individual spectral retrieval and accession-classifier transfer. Wavelength standardisation within each sensor removes per-band location and scale before the centroid distances are calculated. High geometric agreement can therefore coexist with direct-transfer losses that are reduced by calibration of those differences. Individual retrieval asks a separate question: whether spectral correlation selects the same seed among 100 candidates from one accession. For complete smoothed-reflectance profiles, mean cross-sensor correlation was 0.99487 for the same seed and 0.99482 for different seeds within the same accession (Table S2A). The high similarity of other seeds’ profiles can limit unique retrieval without preventing accession classification, which requires discrimination between accessions. The paired acquisition retained the same seed positions, exposed faces and orientations during same-day camera exchange.

Accession-centroid aggregation can suppress seed-level spectral variability. The split-half benchmark shows high accession-distance stability within each sensor (reflectance Mantel r = 0.985 for AisaFENIX and 0.988 for Headwall) and a smaller cross-sensor value (approximately 0.953); analogous SNV and derivative comparisons were 0.997/0.997 versus approximately 0.995 and 0.995/0.995 versus approximately 0.980. These geometry checks compare disjoint groups of seeds. Removing a shared PC1 reduced cross-sensor geometry correlation but left substantial structure, so a single broad axis does not fully account for the association. Centroid top-1 retrieval was as low as 31.25%, illustrating that global distance agreement and centroid matching capture different features of cross-system similarity. With one observed lot per accession, the geometry describes these accession–lot combinations.

The present common 450–950 nm comparison fixes one classifier family to make transfer contrasts interpretable. The pooled-covariance shrinkage-LDA results (Table S19) confirm that stronger within-sensor performance is attainable under some alternative settings, showing how the reference performance depends on classifier choice. Successful transfer of single-seed soybean trait calibrations between sister NIR analysers [19] and the gains obtained here both support explicit calibration across instruments while addressing different prediction endpoints and spectral intervals.

This distinction is relevant to the shift from closed-domain proof of concept to more credible seed-imaging evaluation. Studies increasingly address broader classification and quality tasks [42,43,44,45], real-world data, self-supervision, and open-set recognition [8,9,46]. A model can remain stable under internal resampling yet fail when the measurement system changes.

### 4.2. Directional Asymmetry Was Consistent with Method-Specific Objectives

Within the specified PCA–LDA pipelines, PDS and CORAL improved performance in all 12 fixed-split comparisons with direct transfer. Descriptive rankings varied with transfer direction and spectral representation; there was no formal PDS-versus-CORAL test. Full-grid paired ridge DS achieved higher mean macro-F1 than both across all six representation–direction combinations. The global DS and local ±10 nm PDS maps differ in spectral neighbourhood and regularisation. The choice of adaptation pipeline must be evaluated jointly with calibration effort, target-label access, and mapping architecture. These transfer findings concern PCA–LDA; shrinkage LDA was evaluated as a within-sensor reference.

Distinct pipeline objectives offer a possible explanation for the directional asymmetry. PDS predicts source-coordinate features from paired target-coordinate spectra [26] and then reuses the source-trained classifier; reverse transfer changes the regression target and residual structure. CORAL aligns source and target second-order statistics [27]; in the present pipeline, it maps source development statistics to target calibration statistics and refits PCA–LDA after alignment. Consequently, the PDS–CORAL comparison changes mapping direction, supervision, and classifier refitting simultaneously. SNV attenuates spectrum-wise additive and multiplicative variation [35], whereas the Savitzky–Golay derivative representation emphasises local spectral change [34]. The AisaFENIX derivative reference of 0.917 fell to 0.379 under direct transfer to Headwall, illustrating that strong within-sensor separation need not persist in the other sensor’s feature space. Camera optics, spatial and spectral sampling, radiometric calibration and sensor-specific ROI averaging can all contribute even when corresponding central seed-coat regions are sampled. Their separate effects were not isolated here. The one-factor parameter sensitivity analysis further showed that performance depends on the window, regularisation and PCA settings.

For SNV Headwall-to-AisaFENIX transfer, ridge DS and pooled training achieved macro-F1 values of 0.874 and 0.871, compared with source and target PCA–LDA references of 0.852 and 0.822. These references describe individual single-sensor fits rather than upper bounds: DS estimates a new target-to-source representation, while pooled training uses labelled development spectra from both sensors. Both spectra from every held-out seed were excluded from these fits. Higher performance than a single-sensor PCA–LDA reference is therefore compatible with the different training information and transformations.

Multi-instrument calibration studies use local projections, interpretable deep mappings and transfer between portable spectrometers and imaging systems [47,48,49,50]. Cross-scene hyperspectral methods include adversarial and confidence-based adaptation [51,52,53], feature compaction, gradients and locality preservation [54,55,56]. NIR calibration work also includes parameter-free enhancement and principal-component extreme-learning mappings [57,58], as well as deep and regularised-kernel transfer [59,60]. These studies motivate alternative alignment strategies but address different endpoints and domains. P-norm attention Deep CORAL is a nonlinear covariance-alignment extension evaluated on object-image domains [61]. Together, these studies provide methodological options for future tests under the paired evaluation design used here.

### 4.3. Calibration Composition and Individual Seed Pairing

The pairing control compared calibration using the same seeds across cameras with alternative assignments preserving accession composition. Five derangements in each of 30 overlapping splits yielded small average paired-minus-deranged differences, from −0.0012 to +0.0121 macro-F1, across the six settings; the largest positive value occurred for Headwall-to-AisaFENIX reflectance. A single fixed-split negative first-derivative contrast did not persist as a consistently negative average across derangements and splits. The ±1-percentage-point interval rule was illustrative rather than a justified equivalence margin.

PDS was more sensitive to mismatching accessions than to substituting different seeds from the same accession in this calibration population. This supports the importance of accession composition for the tested mapping, while the small effects of within-accession derangement depend on representation and direction. Optical geometry, detector response, radiometric processing and sensor-specific ROI averaging were not varied separately.

Calibration demand depends on target-sample composition. The smallest balanced CORAL panel contains one known accession standard per class and has a target-covariance rank of at most 31 across 101 features. The random-subset comparison shows the benefit of balancing accessions at a fixed number of target spectra, while the retained PCA variance also changes performance (Table S21). This result concerns selection of labelled accession standards. Source development and target calibration used the same seed population, a favourable setting compared with deployment on unrelated lots. Target-only and pooled labelled-target models provide separate supervision comparators, and full-budget ridge DS uses all 70 physical pairs per accession.

The full-budget comparators further clarify the role of method choice. Per-band reflectance location/scale matching produced gains over direct transfer in both directions, indicating that some domain shift can be reduced by simple calibration, while paired ridge DS was numerically the strongest tested full-budget paired mapping across all six representation–direction cells. The numerical ranking compares pipelines with different supervision and architectures under the reported calibration budgets and remains descriptive. A controlled comparison with matched calibration budgets and supervision would help isolate differences between the mapping methods.

The repeated balanced-versus-random comparison directly tests calibration composition within the observed development pools. Future operational studies should extend it to diversity-based selection, genuinely unlabelled deployment streams, new lots and acquisition conditions. Standard-free and standard-guided transfer strategies provide relevant methodological precedents [62,63,64]. These extensions would test whether the present controlled-standard advantage persists beyond the studied accession collection.

### 4.4. Numerical Spectral Support and Compact Panels

The common 101-coordinate grid over 450–950 nm provides numerical compatibility despite the different native spectral ranges and channel counts. Native-axis smoothing attenuates fine spectral structure. The effective response of each pipeline depends on the instrument response and smoothing kernel; the smoothing window, output spacing and instrumental FWHM therefore describe distinct properties. Acquisition-specific response curves were unavailable, and manufacturer values supply model-family specifications (Table 1). The 5/15/25 nm target-span sensitivity analysis tests processing dependence (Table S23).

Related optical and numerical distinctions arise in satellite harmonisation: spectral-bandpass adjustment and atmospheric-response effects [65,66], learned reflectance harmonisation and cross-sensor self-supervision [67,68], and surface-reflectance cross-comparison and missing-band prediction [69,70]. These studies provide methodological analogies for spectral harmonisation at satellite scales. In this study, changing numerical support altered some transfer results, partly through the PDS neighbourhood definition. Further characterisation of the cameras’ spatial and spectral responses would help distinguish optical effects from processing effects.

Compact panels exposed a distinction between source discrimination and transportability, but all panel analyses here used smoothed reflectance. The 16 source-univariate-ranked bands yielded weak performance even within the source sensor (macro-F1 0.431 and 0.513), so their poor target performance cannot be assigned solely to domain shift. The transportability score preserved AisaFENIX-to-Headwall CORAL macro-F1 but not that of reverse CORAL or AisaFENIX-to-Headwall PDS, and it used known target-calibration accession labels. Uniform spacing was more consistent. Neither within-source univariate ranking nor a label-informed two-domain score guaranteed a compact, bidirectionally portable panel. Inter-instrument wheat and maize kernel work likewise evaluates wavelength selection jointly with preprocessing and transfer algorithms [21], supporting the need to test a compact panel within its intended transfer pipeline.

Wavelength-selection methods serve different endpoints: CARS and sparse PLS-DA address regression and multiclass discrimination, respectively [28,39], while hybrid, interval, attention and multistage approaches have been developed for other spectra and images [29,71,72,73]. UAV-based leaf-area-index selection [74] illustrates the dependence of selected wavelengths on the biological target and measurement scale. Our mRMR comparator [40] uses the same available source and target-calibration labels as the transportability score, but the selection objectives still differ. The 8/16/32-band sensitivity analysis evaluates the selected panels descriptively under these criteria.

### 4.5. Uncertainty, Operational Implications, and Generalisation Boundaries

Domain shift affected confidence as well as classification. The exploratory 50/20/30 analyses show lower mean ECE and higher selective accuracy after adaptation, with split-to-split variability reported in Table S25. The target coverage of source-calibrated prediction sets nevertheless remained below the nominal 90% level in some directions and accessions. These findings show that improved mean ECE and selective accuracy can coexist with undercoverage under sensor shift. The separate conformal-calibration partition is necessary to reserve calibration observations; results from this partition should not be numerically pooled with those from the 70:30 transfer benchmark.

Ordinary conformal validity depends on exchangeability between calibration and test observations, which arbitrary domain shift can violate [31,75]. Risk-controlling and Jackknife+ constructions clarify different assumptions and error guarantees [41,76]. Cross-scanner pathology work using paired images of the same biopsies provides a relevant methodological example of validity failure after changing the imaging device [77]. In our held-out linear-domain check, constant post-alignment scores also illustrate a diagnostic limitation: aligned first moments can make this classifier uninformative, while nonlinear or class-conditional differences remain. Neither an AUC of 0.5 nor a small weighted–unweighted coverage difference proves removal of domain shift.

Hyperspectral processing software [78] and seed-imaging workflows [79] provide practical starting points for operational implementation. Further transfer studies should evaluate new lots and acquisition days under characterised optical and radiometric conditions. Environmental modelling of aerial corn imagery [80] offers a broader phenotyping example of acquisition dependence. Our same-day camera exchange controls seed position, orientation and temporal separation while leaving optical and processing differences combined. Calibration effort, target-label availability, compact panels, uncertainty and abstention therefore require testing for the intended application. Evaluating more flexible adaptation models [81] on independent lots and acquisitions would extend this work.

## 5. Conclusions

Analysis of the paired seed spectra showed stable relative structure of the observed accession–lots after within-sensor wavelength standardisation. Direct application of a source-trained PCA–LDA model to the other system produced large, direction-dependent losses despite common numerical wavelengths. Within-accession individual retrieval by spectral correlation was limited and served as a diagnostic distinct from accession classification.

PDS and CORAL improved performance in all 12 fixed-split PCA–LDA comparisons with direct transfer. Full-grid paired ridge DS achieved higher mean macro-F1 than both across all six representation–direction combinations. Repeated within-accession derangements had small, setting-dependent average effects, whereas mismatching accessions severely degraded PDS performance. These controls describe the contribution of calibration composition under the tested settings. Small-panel CORAL depended on accession-balanced standards and retained components. Numerical support, smoothing, compact-panel selection and probability reliability also varied by pipeline and direction.

Held-out seed-level validation supports the predictive comparisons within the studied accession–lots and imaging systems. Source development and target calibration share the same seed population. New lots, acquisition days and instrument units would extend the validation beyond this experiment, while repeated same-seed imaging and controlled optical characterisation would help separate measurement variability from other cross-system differences.

## Figures and Tables

**Figure 1:**
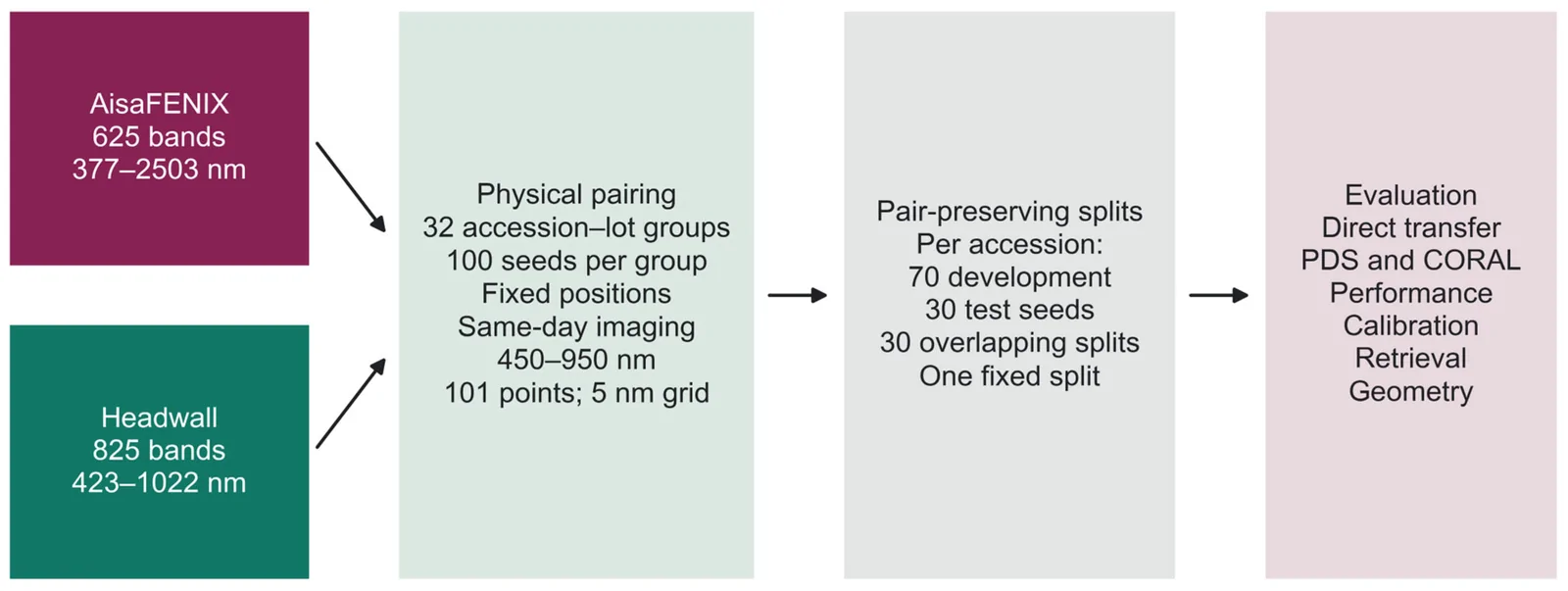
Same-day paired cross-instrument design and pair-preserving evaluation. The same 3200 distinct physical seeds from 32 accession–lot combinations (100 seeds per accession) retained their positions and orientation, while the imaging system was exchanged. One spectrum per seed and sensor was analysed. The numerical common grid contained 101 coordinates from 450 to 950 nm. Both measurements of each held-out seed were excluded from all development and adaptation steps. The main performance analysis used 30 overlapping pair-preserving splits; conditional bootstrap inference used one fixed split.

**Table 1:** Dataset, acquisition systems, pairing basis, and numerical wavelength alignment in AisaFENIX and Headwall hyperspectral cameras.

Characteristic	AisaFENIX	Headwall	Joint Analysis
Instrument	Specim AisaFENIX	Headwall Photonics A-Series	–
Native wavelength domain (nm)	377.27–2502.75	423.34–1022.07	–
Native channels	625	825	–
Median native sampling interval (nm)	1.72	0.73	–
Published model-family VNIR FWHM, not measured	3.5 nm mean	2.5 nm (25 µm nominal slit)	Published model-family specifications
Published model-family peak SNR, not measured	600–1000:1 (binning-dependent)	Not specified in cited A-Series datasheet	Empirical SNR unavailable
Acquisition-specific ground sampling distance	Not available	Not available	Physical pixel footprint not reported
Sensor-to-sample distance	85 cm	85 cm	Camera-specific optics and spatial resolution
Illumination and laboratory temperature	12 × 20 W halogen lamps; approximately 25 °C	12 × 20 W halogen lamps; approximately 25 °C	Same-day paired protocol
Timing and calibration	12 frames s−1; three calibration sessions	39 ms integration; 40 ms frame; four calibration sessions	Groups of 100 distinct seeds in fixed Petri-dish supports; one pass per group per camera; 3200 seeds per camera in total
ROI sampling and spectral averaging	10 interior pixels; central 10 × 10-pixel frame where feasible	All 100 pixels of the central 10 × 10-pixel ROI	One spectrum per seed from corresponding central interior seed-coat regions
Calibration and reflectance conversion	CaliGeoPRO 2.2; ENVI 5.4	ENVI 5.4	Dark and Spectralon white references
Seed spectra	3200	3200	–
Accessions	32	32	–
Seeds per accession	100	100	–
Missing or non-finite values	0	0	–
Values outside 0–1 within 450–950 nm	0	0	–
Pairing basis	Fixed seed position, face and orientation	Fixed seed position, face and orientation	Same 3200 physical seeds; same-day imaging; accession and fixed position define pairs
Primary common support	–	–	450–950 nm
Common analytical grid	–	–	101 grid points at 5 nm spacing
Spectral representations	–	–	Smoothed reflectance; SNV; first derivative
Pair-preserving evaluation	–	–	70 physical seeds for development and 30 for testing per accession; both spectra of each held-out seed excluded from fitting and adaptation

Note: FWHM and peak-SNR entries are published model-family values, not measured for these acquisitions; the analysed Headwall spectra contained 825 wavelength coordinates, whereas the cited A-Series sheet describes 837. Manufacturer sources: Specim AisaFENIX data sheet (https://www.specim.com/wp-content/uploads/2020/03/AisaFENIX-ver1-2020.pdf (accessed on 29 October 2026)); Headwall Hyperspec VNIR data sheet (https://integrys.com/wp-content/uploads/2016/08/VNIR.pdf (accessed on 29 October 2026)).

**Table 2:** Within-sensor classifier references, direct-transfer losses and bidirectional PCA–LDA transfer. Values are macro-F1 means ± descriptive SD across 30 overlapping pair-preserving splits. (**A**) PCA–LDA and shrinkage-LDA references, with source-reference-minus-direct loss in percentage points. (**B**) Transfer performance for the specified calibration methods.

**A. Within-Sensor References and Direct-Transfer Loss**						
**Direction**	**Spectral Input**	**Source PCA–LDA**	**Target PCA–LDA**	**Source Shrinkage LDA**	**Target Shrinkage LDA**	**Direct Loss (pp)**
Aisa → Headwall	Reflectance	0.728 ± 0.015	0.749 ± 0.011	0.843 ± 0.014	0.838 ± 0.010	25.7
Aisa → Headwall	SNV	0.822 ± 0.013	0.852 ± 0.009	0.929 ± 0.007	0.867 ± 0.009	17.6
Aisa → Headwall	First derivative	0.917 ± 0.008	0.851 ± 0.010	0.926 ± 0.007	0.851 ± 0.009	53.8
Headwall → Aisa	Reflectance	0.749 ± 0.011	0.728 ± 0.015	0.838 ± 0.010	0.843 ± 0.014	38.2
Headwall → Aisa	SNV	0.852 ± 0.009	0.822 ± 0.013	0.867 ± 0.009	0.929 ± 0.007	22.3
Headwall → Aisa	First derivative	0.851 ± 0.010	0.917 ± 0.008	0.851 ± 0.009	0.926 ± 0.007	24.3
**B. Cross-Sensor Transfer with PCA–LDA**						
**Direction**	**Spectral Input**	**Direct**	**PDS**	**CORAL**	**Ridge DS**	**Per-Band**
Aisa → Headwall	Reflectance	0.471 ± 0.011	0.674 ± 0.012	0.686 ± 0.013	0.718 ± 0.010	0.669 ± 0.013
Aisa → Headwall	SNV	0.646 ± 0.012	0.707 ± 0.014	0.733 ± 0.012	0.743 ± 0.012	—
Aisa → Headwall	First derivative	0.379 ± 0.012	0.580 ± 0.012	0.605 ± 0.015	0.752 ± 0.010	—
Headwall → Aisa	Reflectance	0.367 ± 0.009	0.522 ± 0.012	0.674 ± 0.012	0.755 ± 0.012	0.659 ± 0.013
Headwall → Aisa	SNV	0.629 ± 0.016	0.834 ± 0.011	0.770 ± 0.013	0.874 ± 0.010	—
Headwall → Aisa	First derivative	0.608 ± 0.018	0.813 ± 0.011	0.805 ± 0.013	0.867 ± 0.010	—

Aisa denotes AisaFENIX; pp denotes percentage points. Source and target references were evaluated on the corresponding held-out seeds; shrinkage LDA used all 101 coordinates with λ = 0.1. All transfer columns use PCA–LDA. Ridge DS uses 70 calibration seed pairs per accession and a ridge penalty of 10^−4^; the per-band location/scale map was evaluated for reflectance only. PDS maps target to source and reuses the source classifier; CORAL aligns source to target and refits the classifier. Both spectra of each test seed were excluded from fitting and adaptation. SDs describe overlapping partitions, and numerical rankings compare complete pipelines rather than isolated alignment operators.

**Table 3:** Fixed-split paired-bootstrap gains for PDS and CORAL versus direct transfer. Macro-F1 differences and stratified paired-bootstrap 95% confidence intervals across 12 specified contrasts. Exact raw and Holm-adjusted *p* values are retained in Table S8.

Representation	Direction	Method	Gain (pp)	95% CI (pp)
Reflectance	AisaFENIX → Headwall	PDS	20.27	17.14–23.51
Reflectance	AisaFENIX → Headwall	CORAL	21.73	18.63–24.96
Reflectance	Headwall → AisaFENIX	PDS	15.94	12.64–19.22
Reflectance	Headwall → AisaFENIX	CORAL	29.67	26.60–32.93
SNV	AisaFENIX → Headwall	PDS	6.29	3.87–8.68
SNV	AisaFENIX → Headwall	CORAL	9.30	6.77–12.08
SNV	Headwall → AisaFENIX	PDS	19.07	16.62–21.74
SNV	Headwall → AisaFENIX	CORAL	12.84	10.38–15.48
First derivative	AisaFENIX → Headwall	PDS	18.38	15.14–21.76
First derivative	AisaFENIX → Headwall	CORAL	20.26	16.98–23.66
First derivative	Headwall → AisaFENIX	PDS	22.06	19.47–24.97
First derivative	Headwall → AisaFENIX	CORAL	20.55	17.94–23.48

Inference is conditional on one fixed seed-pair test partition and calibration set, using 4999 stratified paired-bootstrap replicates. Intervals do not include variation among calibration sets, scanning sessions, lots, days or instrument units; pp denotes percentage points.

## Data Availability

The original contributions presented in this study are included in the article and Supplementary Materials; further inquiries can be directed to the corresponding author.

## Supplementary Materials

The following supporting information can be downloaded at: https://www.mdpi.com/article/10.3390/s26216931/s1. Supplementary Methods S1.1–S1.17, Supplementary Results S2.1–S2.2, Figures S1–S10 and Tables S1–S26 provide definitions, supporting results, calibration and classifier comparisons, and sensitivity analyses.
