# Supplementary material for: Conserved Accession–Lot Geometry but Limited Individual Spectral Retrieval: Bidirectional Transfer Across Two Hyperspectral Imaging Systems
Source: Sensors (Basel). 2026 Nov 2;26(21):6931. doi: 10.3390/s26216931 (PMC13766424; doi:10.3390/s26216931)
Supplement: Supplementary file 1 [file sensors-26-06931-s001.zip › sensors-4568849-supplementary.pdf]

sensors

MDPI

Supplementary Materials

Conserved Accession–Lot Geometry but Limited Individual Spectral Retrieval: Bidirectional Transfer across Two Hyperspectral Imaging Systems

Renan Falcioni [1,*], Roney Berti de Oliveira [1], Marcelo Luiz Chicati [1], José A. M. Demattê [2], and Marcos Rafael Nanni [1]

1 Graduate Program in Agronomy, State University of Maringá, Av. Colombo 5790, Maringá 87020-900, PR, Brazil; rboliveira@uem.br (R.B.d.O.); mlchicati@uem.br (M.L.C.); mrnanni@uem.br (M.R.N.)
2 Department of Soil Science, Luiz de Queiroz College of Agriculture, University of São Paulo, Av. Pádua Dias, 11, Piracicaba 13418-260, SP, Brazil; jamdemat@usp.br (J.A.M.D.)
* Correspondence: renanfalcioni@gmail.com or rfalcioni2@uem.br; Tel.: +55-44-3011-1359

The Supplementary Materials comprise Sections S1.1–S1.17 and S2.1–S2.2, Figures S1–S10 and Tables S1–S26. They provide mathematical definitions, supporting numerical results and sensitivity analyses. Reference numbers correspond to the bibliography in the main article.

Supplementary Method S1. Mathematical Definitions and Implementation Details

The equations below define the analyses. Symbols and numerical conventions are introduced within each subsection; Table S10 summarises the primary analysis parameters, and Sections S1.7–S1.17 describe the additional analyses in Tables S11–S26.

S1.1. Native-Axis Smoothing, Interpolation, SNV, and Differentiation

For spectrum i, a local third-order polynomial was fitted at wavelength coordinate j over a reflected window with half-width h. The fitted intercept defined the smoothed value, and the fitted first-order coefficient defined the derivative. Linear interpolation was applied only after native-axis smoothing, and standard normal variate (SNV) transformation was applied independently to each spectrum.

$$\widehat{\boldsymbol{\beta}}_{ij} = \arg\min_{\boldsymbol{\beta}} \sum_{q=-h}^{h} \left[x_i(\lambda_{j+q}) - \sum_{\ell=0}^{3} \beta_\ell (q\Delta\lambda)^\ell \right]^2$$

$$\tilde{x}_i(\lambda_j) = \widehat{\beta}_0, x'_i(\lambda_j) = \widehat{\beta}_1$$

$$x_i(\lambda^*) = x_i(\lambda_l) + \frac{\lambda^* - \lambda_l}{\lambda_u - \lambda_l}\left(x_i(\lambda_u) - x_i(\lambda_l)\right)$$

$$z_{ij} = \frac{x_{ij} - \bar{x}_i}{s_i}$$

$$\bar{x}_i = \frac{1}{P}\sum_{j=1}^{P} x_{ij}, s_i = \sqrt{\frac{1}{P-1}\sum_{j=1}^{P}\left(x_{ij} - \bar{x}_i\right)^2}$$

For native-axis smoothing, h = 4 for AisaFENIX (9 points), and h = 10 for Headwall (21 points). The products of the window-point count and median wavelength increment were approximately 15.48 and 15.33 nm, respectively; first-to-last coordinate spans were 13.76 and 14.60 nm. Interpolation was evaluated at 450, 455, …, 950 nm. For the first derivative, h = 3, the window contained 7 points, and the wavelength increment was 5 nm.

In the SNV expressions, P = 101. Reflectance and SNV were dimensionless; derivatives were expressed as reflectance per nm.

S1.2. Accession Geometry and Individual Spectral Retrieval

Accession centroids, wavelength-standardised centroid coordinates, pairwise Euclidean distances, the consensus rank-distance vector, Mantel association, symmetric Procrustes agreement, and the RV coefficient were defined as follows:

$$\mu_{aj}^{(d)(r)} = \frac{1}{n_a} \sum_{i:y_i=a} x_{ij}^{(d)(r)}$$

$$z_{aj}^{(d)(r)} = \frac{\mu_{aj}^{(d)(r)} - \bar{\mu}_{\cdot j}^{(d)(r)}}{s_{\mu,j}^{(d)(r)}}$$

$$d_{ab}^{(d)(r)} = \sqrt{\sum_j \left(z_{aj}^{(d)(r)} - z_{bj}^{(d)(r)}\right)^2}$$

$$M = 496, d_{ab,\mathrm{cons}}^{(d)} = \frac{1}{3} \sum_{r=1}^{3} \frac{\mathrm{rank}\left(d_{ab}^{(d)(r)}\right)}{M}$$

$$\rho_{\mathrm{M}} = \mathrm{cor}_{\mathrm{Spearman}}\left(\left\{d_{ab}^{(\mathrm{A})}\right\}, \left\{d_{ab}^{(\mathrm{H})}\right\}; a < b\right)$$

$$\mathrm{ss} = \min_{\mathbf{Q}^{\mathrm{T}}\mathbf{Q}=\mathbf{I}} \|\mathbf{X} - \mathbf{YQ}\|^2, r_{\mathrm{P}} = \sqrt{1 - \mathrm{ss}}$$

$$\mathbf{G}_{\mathbf{X}} = \mathbf{XX}^{\mathrm{T}}, \mathbf{G}_{\mathbf{Y}} = \mathbf{YY}^{\mathrm{T}}, \mathrm{RV} = \frac{\langle \mathbf{G}_{\mathbf{X}}, \mathbf{G}_{\mathbf{Y}} \rangle_{\mathrm{F}}}{\|\mathbf{G}_{\mathbf{X}}\|_F \|\mathbf{G}_{\mathbf{Y}}\|_F}$$

Each accession contained n_a = 100 distinct physical seeds, and the 32 accession centroids yielded M = 496 pairwise distances. One seed lot was observed per accession. Tied distance ranks were averaged. Before symmetric Procrustes analysis, each distance matrix was converted to k = 10 classical multidimensional-scaling coordinates with cmdscale(add = TRUE). In the Procrustes expression, X and Y are centred and symmetrically scaled MDS configurations; in the RV expression, they are wavelength-standardised accession-centroid matrices, and G_X and G_Y are the corresponding Gram matrices. Significance for the Mantel and Procrustes analyses was assessed using 999 permutations. The consensus distance vector combines the same three spectral representations and is descriptive rather than independent validation. Figure 2A in the main article displays the first two aligned coordinates for all 32 accession–lot centroid pairs; the Procrustes statistic uses all ten coordinates. Each centroid averages 100 seed spectra, so these links describe accession–lot geometry rather than individual-seed retrieval.

For individual-seed analyses, spectra were used as complete profiles or residualised by subtracting the accession-specific mean within each system and representation. After row standardisation, Pearson correlation was equivalent to cosine similarity. For each source profile, the 100 target profiles from the same accession were ranked by correlation; top-1 retrieval was the proportion for which the target measurement with the highest correlation came from the same seed. This exploratory profile-ranking analysis was separate from the fitted accession-classification and calibration-transfer models.

$$e_{ij}^{(d)(r)} = x_{ij}^{(d)(r)} - \mu_{a(i)j}^{(d)(r)}$$

$$z_{ij}^{(d)(r)} = \frac{e_{ij}^{(d)(r)} - \bar{e}_i^{(d)(r)}}{s_{e,i}^{(d)(r)}}$$

$$S_{ik} = \frac{1}{P-1} \sum_{j=1}^{P} z_{ij}^{(\mathrm{A})} z_{kj}^{(\mathrm{H})}$$

$$\hat{k}_i = \underset{k:a(k)=a(i)}{\arg\max} S_{ik}, \mathrm{Top1} = \frac{1}{N} \sum_{i=1}^{N} \mathbb{I}[\hat{k}_i = \pi(i)]$$

$$T_{\text{obs}} = \frac{1}{N}\sum_{i=1}^{N} S_{i\pi(i)}, p = \frac{1+\sum_{b=1}^{999}\mathbb{I}[T_b \geq T_{\text{obs}}]}{1000}$$

The same-seed pairing map is denoted by π. The same 100 distinct physical seeds per accession remained in fixed positions and retained the same face and orientation during same-day imaging with both systems. Pairs were defined by accession and fixed physical position. Ties were resolved by choosing the first index, and uniform random selection among 100 candidates gave a top-1 reference of 1/100. The mean same-seed similarity was assessed using an upper-tail permutation test with 999 independent within-accession permutations.

S1.3. PCA–LDA, Performance Metrics, and Distribution-Shift Summaries

Classification performance was evaluated on held-out physical seeds: both sensor measurements of each test seed were excluded from model fitting and transfer calibration. Source-development features were centred and standardised before principal component analysis. The smallest number of components explaining at least 99.5% of the variance was retained subject to the implemented rank limits, after which linear discriminant analysis was fitted to the retained scores.

$$z_{ij} = \frac{x_{ij} - \bar{x}_j}{s_j}, \mathbf{t}_i = \mathbf{z}_i \mathbf{V}_K$$

$$K_0 = \min_{\left\{k: \frac{\sum_{\ell=1}^{k}\lambda_\ell}{\sum_\ell \lambda_\ell} \geq 0.995\right\}}, K_{\max} = \min(60, \mathrm{n} - \mathrm{C}, \mathrm{p})$$

$$K = \max\big(2, \min(K_0, K_{\max})\big)$$

$$\delta_c = \mathbf{t}^{\mathrm{T}}\boldsymbol{\Sigma}^{-1}\boldsymbol{\mu}_c - \frac{1}{2}\boldsymbol{\mu}_c^{\mathrm{T}}\boldsymbol{\Sigma}^{-1}\boldsymbol{\mu}_c + \log(\pi_c), \hat{y} = \arg\max_c \delta_c$$

With n training seeds, p features, and C = 32 classes, K_max = min(60, n − C, p), with at least two retained components. Balanced development partitions gave class priors π_c = 1/32. Training-feature standard deviations were replaced by 1 before standardisation if they were non-finite or below 10^{-10}.

Classwise precision, recall, F1, macro-F1, accuracy, balanced accuracy, and the mean- and covariance-gap diagnostics were defined as follows:

$$P_c = \frac{\mathrm{TP}_c}{\mathrm{TP}_c + \mathrm{FP}_c}, R_c = \frac{\mathrm{TP}_c}{\mathrm{TP}_c + \mathrm{FN}_c}$$

$$F_{1,c} = \frac{2P_c R_c}{P_c + R_c}, F_{1,\text{macro}} = \frac{1}{32}\sum_{c=1}^{32} F_{1,c}$$

$$\text{Accuracy} = \frac{\sum_c \mathrm{TP}_c}{N}, \text{Balanced Accuracy} = \frac{1}{32}\sum_{c=1}^{32} R_c$$

$$g_\mu = \|\bar{\mathbf{z}}_{\mathrm{S}} - \bar{\mathbf{z}}_{\mathrm{T}}\|_2, g_\Sigma = \frac{\|\boldsymbol{\Sigma}_{\mathrm{S}} - \boldsymbol{\Sigma}_{\mathrm{T}}\|_F}{\max\left(\|\boldsymbol{\Sigma}_{\mathrm{T}}\|_F, 10^{-12}\right)}$$

If a class received no predictions, its precision was defined as zero; class F1 was zero whenever precision plus recall was zero. The mean-vector and normalised covariance gaps were calculated in a scenario-specific pooled z-space, and the Frobenius-norm denominator was bounded below by 10^{-12}.

S1.4. Pairing Control, Bootstrap Inference, Exploratory Pairing Band, and Grid Sensitivity

Target-calibration spectra were deranged separately within each accession. The permutation had no fixed points, so each source seed was paired with a different target seed from the same accession.

$$\pi_a: I_a \rightarrow I_a, \pi_a(i) \neq i, \mathbf{x}_{\mathrm{T},i}^{\text{shuffled}} = \mathbf{x}_{\mathrm{T},\pi_a(i)}$$

$$\Delta_{\text{pair}} = F_{1,\text{linked}} - F_{1,\text{shuffled}}$$

Paired-bootstrap contrasts used identical accession-stratified test-seed resamples for the compared methods. Percentile intervals, plus-one p values, and Holm adjustment were defined as follows. The fixed-split pairing comparison used an illustrative ±0.010 macro-F1 sensitivity band.

$$\Delta^{(b)} = F_{1,\mathrm{A}}^{(b)} - F_{1,\mathrm{B}}^{(b)}$$

$$\mathrm{CI}_{1-\alpha} = \left[Q_{\frac{\alpha}{2}}\left(\Delta^{(b)}\right), Q_{1-\frac{\alpha}{2}}\left(\Delta^{(b)}\right)\right]$$

$$p = \frac{1 + \sum_{b=1}^{B} \mathbb{I}\left[\left|\Delta^{(b)} - \Delta_{\mathrm{obs}}\right| \geq \left|\Delta_{\mathrm{obs}}\right|\right]}{B+1}$$

$$\tilde{p}_{(i)} = \min\left(1, \max_{j \leq i}\left\{(m-j+1)p_{(j)}\right\}\right)$$

$$\mathrm{CI}_{90\%}\left(\Delta_{\mathrm{pair}}\right) \subseteq [-0.010, 0.010]$$

The analysis used B = 4,999 replicates. Scenario intervals were 95% percentile intervals, and m = 12 adaptation-versus-direct-transfer contrasts were adjusted jointly. With the plus-one convention, the smallest attainable unadjusted bootstrap p value was 0.0002. The fixed-split same-seed-versus-deranged calibration comparison used 90% intervals against an illustrative ±0.010 macro-F1 band. The 90% level is consistent with the interval convention for two one-sided 5% comparisons against a band; here, the band was exploratory. This separate interval display addresses the magnitude of the calibration-pair effect; it does not use the 95% intervals or the multiplicity-adjusted tests for adaptation gains. It is conditional on one split and one within-accession derangement and does not establish biological equivalence. The split, bootstrap, and derangement seeds were 20,960,726, 20,960,727, and 20,960,728, respectively.

For the numerical-support sensitivity analysis, changes were expressed relative to the 450–950 nm grid at 5 nm spacing.

$$\Delta \mathrm{F}_{1,g,r}^{\mathrm{pp}} = 100\left(F_{1,g,r} - F_{1,450-950/5\,\mathrm{nm},r}\right)$$

PDS used h = 2 on the 5 nm grids and h = 1 on the 10 nm grids. The 450–950 nm interval provided conservative shared numerical support, avoiding the Headwall range edges and the AisaFENIX VNIR/SWIR transition near 970 nm, and 5 nm was the chosen numerical sampling increment. This comparison changed numerical support and sampling density only and did not model measured sensor response functions or optical FWHM.

S1.5. Compact-Panel Components and Seed-Level Concordance Diagnostics

The source- and target-domain accession-discrimination terms were based on one-way η^2 at each wavelength. The sums of squares and bounded η^2 were defined as follows.

$$\mathrm{SSB}_{d,j} = \sum_{a=1}^{32} n_a\left(\bar{x}_{d,a,j} - \bar{x}_{d,\cdot,j}\right)^2, \mathrm{SST}_{d,j} = \sum_{i}\left(x_{d,i,j} - \bar{x}_{d,\cdot,j}\right)^2$$

$$\eta_{d,j}^2 = \min\left(1, \max\left(0, \frac{\mathrm{SSB}_{d,j}}{\max\left(\mathrm{SST}_{d,j}, 10^{-12}\right)}\right)\right)$$

The composite panel combined the geometric mean of source and target η^2, an exponential domain-stability term, and non-negative accession-centroid correlation. Each component was bounded before calculating the harmonic mean. The exact definitions of D_j, Δ_j, U_j, C_j, H_j, and the regional maximiser are given in Section 2.10 of the main manuscript. Within-accession residual Pearson correlation and Lin's concordance correlation coefficient were retained only as diagnostics. Target-domain accession labels contribute to η^2 and the panel score; the panel is selected with this label information and evaluated within the study.

$$r_j = \frac{\mathrm{cov}\left(e_{\mathrm{S},j}, e_{\mathrm{T},j}\right)}{s_{\mathrm{S},j} s_{\mathrm{T},j}}$$

$$\rho_{c,j} = \frac{2\mathrm{cov}(e_{\mathrm{S},j}, e_{\mathrm{T},j})}{s_{\mathrm{S},j}{}^2 + s_{\mathrm{T},j}{}^2 + (\bar{e}_{\mathrm{S},j} - \bar{e}_{\mathrm{T},j})^2 + 10^{-12}}$$

In these expressions, e_S and e_T are within-accession residuals for the same-seed spectra. The denominator in Lin's coefficient was bounded below by 10^{-12}. Neither residual correlation nor concordance entered the composite panel score.

S1.6. Probability Calibration, Prediction Sets, and Selective Classification

For posterior probability p_{ic} and true class y_i, log loss, multiclass Brier score, expected calibration error, split-conformal nonconformity, the finite-sample threshold, and standard prediction-set summaries were defined as follows:

$$\mathrm{LogLoss} = -\frac{1}{N}\sum_{i=1}^{N} \log\left(\max\left(p_{iy_i}, 10^{-15}\right)\right)$$

$$\mathrm{Brier} = \frac{1}{N}\sum_{i=1}^{N}\sum_{c=1}^{32} (p_{ic} - \mathbb{I}[y_i = c])^2$$

$$\mathrm{ECE}_{10} = \sum_{b=1}^{10} \frac{|B_b|}{N} |\mathrm{acc}(B_b) - \mathrm{conf}(B_b)|$$

$$a_i = 1 - p_{iy_i}$$

$$k = \min(n_{\mathrm{cal}}, \lceil (n_{\mathrm{cal}} + 1)(1 - \alpha) \rceil), \hat{q}_\alpha = a_{(k)}$$

$$\Gamma_\alpha = \{c{:}\, 1 - p_c \le \hat{q}_\alpha\}$$

$$\mathrm{Coverage} = \frac{1}{N}\sum_{i=1}^{N} \mathbb{I}[y_i \in \Gamma_i]\,, \mathrm{Mean\ set\ size} = \frac{1}{N}\sum_{i=1}^{N} |\Gamma_i|\,, \mathrm{Singleton\ rate} = \frac{1}{N}\sum_{i=1}^{N} \mathbb{I}[|\Gamma_i| = 1]$$

Log loss used a lower probability bound of 10^{-15}, and ECE used 10 equal-width confidence bins. Source-domain conformal calibration had n_{cal} = 640, α = 0.10, and order-statistic index k = 577. Singleton accuracy, empty-set rate, and accession-specific coverage were calculated from the same sets.

For the exploratory importance-weighted analysis, a cross-validated domain classifier supplied target-domain probabilities and density-ratio weights. Effective sample size, weighted classwise p values, weighted prediction sets, and risk–coverage summaries were defined as follows:

$$\tilde{\pi}_{\mathrm{T},i} = \min\left(1 - 10^{-5}, \max\left(10^{-5}, \hat{\pi}_{\mathrm{T},i}\right)\right)$$

$$w_{\mathrm{raw},i} = \frac{\tilde{\pi}_{\mathrm{T},i}}{1 - \tilde{\pi}_{\mathrm{T},i}} \frac{\pi_\mathrm{S}}{\pi_\mathrm{T}}, w_i = \min\left(20, \max(0.05, w_{\mathrm{raw},i})\right)$$

$$\mathrm{ESS} = \frac{(\sum_i w_i)^2}{\sum_i w_i{}^2}$$

$$p_{ic}^{(w)} = \frac{w_i + \sum_{r\in\mathrm{cal}} w_r \mathbb{I}[a_r \ge 1 - p_{ic}]}{w_i + \sum_{r\in\mathrm{cal}} w_r}$$

$$\Gamma_i^{(w)} = \left\{c{:}\, p_{ic}^{(w)} > \alpha\right\}$$

$$q_i = \max_c p_{ic}, \mathrm{Risk}(\tau) = 1 - \frac{\sum_{i\in R_\tau} \mathbb{I}[\hat{y}_i = y_i]}{|R_\tau|}$$

The domain model used 20 principal components and five cross-validation folds. Estimated target probabilities were clipped to [10^{-5}, 1 − 10^{-5}], and weights were truncated to 0.05–20 after source-to-target prior correction. For a retained fraction τ, R_τ contained the floor of τN predictions with the highest maximum posterior probabilities.

S1.7. Experimental Unit and Paired Acquisition

The paired analysis comprised spectra from 32 accessions with 100 distinct physical seeds per accession. The same seeds in fixed Petri-dish supports retained their positions, exposed faces and orientations during same-day imaging with both systems; one

spectrum per seed and sensor was analysed. Pairs were defined by accession and fixed physical position (1–100). Headwall spectra were obtained by averaging 100 pixels in a central 10 × 10-pixel ROI [1]. AisaFENIX spectra were obtained by averaging ten selected interior pixels within a manually placed central sampling window (10 × 10 pixels where feasible) [2]. These ROIs sampled corresponding central interior seed-coat regions, with pixel selection adjusted to each camera's imaging geometry and spatial resolution.

Four Headwall sessions and three AisaFENIX sessions were used for equipment calibration and illumination adjustments. The analysed spectra were acquired in groups of 100 distinct seeds in fixed Petri-dish supports, with one conveyor pass per group under each camera. The support arrangement, seed positions, exposed faces and orientations were maintained between the two same-day measurements, and conveyor speed was held constant. Camera-specific acquisition settings are described in Section 2.2 of the main article. Validation partitions were formed at the physical-seed level, keeping both measurements of each seed together.

S1.8. Split-Half and PC1-Excluded Accession Geometry

In each of 30 repeated partitions, the 100 seeds within every accession were divided into two disjoint sets of 50, using the same membership in both sensor matrices. We computed accession centroids for each half and representation and then compared Euclidean accession-distance matrices using Spearman correlation of their upper-triangular entries, reported descriptively as Mantel r. Symmetric Procrustes correlation, and the RV coefficient provided complementary geometry summaries. Comparisons comprised AisaFENIX half 1 versus half 2, Headwall half 1 versus half 2, and matched cross-sensor halves. These split halves assess the stability of accession centroids across different seeds; they do not measure repeatability of a physical seed scanned twice by one sensor.

In a separate sensitivity analysis, the 32 accession centroids from each sensor were stacked and wavelength standardised jointly. We computed one common principal-component basis, removed its first component, and recalculated the cross-sensor geometry. The all-component reference in this calculation uses joint scaling and should be compared with its own PC1-excluded value rather than treated as numerically identical to the geometry reported in Table S2C.

S1.9. Repeated Derangements and Adaptation Parameter Sensitivity

For each of the 30 pair-preserving 70:30 development/test partitions, we generated five independent fixed-point-free permutations of target calibration spectra within every accession. The source development data, accession composition, number of target calibration spectra, PDS settings, and held-out target test set were unchanged; only exact source-to-target calibration-pair identity was broken. Across three representations and two directions, this produced 900 derangement evaluations (150 per representation-direction cell). Exact-minus-deranged macro-F1 was summarised across splits without a repeated-split significance test because the test sets overlap.

Parameter sensitivity used ten specified split IDs (1, 4, 7, 11, 14, 17, 21, 24, 27, and 30). Around the primary setting, one factor was varied at a time: PDS half-window h in {1, 2, 4}; PDS ridge in {10^{-8}, 10^{-6}, 10^{-4}}; CORAL covariance ridge in {10^{-6}, 10^{-4}, 10^{-2}}; and PCA cumulative-variance target in {0.990, 0.995, 0.999}. The held-out test labels were not used to fit transformations or select a setting; sensitivity results are descriptive and do not establish hyperparameter optimality.

S1.10. Additional Transfer Comparators and Calibration Budgets

We evaluated two simple reflectance alignment baselines at the full target-calibration budget: matching the target location and scale to those of the source calibration spectra at each band and a single gain-and-offset map estimated from the two calibration mean spectra. We also fitted a paired ridge direct-standardisation (DS) map from target to source

after domain-specific calibration standardisation. Its primary ridge was 10^{-4}, with 10^{-6} and 10^{-2} examined as sensitivity values. This implementation was motivated by the DS family discussed in Refs. [15,26]. DS used all 70 calibration pairs per accession (2,240 pairs total), so its results provide a paired benchmark with a high calibration budget.

Nested target-calibration budgets were 1, 2, 5, 10, 20, 40, and 70 seeds per accession. Alongside PDS and CORAL, we evaluated target-only PCA–LDA and PCA–LDA fitted to pooled source and labelled-target calibration spectra at matching target-label budgets for both reflectance and SNV. Test seeds remained held out. Target-only PCA–LDA with one labelled seed per accession was not estimable because 32 observations for 32 classes leave zero residual degrees of freedom; the corresponding result is missing by design, not zero. CORAL's alignment objective does not use class labels, but the budget subsets were selected using known accession membership.

S1.11. Sensitivity Restricted to Phaseolus Accessions

We excluded A27 (Vigna) and A28 (Cajanus) and repeated the within-sensor reference, direct transfer, PDS, and CORAL analyses for the remaining 30 Phaseolus accessions, all three representations, both transfer directions, and all 30 partitions. Accession geometry was recalculated on the same 30-accession subset. The analysis assesses dependence of the reported patterns on these two non-Phaseolus accessions.

S1.12. Regional Geometry and Paired Seed Bootstrap

For reflectance, SNV and first derivatives, accession geometry was recomputed over the 450–950, 450–700 and 700–950 nm intervals for all 32 accessions and after excluding A27/A28. The two subranges share 700 nm and are not independent. After selecting wavelengths and accessions, each sensor's centroid matrix was separately centred and scaled by wavelength; coordinates with a standard deviation of at most 10^{-12} were omitted. Spearman correlation compared Euclidean distance vectors, symmetric Procrustes analysis used ten classical multidimensional-scaling coordinates, and the RV coefficient compared centred Gram matrices, as in S1.2.

For the full interval and all 32 accessions, 999 bootstrap draws sampled 100 seed identifiers with replacement within each accession (random seed 20260928). Identical sampled identifiers were applied to both sensors and all representations. Centroids, scaling and geometry were recomputed in every draw. The 2.5th and 97.5th percentiles (R quantile type 8) define paired seed-bootstrap intervals conditional on these accession–lots and acquisitions; they exclude variation among new lots, days or instrument units.

S1.13. Shrinkage LDA, Cross-Accession Negative Control and Repeated Gains

Unless otherwise specified, additional transfer analyses used the same 30 accession-stratified 70:30 partitions, with seed 20260726 + r for repeat r = 1,…,30. Both sensor measurements of each physical seed were assigned to the same partition: 2,240 development and 960 held-out test pairs. Ten-split sensitivities used r = 1, 4, 7, 11, 14, 17, 21, 24, 27 and 30.

Within-sensor shrinkage LDA used all 101 coordinates without PCA. Features were standardised using development-set means and standard deviations; standard deviations below 10^{-10} were replaced by one. Class means were estimated from the standardised development data. The pooled within-class covariance S was estimated from the within-class development residuals by dividing their cross-product by 2,240 − 32. Covariance was replaced by $(1 - \lambda)S + \lambda\ \mathrm{tr}(S)I/101$. For each class, the score was the test vector's inner product with the inverse-covariance-weighted class mean minus half the mean's squared Mahalanobis length. Equal class priors were used; the largest score determined the prediction. The reference value $\lambda = 0.1$ and sensitivity values 0.01/0.5 were fixed and were not selected using test data; both sensors and all three representations were evaluated on all 30 splits.

For each split, five cross-accession controls used seed 20260928 + 1000 + 100r + d, with draw d = 1,…,5. A fixed-point-free permutation assigned each accession to another accession; random within-destination ordering produced a bijection of all development seeds. PDS was refitted with these deliberately incorrect calibration matches (half-window two; ridge 10^{-6}); source classifiers and test sets were unchanged. The five results were averaged within each split before comparison with exact-pair PDS. This tests accession correspondence separately from the within-accession control in S1.9. PDS-minus-direct and CORAL-minus-direct gains were also paired within each development/test split. Across-split ranges are descriptive summaries, not inferential estimates based on independent replicates.

S1.14. Target-Panel Composition, Covariance Sensitivity and Held-Out Domain Discrimination

Reflectance CORAL used the ten specified splits, five calibration draws per split, and target calibration budgets of 32/64/160. All 2,240 source development spectra remained available. Balanced panels contained 1/2/5 seeds per accession; unbalanced panels were simple random samples without replacement from the target development pool. The samples at successive budgets were nested prefixes of a random ordering within each draw. Balanced ordering used seed 20260928 + 10000 + 100r + d; unbalanced ordering added 100000. The same identifiers were used in both directions. Balanced sampling used target accession labels; random sampling and covariance alignment did not. No test seed entered either panel.

Pooled development scaling preceded CORAL (ridge $\varepsilon = 10^{-4}$), which was followed by PCA–LDA with 99.5% variance retention, subject to the limits in S1.3. For n target calibration spectra and p = 101 coordinates, covariance rank was defined as the number of eigenvalues exceeding max(n,p) times the largest eigenvalue times machine precision. The unregularised condition number was infinite for rank-deficient matrices; the regularised condition number was (largest eigenvalue + ε)/max(smallest eigenvalue + ε, ε). For the first draw at the balanced budget of 32, separate sensitivity analyses changed ε to $10^{-6}/10^{-2}$ or PCA retention to 99.0%/99.9%, one factor at a time. No optimum was selected. For performance summaries, results were averaged over the five calibration draws within each outer split before the mean and SD were calculated across the ten splits. Composition and covariance diagnostics describe all 50 split/draw evaluations per cell; parameter sensitivities describe ten splits.

A separate domain diagnostic used all 30 primary 70:30 splits and both directions. Development-fitted PDS mapped target observations to source coordinates; CORAL mapped source observations to target coordinates, including held-out source spectra. Unadapted spectra provided the reference. A ridge logistic classifier distinguished sensors using 20 PCs after scaling and PCA were fitted to the 4,480 transformed development observations. Five-fold development CV minimised binomial deviance; paired measurements shared folds, with seed 20260928 + 500000 + r. Transformations and PCA were not refitted inside these tuning folds. Final AUC was calculated from 1,920 observations from the 960 completely held-out seed pairs, with target probability as the positive score, average ranks for ties, and no post hoc reversal. Score variability was checked: constant post-alignment probabilities yield AUC 0.5 but do not demonstrate the absence of covariance, nonlinear or class-conditional shift. Internal-CV AUC is distinct from this outer-test diagnostic.

S1.15. Development-Only mRMR Wavelength Selection

A minimum-redundancy maximum-relevance comparator used the mutual-information difference criterion [40]. Reflectance panels used the same compact-panel partitions, seed 20260726 + 70000 + r, distinct from the primary transfer partitions. The 2,240

development spectra per sensor were pooled with their accession labels (4,480 rows). Reflectance at each wavelength was discretised using its development-pool empirical deciles (R quantile type 8); repeated boundaries were collapsed. Empirical mutual information was the sum of positive joint-cell probabilities multiplied by the natural logarithm of the ratio of the joint probability to the product of the marginal probabilities.

The first band maximised information about accession identity. Each subsequent band maximised that relevance minus mean pairwise mutual information with already selected bands. Ties were resolved by wavelength order. The deterministic greedy ranking supplied 16 bands in all 30 splits and 8/32 bands in the ten specified splits. Selected columns were reordered by wavelength before refitting direct-transfer, PDS and CORAL pipelines. PDS's two-neighbour half-window follows selected-column adjacency, not a fixed physical span. Both directions used the same pooled ranking. Target-development labels matched the supervision available to the transportability score; test spectra and labels entered neither binning nor selection. Panel sizes were sensitivity budgets, not optimised designs.

S1.16. Native-Smoothing Sensitivity and Descriptive Uncertainty Ranges

Requested native Savitzky–Golay spans were 5/15/25 nm. Window size was obtained by dividing the requested span by the median native wavelength increment and rounding to the nearest integer. The window contained at least five points and was increased by one if its size was even. Third-order smoothing used reflected edge padding. AisaFENIX windows contained 5/9/15 points and Headwall windows 7/21/35; first-to-last coordinate spans were respectively 6.88/13.76/24.08 and 4.38/14.60/24.82 nm. Linear interpolation then produced the common 101-point grid, followed by SNV or the fixed seven-point first-derivative filter with a third-order polynomial at 5 nm spacing. Every transfer model was refitted for each span, representation and direction on the ten specified splits. This assesses processing sensitivity, not harmonisation of measured optical responses or FWHM.

Uncertainty summaries used the 30 separate 50/20/30 partitions for reflectance (seed 20260726 + 110000 + r), reserving 50 seeds per accession for fitting/paired adaptation, 20 source seeds for conformal calibration, and 30 for testing. Density-ratio tuning used random seed 20260726 + r + c, with c = 150000, 160000 and 170000 for direct transfer, PDS and CORAL, respectively; other definitions are given in S1.6. ECE, accuracy, macro-F1, standard/weighted target coverage and selective accuracy at 50% retention were summarised by the mean, sample SD and empirical 2.5th–97.5th percentiles (type 8). Weighted-minus-standard coverage was paired within each split, direction and scenario. These are descriptive central ranges across overlapping partitions, not confidence intervals or target-domain coverage guarantees.

S1.17. Mean Reflectance, PC1, and Order-Sensitivity Summaries

Additional exploratory summaries characterised within-accession cross-sensor associations in reflectance magnitude. Mean reflectance was calculated across the 101 common wavelengths from 450 to 950 nm, without SNV or row standardisation. A separate covariance PCA was fitted to the 3,200 spectra from each sensor after band centring, without variance scaling. The sign of PC1 was oriented towards each sensor's own mean reflectance. For each summary (mean reflectance and PC1 scores), cross-sensor Pearson correlations were calculated between the paired measurements within each accession; the aggregate statistic was the median correlation across the 32 accessions. Spearman correlations provided a rank-based sensitivity analysis.

Order sensitivity was examined by shifting the Headwall seed index by $k = -10, \ldots, +10$ relative to AisaFENIX, retaining only the $100 - |k|$ overlapping positions without circular wrapping, and by reversing the index as $i \mapsto 101 - i$. Zero offset retained the experimentally paired seeds; the other orders were perturbation controls for the correlation

summaries. Null distributions used 9,999 independent permutations of the complete Headwall vector within each accession, with the same permutations applied to both metrics and all 22 orders. Two-sided p values compared absolute correlations or absolute accession-median correlations with their permutation distributions, using the plus-one convention.

Holm adjustment covered the 64 zero-offset metric-by-accession comparisons. For the order sensitivities, a maximum-statistic adjustment jointly covered the 44 metric-by-order combinations, using the maximum absolute accession-median correlation from each permutation. A further Holm adjustment covered all 1,408 accession-by-metric-by-order comparisons. The permutation reference assumes exchangeability of seeds within each accession. These summaries use the same spectral observations and are supplementary association analyses, separate from the held-out classification results.

S2. Supplementary Results

S2.1. Numerical Support and Sampling Density

Numerical grid effects depended on direction and pipeline (Table S9; Figure S9). Changing 450–950 nm sampling from 5 to 10 nm had little effect on direct transfer and CORAL but reduced macro-F1 for reverse-direction PDS by 5.77 percentage points. Restricting support to 450–900 nm improved performance for some combinations and reduced it for others, including a 5.63-point reduction in reverse CORAL. These are numerical-support sensitivities; they do not measure changes in optical bandwidth. Native smoothing-span sensitivity is reported separately in Table S23.

Varying the nominal native smoothing span from 5 to 25 nm changed some transfer estimates appreciably (Table S23). For example, AisaFENIX-to-Headwall derivative PDS macro-F1 ranged from 0.500 to 0.584, while macro-F1 for reverse-direction direct transfer of reflectance ranged from 0.368 to 0.418. The 15 nm setting reproduced the reference matrices and predictions. These checks measure processing sensitivity and do not equate the cameras' effective optical resolutions.

S2.2. Compact Wavelength Panels

The transportability-oriented 16-band panel covered the spectrum consistently: a total of 14 wavelengths entered every development resample, with remaining variation near 500–510 and 700–705 nm (Figure S10A,B; Table S5). Its predictive effect depended on direction and method (Figure S10C). For AisaFENIX-to-Headwall CORAL, macro-F1 was 0.687, similar to 0.685 with 101 bands. For Headwall-to-AisaFENIX CORAL, it fell from 0.667 to 0.608. The uniformly spaced panel was more consistent, yielding 0.670 and 0.659 with CORAL in the two directions.

The 16 source-univariate-ranked bands performed poorly even within the source domain: source-reference macro-F1 was 0.431 and 0.513 in the two directions (Table S5). Their direct target macro-F1 was 0.139 and 0.147, and CORAL macro-F1 was 0.511 and 0.333. Therefore, their weak transfer cannot be attributed solely to a sensor mismatch. The label-informed transportability panel also reduced AisaFENIX-to-Headwall PDS from 0.673 to 0.594. Within-source discrimination, compactness, and cross-instrument portability require separate empirical checks.

The mRMR comparator matched the label budget of the transportability panel and selected bands using source- and target-development samples only. With 16 bands, direct/PDS/CORAL macro-F1 was 0.456/0.631/0.676 from AisaFENIX to Headwall and 0.376/0.588/0.647 in reverse (Table S24). Results with 8 and 32 bands varied by direction and adaptation pipeline. Thus, the custom transportability criterion is not uniformly superior to an established redundancy-aware selection rule.

Supplementary Figures

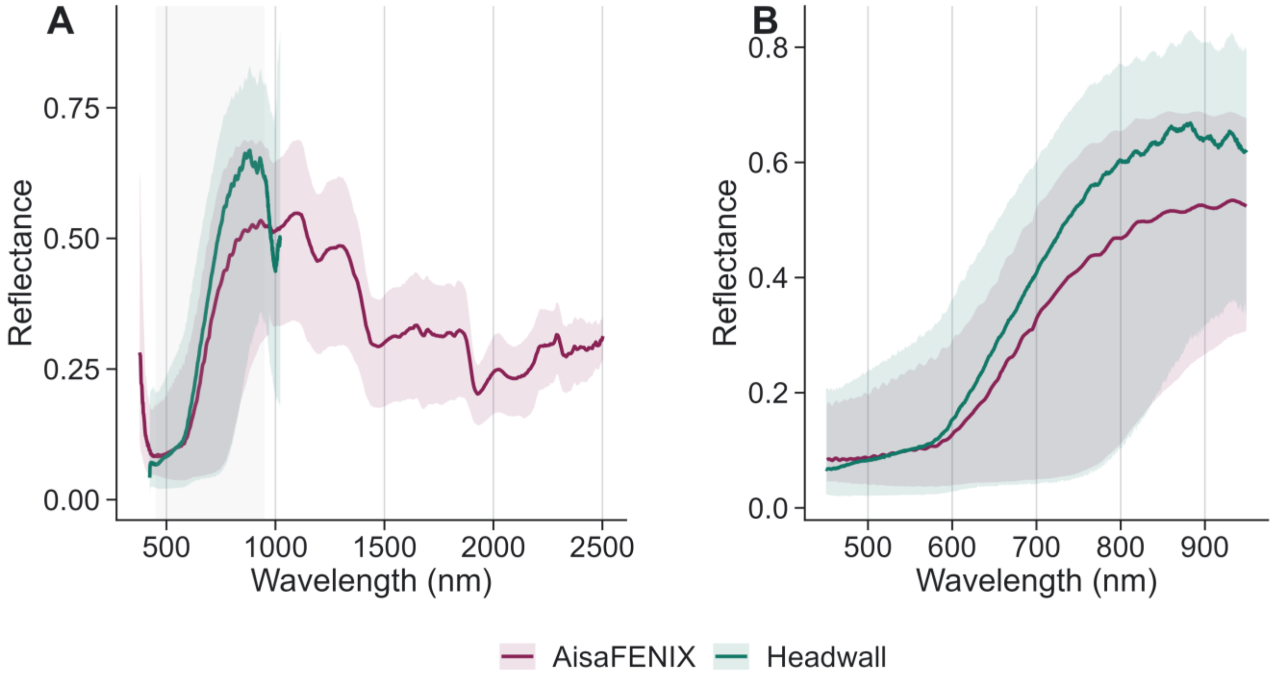

Figure S1. Spectral distributions of the source datasets. Median reflectance and the 5th–95th percentile envelope for 3,200 seeds per sensor. (A) Full native wavelength domains; the grey region marks the primary common interval. (B) Enlarged view of the 450–950 nm interval used for cross-sensor analysis.

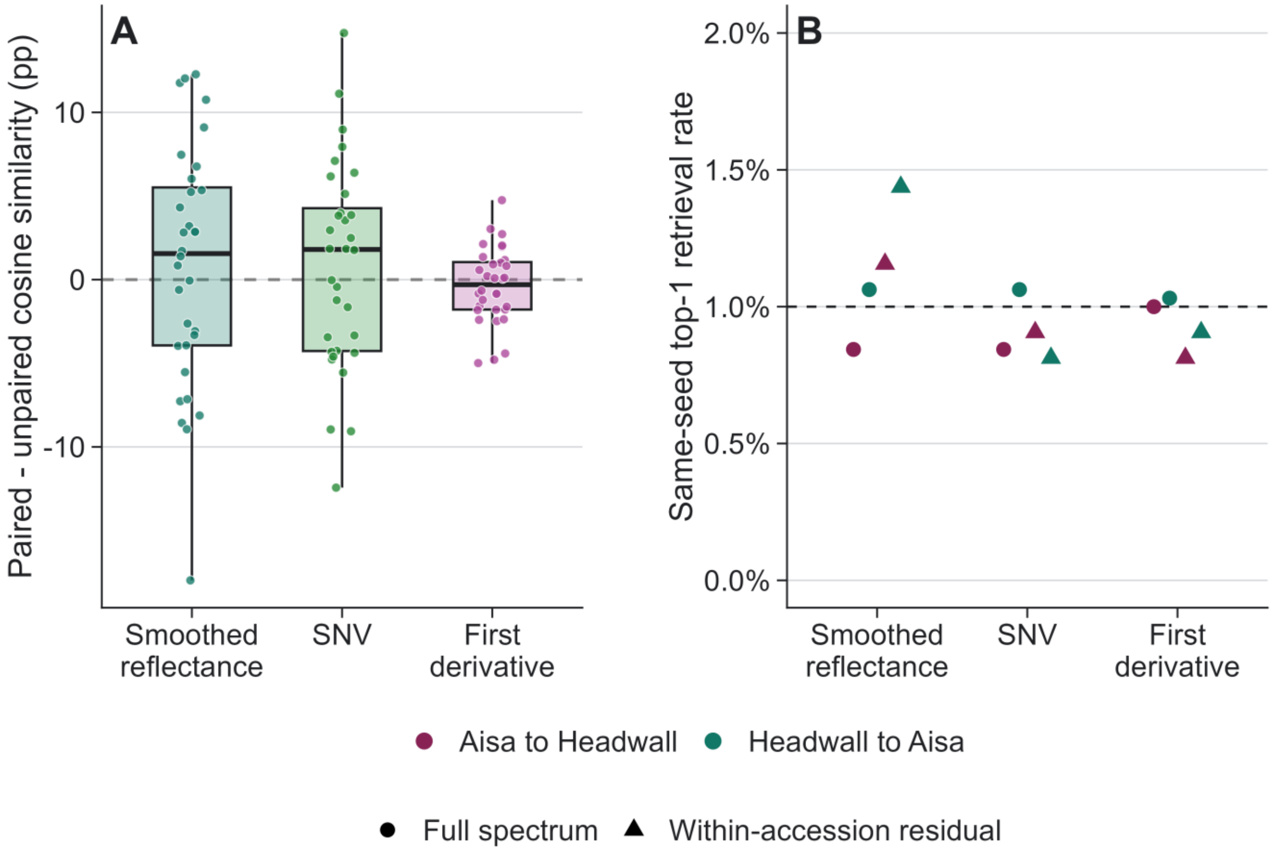

Figure S2. Exploratory cross-sensor retrieval of individual seed spectra. (A) Mean same-seed minus other-seed residual cosine similarity within each accession; each point represents one accession. (B) Same-seed top-1 retrieval for both directions and all three representations, using complete profiles or within-accession residuals; the dashed line is the 1% reference for uniform random selection among 100 candidates. Retrieval uses the largest profile correlation and is distinct from accession-classification accuracy.

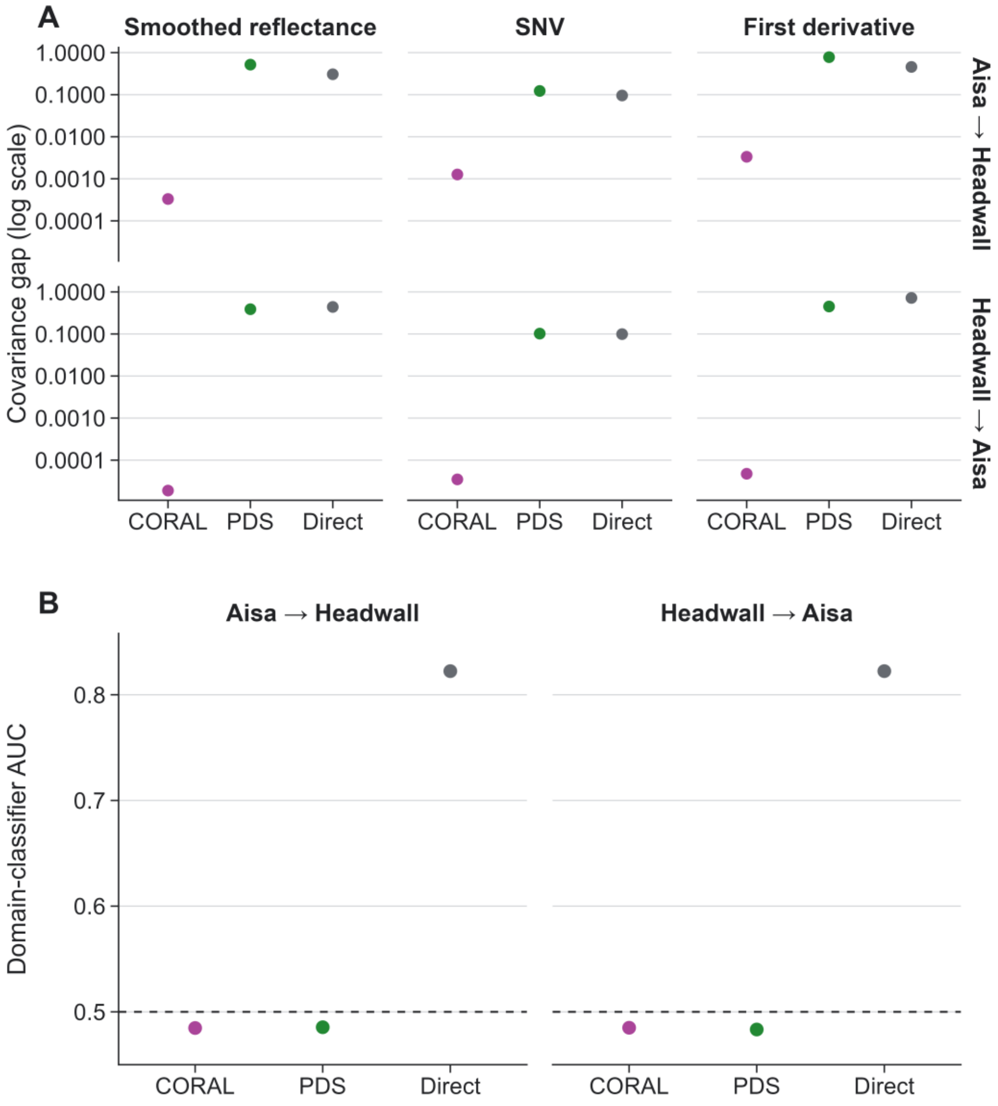

Figure S3. Domain-shift diagnostics before and after alignment. (A) Standardised covariance gaps for all three representations and both transfer directions. (B) Cross-validated linear domain-classifier AUC for smoothed reflectance; the dashed line marks chance discrimination (AUC = 0.5). Alignment and these diagnostics use calibration-domain data, so a near-zero gap or near-chance linear AUC is not independent evidence that all target-domain differences have disappeared.

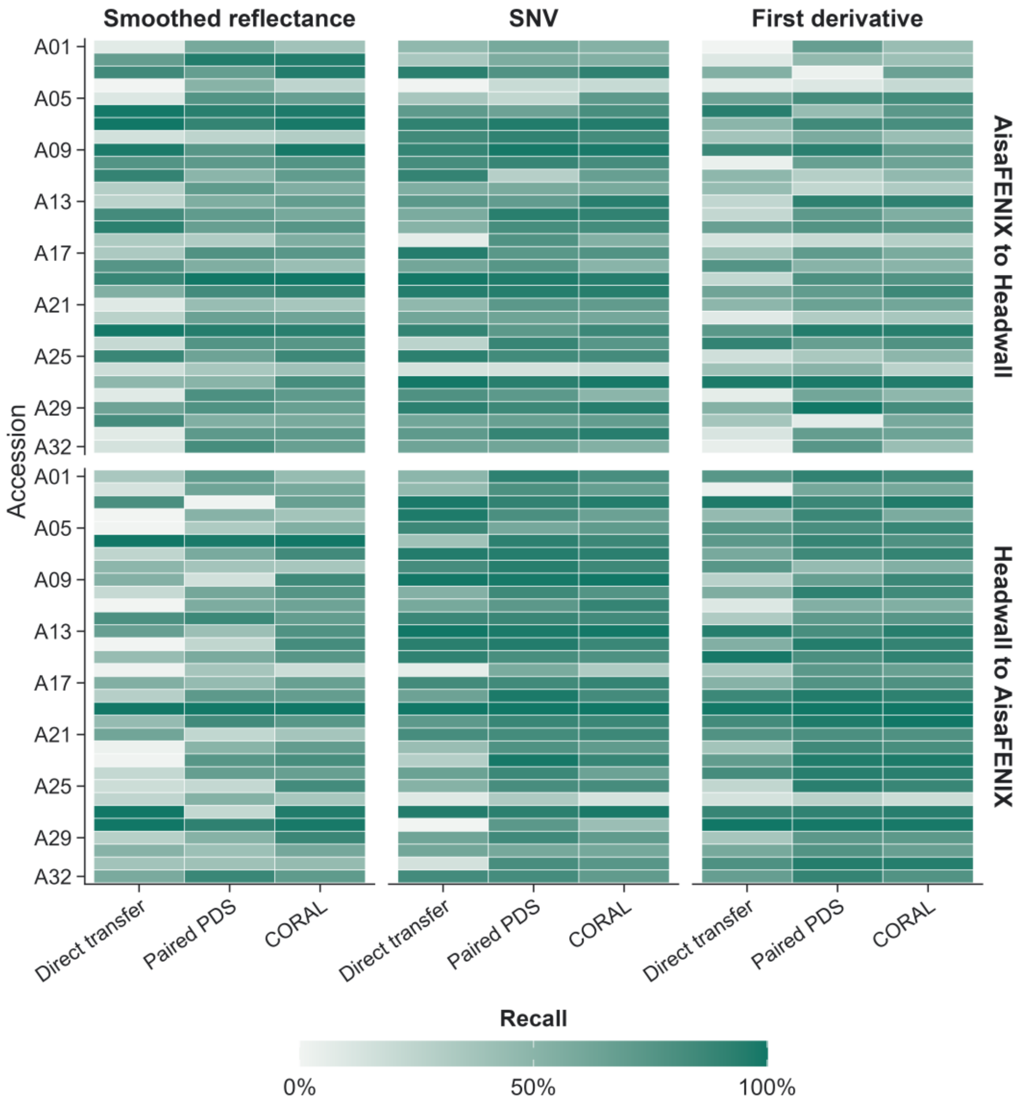

Figure S4. Accession-specific cross-sensor recall. Mean recall for each of the 32 accessions, all three spectral representations, both transfer directions, and the direct-transfer, PDS, and CORAL scenarios across pair-preserving resamples.

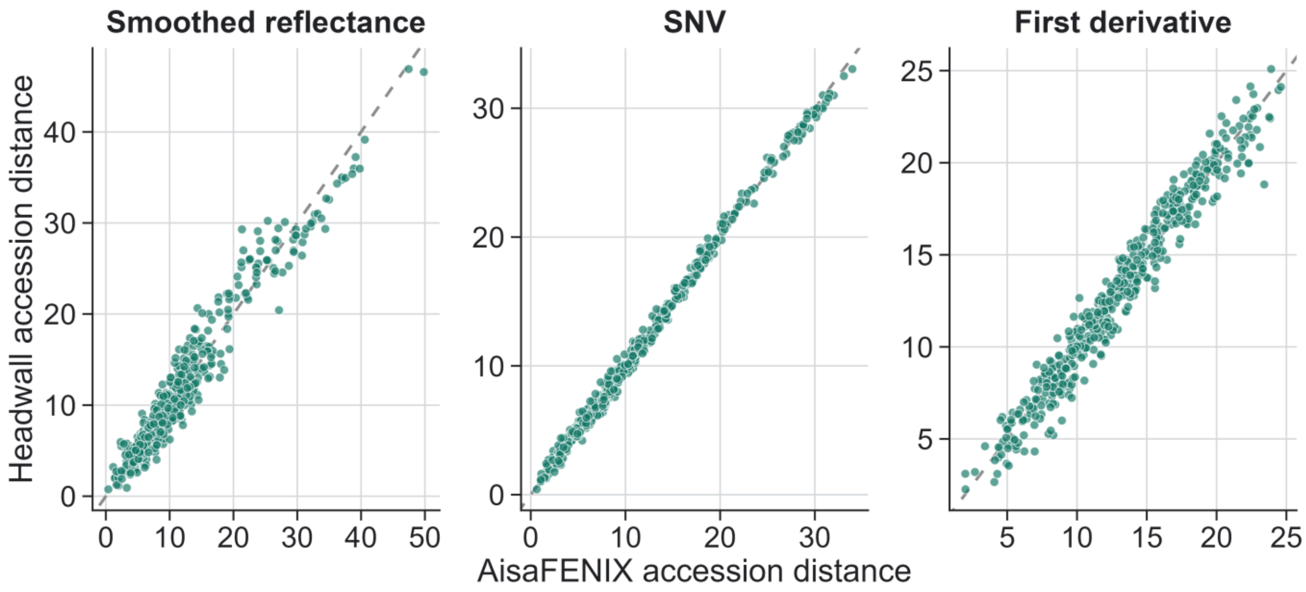

Figure S5. Accession-distance concordance for the three spectral representations. All 496 pairwise Euclidean distances between wavelength-standardised accession centroids are plotted for AisaFENIX versus Headwall for reflectance, SNV and first derivatives. Individual-seed retrieval is reported in Figure S2 and Table S2A; accession-centroid top-1 retrieval is reported separately in Table S2B. The dashed line denotes the identity line.

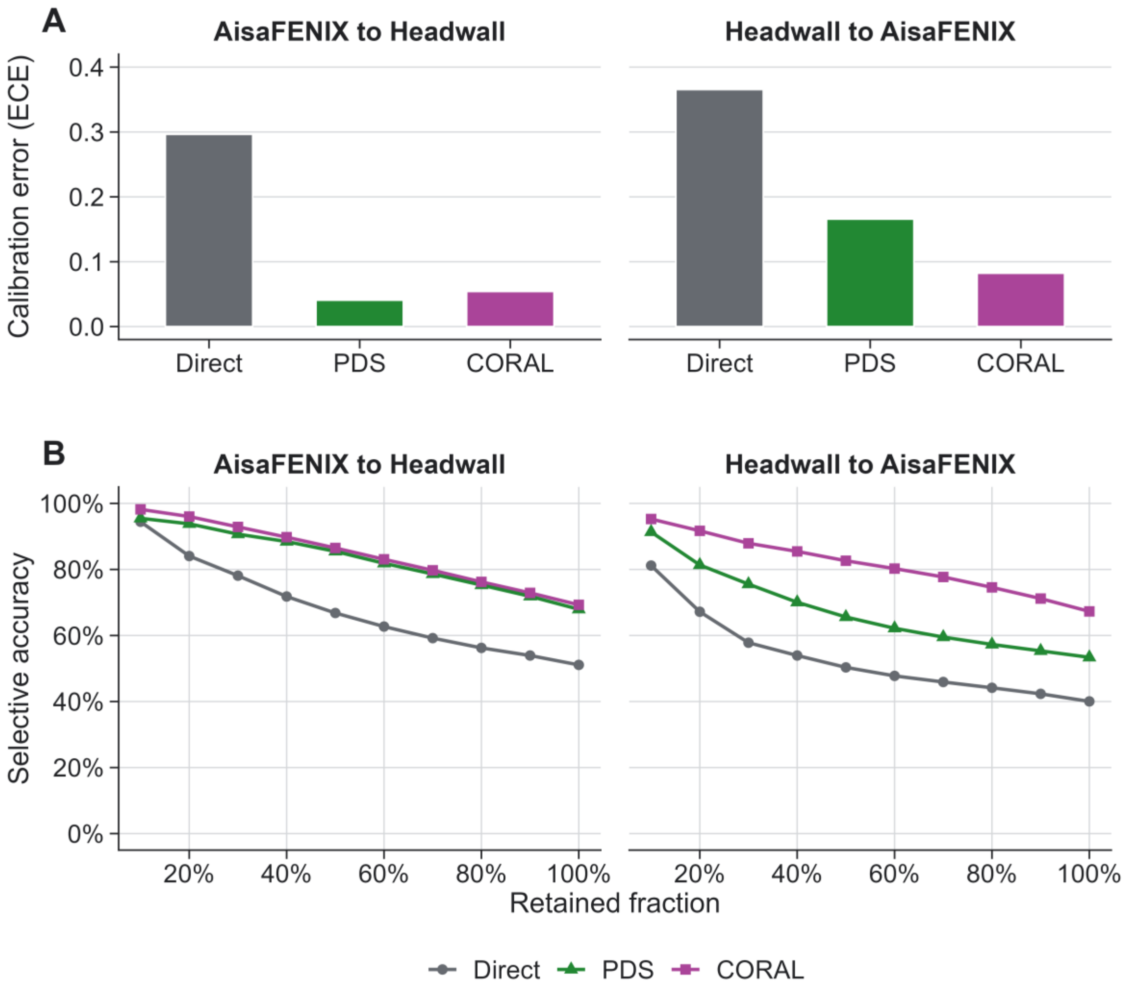

Figure S6. Probability calibration and selective prediction under sensor shift. Smoothed-reflectance results for direct transfer, PDS, and CORAL. (A) Expected calibration error. (B) Selective accuracy as the retained fraction increases. Accession-specific conformal coverage and prediction-set sizes are reported in Figure S7 and Table S6; that analysis is exploratory and does not provide a target-domain coverage guarantee.

sensors MDPI

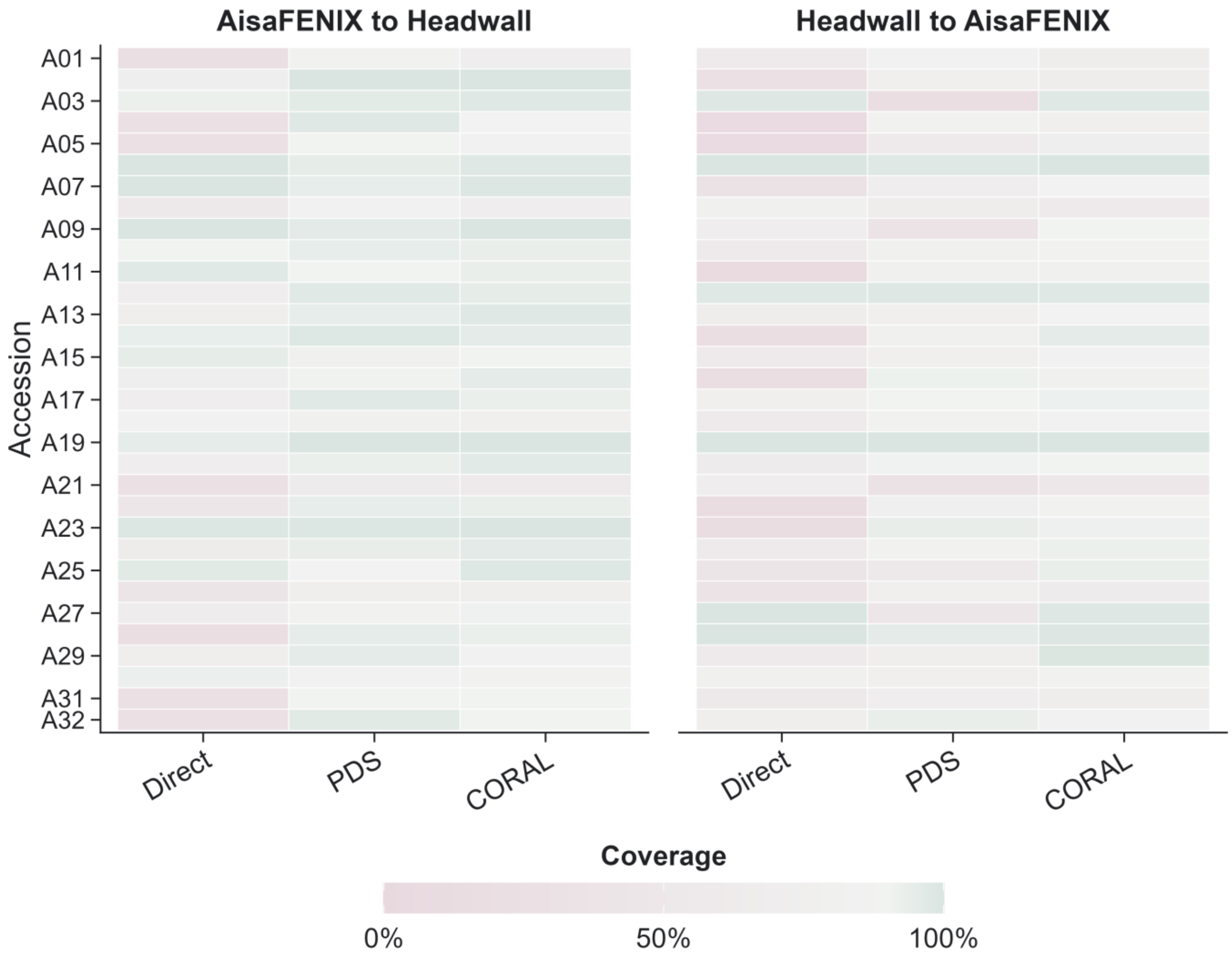

Figure S7. Accession-specific conformal coverage. Smoothed-reflectance true-label coverage for source-calibrated conformal sets by accession, direction, and transfer scenario. Coverage is exploratory and is not a target-domain guarantee.

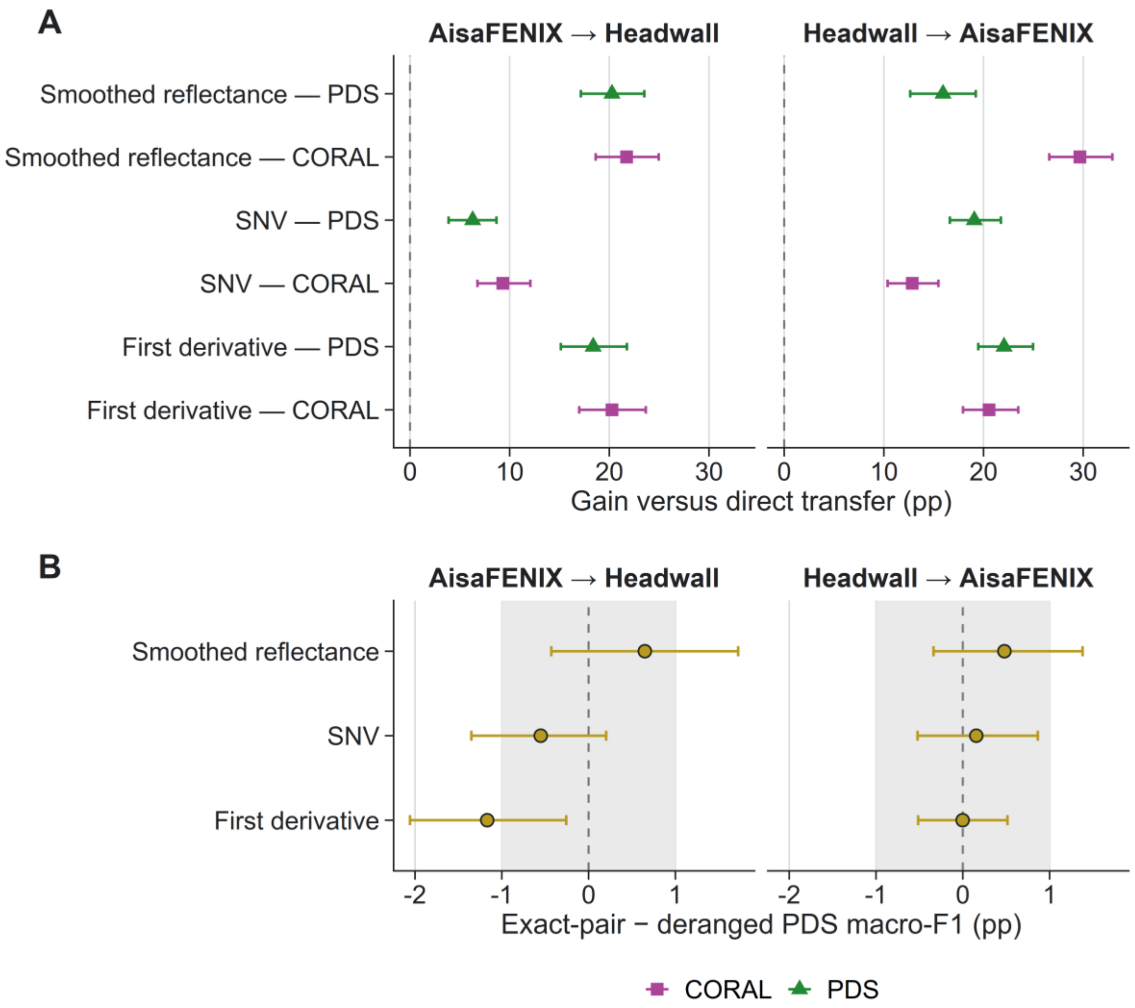

Figure S8. Fixed-split calibration-transfer gains and exploratory pairing intervals. (A) Macro-F1 differences for PDS and CORAL versus direct transfer on the fixed 70:30 split; bars show accession-stratified paired-bootstrap 95% intervals, and Holm adjustment covered 12 adaptation-versus-direct-transfer contrasts. (B) Same-seed minus within-accession-deranged PDS differences; bars show 90% intervals, and the shaded ±1-percentage-point band is illustrative rather than a validated equivalence margin. Intervals are conditional on one split and one derangement and exclude uncertainty from additional lots, acquisitions, or instruments. Repeated derangements are summarised in Table S12.

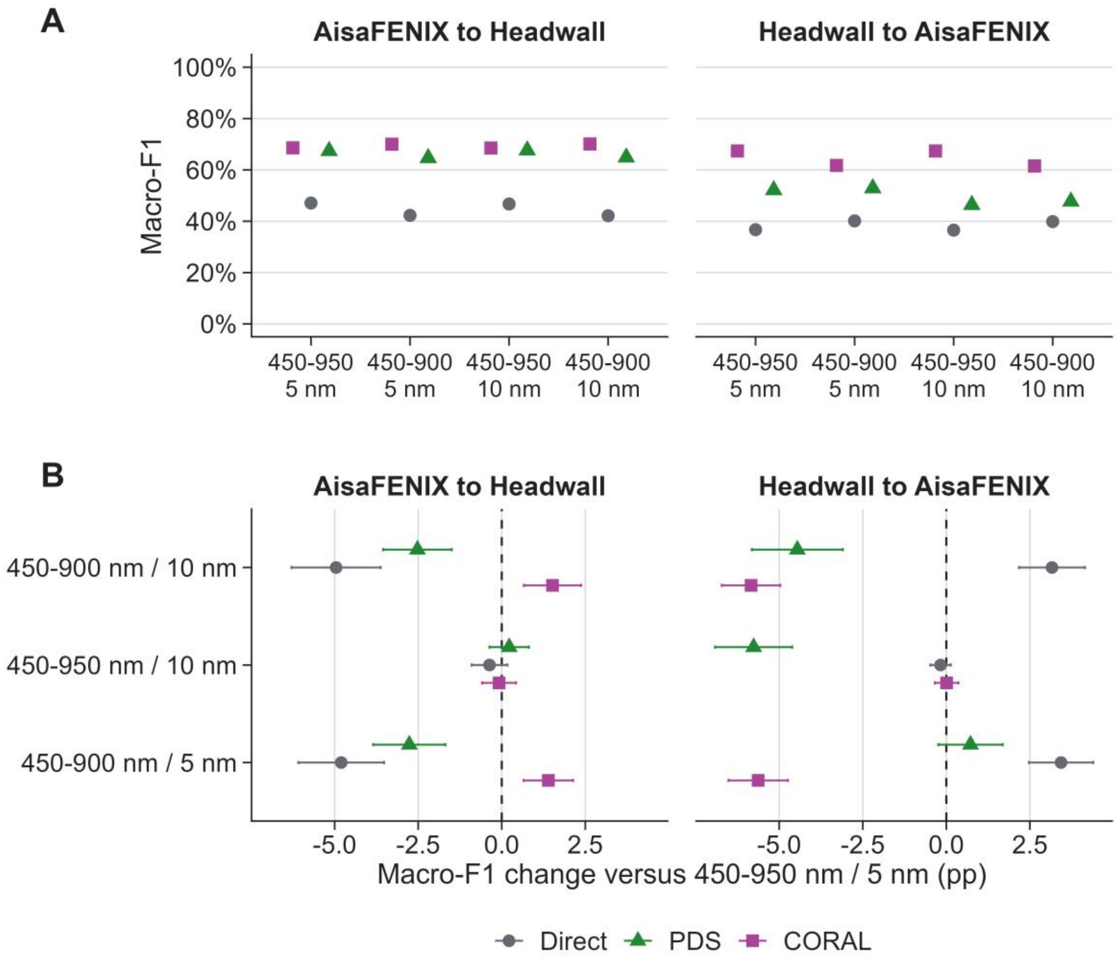

Figure S9. Sensitivity to numerical common support and sampling density. (A) Smoothed-reflectance macro-F1 for the 450–900 and 450–950 nm supports at 5 and 10 nm sampling intervals. (B) Paired changes relative to 450–950 nm at 5 nm. Error bars are descriptive standard deviations across 30 pair-preserving resamples. This analysis does not model sensor spectral-response functions or FWHM and is therefore not an optical-resolution harmonisation test.

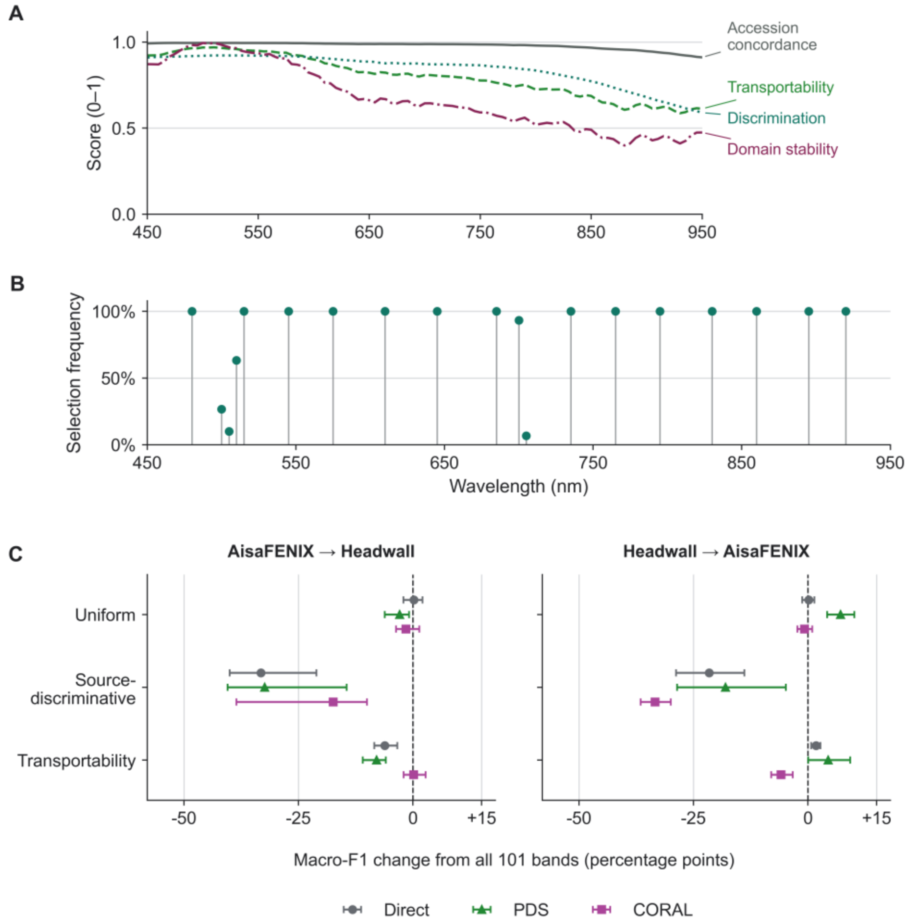

Figure S10. Spectral basis, stability, and predictive value of compact wavelength panels. (A) Bidirectionally averaged discrimination, domain-stability, accession-concordance and composite transportability profiles, identified directly and by line style. (B) Frequency with which each wavelength entered the 16-band transportability panel across 30 development resamples in each direction. (C) Mean paired macro-F1 change relative to all 101 common-interval bands for uniform, source-univariate-ranked (labelled 'Source-discriminative') and transportability-oriented 16-band subsets. Bars show the empirical 2.5th–97.5th percentiles of the 30 paired differences: descriptive central 95% ranges across overlapping splits, not confidence intervals. The additional mRMR comparator is reported in Table S24.

Supplementary Tables

Tables S1–S26 contain the supporting numerical results, consolidated parameters, and additional sensitivity and comparator analyses described in Supplementary Methods S1.

Table S1. Dataset dimensions and descriptive distributions after numerical wavelength alignment.

Panel A. Native dataset dimensions and recorded data properties.

Instrument	Seed spectra (n)	Accessions (n)	Seeds per accession	Native wavelengths (n)	Native range (nm)	Median interval (nm)	Missing/non-finite (n)	Duplicate spectra (n)	Duplicate identifiers (n)	Outside 0–1 within 450–950 nm (n)
AisaFENIX	3,200	32	100 for every accession	625	377.27–2502.75	1.72	0	0	0	0
Headwall	3,200	32	100 for every accession	825	423.34–1022.07	0.73	0	0	0	0

Panel B. Descriptive distributions after common-grid alignment.

Representation	Instrument	Spectra (n)	Coordinates (n)	Finite entries	Minimum	Maximum	Mean	SD
Smoothed reflectance	AisaFENIX	3,200	101	100.0%	0.016	0.795	0.305	0.200
Smoothed reflectance	Headwall	3,200	101	100.0%	0.003	0.919	0.367	0.250
SNV	AisaFENIX	3,200	101	100.0%	-2.536	3.162	0.000	0.995
SNV	Headwall	3,200	101	100.0%	-2.711	3.825	0.000	0.995
First derivative	AisaFENIX	3,200	101	100.0%	-0.002	0.007	0.001	0.001
First derivative	Headwall	3,200	101	100.0%	-0.011	0.009	0.001	0.001

Note: All 3,200 spectra per instrument were retained, representing one spectrum per physical seed and sensor. The 625 AisaFENIX and 825 Headwall native wavelength channels describe spectral sampling; the ROI pixel counts in Section S1.7 describe spatial sampling. Reflectance and SNV are dimensionless; first derivatives are expressed as reflectance per nm. Interpolation onto 101 common coordinates from 450 to 950 nm at 5 nm intervals aligned the wavelength grids but did not equalise optical FWHM or spectral-response functions. Acquisition-specific FWHM, ground-sampling distance, and signal-to-noise ratio were unavailable for this analysis.

Table S2. Same-seed spectral similarity and retrieval and cross-instrument preservation of accession–lot geometry.

Panel A. Pooled same-seed similarity, retrieval, and similarity permutation tests.

Representation	Analysis	Same-seed pairs (n)	Same-seed similarity	Non-match similarity	Difference	Top-1: AisaFENIX to Headwall	Top-1: Headwall to AisaFENIX	Random top-1	Similarity permutation p
Smoothed reflectance	Full spectrum	3,200	0.995	0.995	0.00005	0.8%	1.1%	1.0%	0.1430
Smoothed reflectance	Within-accession residual	3,200	0.014	0.006	0.00801	1.2%	1.4%	1.0%	0.2800
SNV	Full spectrum	3,200	0.995	0.995	0.00005	0.8%	1.1%	1.0%	0.1520
SNV	Within-accession residual	3,200	0.011	0.003	0.00788	0.9%	0.8%	1.0%	0.2000
First derivative	Full spectrum	3,200	0.712	0.713	-0.00018	1.0%	1.0%	1.0%	0.6690
First derivative	Within-accession residual	3,200	-0.003	0.000	-0.00359	0.8%	0.9%	1.0%	0.8400

Panel B. Accession-centroid similarity and bidirectional retrieval.

Representation	Accessions (n)	Matched-centroid similarity	Non-match similarity	Difference	Top-1: AisaFENIX to Headwall	Top-1: Headwall to AisaFENIX	Random top-1
Smoothed reflectance	32	0.999	0.926	0.07315	68.8%	78.1%	3.1%
SNV	32	0.999	0.925	0.07399	75.0%	81.2%	3.1%
First derivative	32	0.859	0.434	0.42515	31.2%	56.2%	3.1%

Panel C. Mantel, Procrustes, and RV concordance statistics.

Representation	Accessions (n)	Mantel rho	Mantel p	Procrustes r	Procrustes p	RV coefficient
Smoothed reflectance	32	0.958	0.0010	0.981	0.0010	0.963
SNV	32	0.996	0.0010	0.997	0.0010	0.997
First derivative	32	0.982	0.0010	0.979	0.0010	0.984
Consensus rank distance	32	0.989	0.0010	0.981	0.0010	0.990

Note: The uniform-random top-1 reference was 1% for individual seeds and 3.125% for accession centroids. The p values in Panel A test mean same-seed similarity, not top-1 retrieval; these upper-tail tests used 999 within-accession permutations and were exploratory without multiplicity adjustment. Permutation p values had a minimum attainable value of 0.001. The consensus distance summary is computed from the same representations and is not independent validation.

Table S3. Cross-instrument classification performance and residual distribution shift after calibration transfer.

Panel A. Repeated cross-instrument accuracy and macro-F1.

Representation	Direction	Scenario	Accuracy mean	Accuracy SD	Macro-F1 mean	Macro-F1 SD	Splits (n)
Smoothed reflectance	AisaFENIX to Headwall	Same-sensor reference	0.732	0.014	0.728	0.015	30
Smoothed reflectance	AisaFENIX to Headwall	Direct transfer	0.516	0.010	0.471	0.011	30
Smoothed reflectance	AisaFENIX to Headwall	PDS	0.677	0.012	0.674	0.012	30
Smoothed reflectance	AisaFENIX to Headwall	CORAL	0.695	0.012	0.686	0.013	30
Smoothed reflectance	Headwall to AisaFENIX	Same-sensor reference	0.753	0.011	0.749	0.011	30
Smoothed reflectance	Headwall to AisaFENIX	Direct transfer	0.407	0.009	0.367	0.009	30
Smoothed reflectance	Headwall to AisaFENIX	PDS	0.536	0.013	0.522	0.012	30
Smoothed reflectance	Headwall to AisaFENIX	CORAL	0.679	0.011	0.674	0.012	30
SNV	AisaFENIX to Headwall	Same-sensor reference	0.824	0.012	0.822	0.013	30
SNV	AisaFENIX to Headwall	Direct transfer	0.660	0.011	0.646	0.012	30
SNV	AisaFENIX to Headwall	PDS	0.716	0.013	0.707	0.014	30
SNV	AisaFENIX to Headwall	CORAL	0.735	0.012	0.733	0.012	30
SNV	Headwall to AisaFENIX	Same-sensor reference	0.853	0.009	0.852	0.009	30
SNV	Headwall to AisaFENIX	Direct transfer	0.657	0.014	0.629	0.016	30
SNV	Headwall to AisaFENIX	PDS	0.833	0.011	0.834	0.011	30
SNV	Headwall to AisaFENIX	CORAL	0.773	0.012	0.770	0.013	30
First derivative	AisaFENIX to Headwall	Same-sensor reference	0.918	0.008	0.917	0.008	30
First derivative	AisaFENIX to Headwall	Direct transfer	0.409	0.012	0.379	0.012	30
First derivative	AisaFENIX to Headwall	PDS	0.591	0.011	0.580	0.012	30
First derivative	AisaFENIX to Headwall	CORAL	0.600	0.015	0.605	0.015	30
First derivative	Headwall to AisaFENIX	Same-sensor reference	0.853	0.010	0.851	0.010	30
First derivative	Headwall to AisaFENIX	Direct transfer	0.614	0.016	0.608	0.018	30
First derivative	Headwall to AisaFENIX	PDS	0.815	0.011	0.813	0.011	30
First derivative	Headwall to AisaFENIX	CORAL	0.806	0.013	0.805	0.013	30

Panel B. Mean-vector and normalised covariance gaps in pooled z-space.

Representation	Direction	Scenario	Mean gap mean	Mean gap SD	Covariance gap mean	Covariance gap SD
Smoothed reflectance	AisaFENIX to Headwall	Direct transfer	4.937	0.054	0.306	0.010
Smoothed reflectance	AisaFENIX to Headwall	PDS	0.000	0.000	0.518	0.011
Smoothed reflectance	AisaFENIX to Headwall	CORAL	0.000	0.000	0.000	0.000
Smoothed reflectance	Headwall to AisaFENIX	Direct transfer	4.937	0.054	0.439	0.020
Smoothed reflectance	Headwall to AisaFENIX	PDS	0.000	0.000	0.388	0.006
Smoothed reflectance	Headwall to AisaFENIX	CORAL	0.000	0.000	0.000	0.000
SNV	AisaFENIX to Headwall	Direct transfer	1.889	0.011	0.096	0.003
SNV	AisaFENIX to Headwall	PDS	0.000	0.000	0.123	0.003
SNV	AisaFENIX to Headwall	CORAL	0.000	0.000	0.001	0.000
SNV	Headwall to AisaFENIX	Direct transfer	1.889	0.011	0.099	0.004
SNV	Headwall to AisaFENIX	PDS	0.000	0.000	0.102	0.003
SNV	Headwall to AisaFENIX	CORAL	0.000	0.000	0.000	0.000
First derivative	AisaFENIX to Headwall	Direct transfer	5.423	0.017	0.457	0.002
First derivative	AisaFENIX to Headwall	PDS	0.000	0.000	0.780	0.008
First derivative	AisaFENIX to Headwall	CORAL	0.000	0.000	0.003	0.000
First derivative	Headwall to AisaFENIX	Direct transfer	5.423	0.017	0.719	0.007
First derivative	Headwall to AisaFENIX	PDS	0.000	0.000	0.450	0.005
First derivative	Headwall to AisaFENIX	CORAL	0.000	0.000	0.000	0.000

Note: Values are means and descriptive SDs across 30 overlapping pair-preserving splits. SDs are not confidence intervals, and the splits are not independent biological replicates. Accuracy equalled balanced accuracy because every test split contained the same number of seeds per accession. The near-zero mean and covariance gaps after CORAL are expected for an alignment fitted on calibration-domain data and do not prove external equivalence. For smoothed reflectance in the AisaFENIX-to-Headwall direction, the covariance gap increased from 0.306 with direct transfer to 0.518 with PDS, showing that mean correction need not reduce covariance mismatch.

Table S4. Effect of target calibration-set size and exact versus deranged pairing on cross-instrument macro-F1.

Panel A. Smoothed-reflectance macro-F1 by target calibration-set size.

Direction	Method	Calibration spectra per accession (n)	Total calibration spectra (n)	Macro-F1 mean	Macro-F1 SD	Splits (n)
AisaFENIX to Headwall	Direct transfer	0	0	0.471	0.011	30
AisaFENIX to Headwall	PDS	1	32	0.496	0.056	30
AisaFENIX to Headwall	CORAL	1	32	0.659	0.027	30
AisaFENIX to Headwall	PDS	2	64	0.554	0.053	30
AisaFENIX to Headwall	CORAL	2	64	0.673	0.018	30
AisaFENIX to Headwall	PDS	5	160	0.626	0.028	30
AisaFENIX to Headwall	CORAL	5	160	0.680	0.015	30
AisaFENIX to Headwall	PDS	10	320	0.652	0.020	30
AisaFENIX to Headwall	CORAL	10	320	0.684	0.015	30
AisaFENIX to Headwall	PDS	20	640	0.666	0.018	30
AisaFENIX to Headwall	CORAL	20	640	0.683	0.015	30
AisaFENIX to Headwall	PDS	40	1,280	0.673	0.014	30
AisaFENIX to Headwall	CORAL	40	1,280	0.686	0.014	30
AisaFENIX to Headwall	PDS	70	2,240	0.674	0.012	30
AisaFENIX to Headwall	CORAL	70	2,240	0.686	0.013	30
Headwall to AisaFENIX	Direct transfer	0	0	0.367	0.009	30
Headwall to AisaFENIX	PDS	1	32	0.407	0.064	30
Headwall to AisaFENIX	CORAL	1	32	0.615	0.037	30
Headwall to AisaFENIX	PDS	2	64	0.459	0.047	30
Headwall to AisaFENIX	CORAL	2	64	0.642	0.035	30
Headwall to AisaFENIX	PDS	5	160	0.503	0.032	30
Headwall to AisaFENIX	CORAL	5	160	0.657	0.025	30
Headwall to AisaFENIX	PDS	10	320	0.506	0.029	30
Headwall to AisaFENIX	CORAL	10	320	0.662	0.027	30
Headwall to AisaFENIX	PDS	20	640	0.515	0.016	30
Headwall to AisaFENIX	CORAL	20	640	0.673	0.014	30
Headwall to AisaFENIX	PDS	40	1,280	0.519	0.016	30
Headwall to AisaFENIX	CORAL	40	1,280	0.674	0.013	30

Direction	Method	Calibration spectra per accession (n)	Total calibration spectra (n)	Macro-F1 mean	Macro-F1 SD	Splits (n)
Headwall to AisaFENIX	PDS	70	2,240	0.522	0.012	30
Headwall to AisaFENIX	CORAL	70	2,240	0.674	0.012	30

Panel B. Same-seed versus within-accession shuffled PDS.

Representation	Direction	Same-seed PDS mean	Same-seed PDS SD	Within-accession shuffled PDS mean	Within-accession shuffled PDS SD	Difference mean (pp)	Difference SD (pp)	Minimum difference (pp)	Maximum difference (pp)	Exact-pair win rate
Smoothed reflectance	AisaFENIX to Headwall	0.674	0.012	0.673	0.012	0.15	0.60	-1.07	1.34	63.3%
Smoothed reflectance	Headwall to AisaFENIX	0.522	0.012	0.510	0.014	1.13	0.73	-0.60	2.61	90.0%
SNV	AisaFENIX to Headwall	0.707	0.014	0.708	0.013	-0.10	0.49	-1.05	0.92	46.7%
SNV	Headwall to AisaFENIX	0.834	0.011	0.832	0.010	0.20	0.43	-0.66	1.32	66.7%
First derivative	AisaFENIX to Headwall	0.580	0.012	0.577	0.012	0.31	0.61	-0.82	1.92	70.0%
First derivative	Headwall to AisaFENIX	0.813	0.011	0.811	0.011	0.19	0.37	-0.48	0.80	73.3%

Note: SDs describe variation across 30 overlapping splits, which are summarised descriptively. CORAL did not use class labels in its alignment objective, but its target calibration subsets were selected with known accession labels to enforce balance. Table S12 expands the PDS pairing control to five derangements per split.

Table S5. Selection stability and predictive performance of reduced wavelength panels.

Panel A. Selection frequency of the transportability-oriented wavelengths.

Wavelength (nm)	Selection frequency	Selected splits (n/30)
480	100.0%	30
500	26.7%	8
505	10.0%	3
510	63.3%	19
515	100.0%	30
545	100.0%	30
575	100.0%	30
610	100.0%	30
645	100.0%	30
685	100.0%	30
700	93.3%	28
705	6.7%	2
735	100.0%	30
765	100.0%	30
795	100.0%	30
830	100.0%	30
860	100.0%	30
895	100.0%	30
920	100.0%	30

Panel B. Macro-F1 for full-grid and 16-wavelength strategies.

Direction	Panel	Scenario	Wavelengths (n)	Macro-F1 mean	Macro-F1 SD
AisaFENIX to Headwall	Full grid	Same-sensor reference	101	0.721	0.012
AisaFENIX to Headwall	Full grid	Direct transfer	101	0.471	0.012
AisaFENIX to Headwall	Full grid	PDS	101	0.673	0.011
AisaFENIX to Headwall	Full grid	CORAL	101	0.685	0.013
AisaFENIX to Headwall	Uniform 16	Same-sensor reference	16	0.720	0.010

Direction	Panel	Scenario	Wavelengths (n)	Macro-F1 mean	Macro-F1 SD
AisaFENIX to Headwall	Uniform 16	Direct transfer	16	0.473	0.014
AisaFENIX to Headwall	Uniform 16	PDS	16	0.644	0.015
AisaFENIX to Headwall	Uniform 16	CORAL	16	0.670	0.012
AisaFENIX to Headwall	Source-univariate-ranked 16	Same-sensor reference	16	0.431	0.098
AisaFENIX to Headwall	Source-univariate-ranked 16	Direct transfer	16	0.139	0.058
AisaFENIX to Headwall	Source-univariate-ranked 16	PDS	16	0.350	0.102
AisaFENIX to Headwall	Source-univariate-ranked 16	CORAL	16	0.511	0.084
AisaFENIX to Headwall	Transportability-oriented 16	Same-sensor reference	16	0.666	0.014
AisaFENIX to Headwall	Transportability-oriented 16	Direct transfer	16	0.409	0.014
AisaFENIX to Headwall	Transportability-oriented 16	PDS	16	0.594	0.016
AisaFENIX to Headwall	Transportability-oriented 16	CORAL	16	0.687	0.013
Headwall to AisaFENIX	Full grid	Same-sensor reference	101	0.749	0.011
Headwall to AisaFENIX	Full grid	Direct transfer	101	0.363	0.012
Headwall to AisaFENIX	Full grid	PDS	101	0.517	0.013
Headwall to AisaFENIX	Full grid	CORAL	101	0.667	0.011
Headwall to AisaFENIX	Uniform 16	Same-sensor reference	16	0.729	0.009
Headwall to AisaFENIX	Uniform 16	Direct transfer	16	0.364	0.012
Headwall to AisaFENIX	Uniform 16	PDS	16	0.588	0.012
Headwall to AisaFENIX	Uniform 16	CORAL	16	0.659	0.011
Headwall to AisaFENIX	Source-univariate-ranked 16	Same-sensor reference	16	0.513	0.109
Headwall to AisaFENIX	Source-univariate-ranked 16	Direct transfer	16	0.147	0.051
Headwall to AisaFENIX	Source-univariate-ranked 16	PDS	16	0.337	0.079
Headwall to AisaFENIX	Source-univariate-ranked 16	CORAL	16	0.333	0.016
Headwall to AisaFENIX	Transportability-oriented 16	Same-sensor reference	16	0.736	0.011
Headwall to AisaFENIX	Transportability-oriented 16	Direct transfer	16	0.380	0.012
Headwall to AisaFENIX	Transportability-oriented 16	PDS	16	0.561	0.020
Headwall to AisaFENIX	Transportability-oriented 16	CORAL	16	0.608	0.013

Note: Selection frequencies were calculated across 30 development partitions. Reduced panels were evaluated within the studied datasets and transfer pipelines. The source-univariate-ranked panel had weak same-sensor macro-F1 (0.431 and 0.513 in the two directions), so its weak transfer cannot be attributed solely to sensor shift. The transportability-oriented panel uses target-domain accession information for its internal selection. Full-grid values can differ slightly from Table 2 because the compact-panel analysis used separate pair-preserving splits.

Table S6. Probability calibration, conformal-set behaviour, and selective prediction under cross-instrument transfer using a separate 50/20/30 design.

Panel A. Probability-calibration metrics with descriptive SD.

Direction	Scenario	Accuracy mean	Accuracy SD	Macro-F1 mean	Macro-F1 SD	Log loss mean	Log loss SD	Brier score mean	Brier score SD	ECE mean	ECE SD
AisaFENIX to Headwall	Direct transfer	0.511	0.014	0.465	0.017	2.300	0.100	0.746	0.022	0.296	0.016
AisaFENIX to Headwall	PDS	0.679	0.013	0.676	0.014	0.995	0.050	0.437	0.012	0.041	0.011
AisaFENIX to Headwall	CORAL	0.693	0.009	0.684	0.011	1.014	0.055	0.424	0.010	0.054	0.009
Headwall to AisaFENIX	Direct transfer	0.400	0.013	0.362	0.014	2.966	0.125	0.907	0.022	0.365	0.015
Headwall to AisaFENIX	PDS	0.534	0.016	0.518	0.018	1.625	0.064	0.651	0.017	0.166	0.015
Headwall to AisaFENIX	CORAL	0.673	0.010	0.668	0.010	1.170	0.050	0.467	0.013	0.082	0.010

Panel B. Coverage, set size, singleton, and empty-set summaries.

Direction	Scenario	Evaluation domain	Method	Alpha	Coverage (mean ± SD)	Set size (mean ± SD)	Singleton rate (mean ± SD)	Singleton accuracy (mean ± SD)	Empty-set rate (mean ± SD)
AisaFENIX to Headwall	Direct transfer	Source	Standard	0.10	0.901 ± 0.019	1.883 ± 0.138	0.407 ± 0.049	0.906 ± 0.019	0.000 ± 0.000
AisaFENIX to Headwall	Direct transfer	Target	Standard	0.10	0.679 ± 0.023	1.641 ± 0.100	0.522 ± 0.044	0.655 ± 0.022	0.000 ± 0.000
AisaFENIX to Headwall	Direct transfer	Target	Importance weighted	0.10	0.665 ± 0.022	1.578 ± 0.083	0.551 ± 0.039	0.643 ± 0.023	0.000 ± 0.000
AisaFENIX to Headwall	PDS	Source	Standard	0.10	0.901 ± 0.019	1.883 ± 0.138	0.407 ± 0.049	0.906 ± 0.019	0.000 ± 0.000
AisaFENIX to Headwall	PDS	Target	Standard	0.10	0.910 ± 0.015	2.072 ± 0.159	0.316 ± 0.046	0.896 ± 0.019	0.000 ± 0.000
AisaFENIX to Headwall	PDS	Target	Importance weighted	0.10	0.910 ± 0.015	2.072 ± 0.159	0.316 ± 0.046	0.896 ± 0.019	0.000 ± 0.000

Direction	Scenario	Evaluation domain	Method	Alpha	Coverage (mean ± SD)	Set size (mean ± SD)	Singleton rate (mean ± SD)	Singleton accuracy (mean ± SD)	Empty-set rate (mean ± SD)
AisaFENIX to Headwall	CORAL	Source	Standard	0.10	0.902 ± 0.017	1.837 ± 0.124	0.430 ± 0.045	0.906 ± 0.019	0.000 ± 0.000
AisaFENIX to Headwall	CORAL	Target	Standard	0.10	0.900 ± 0.017	1.882 ± 0.124	0.397 ± 0.044	0.900 ± 0.020	0.000 ± 0.000
AisaFENIX to Headwall	CORAL	Target	Importance weighted	0.10	0.900 ± 0.017	1.882 ± 0.124	0.397 ± 0.044	0.900 ± 0.020	0.000 ± 0.000
Headwall to AisaFENIX	Direct transfer	Source	Standard	0.10	0.897 ± 0.016	1.637 ± 0.102	0.508 ± 0.049	0.899 ± 0.017	0.000 ± 0.000
Headwall to AisaFENIX	Direct transfer	Target	Standard	0.10	0.559 ± 0.030	1.660 ± 0.116	0.498 ± 0.051	0.491 ± 0.026	0.000 ± 0.000
Headwall to AisaFENIX	Direct transfer	Target	Importance weighted	0.10	0.596 ± 0.068	1.856 ± 0.333	0.440 ± 0.111	0.513 ± 0.047	0.000 ± 0.000
Headwall to AisaFENIX	PDS	Source	Standard	0.10	0.897 ± 0.016	1.637 ± 0.102	0.508 ± 0.049	0.899 ± 0.017	0.000 ± 0.000
Headwall to AisaFENIX	PDS	Target	Standard	0.10	0.751 ± 0.026	1.892 ± 0.148	0.380 ± 0.054	0.704 ± 0.034	0.000 ± 0.000
Headwall to AisaFENIX	PDS	Target	Importance weighted	0.10	0.751 ± 0.026	1.892 ± 0.148	0.380 ± 0.054	0.704 ± 0.034	0.000 ± 0.000
Headwall to AisaFENIX	CORAL	Source	Standard	0.10	0.896 ± 0.016	1.681 ± 0.114	0.487 ± 0.050	0.898 ± 0.016	0.000 ± 0.000
Headwall to AisaFENIX	CORAL	Target	Standard	0.10	0.844 ± 0.019	1.735 ± 0.122	0.460 ± 0.051	0.832 ± 0.021	0.000 ± 0.000
Headwall to AisaFENIX	CORAL	Target	Importance weighted	0.10	0.844 ± 0.019	1.735 ± 0.122	0.460 ± 0.051	0.832 ± 0.021	0.000 ± 0.000

Panel C. Across-accession distribution of conformal coverage.

Direction	Scenario	Evaluation domain	Method	Alpha	Worst accession	10th percentile	Median	Best accession
AisaFENIX to Headwall	Direct transfer	Source	Standard	0.10	0.559	0.784	0.919	1.000
AisaFENIX to Headwall	Direct transfer	Target	Standard	0.10	0.188	0.264	0.716	1.000
AisaFENIX to Headwall	Direct transfer	Target	Importance weighted	0.10	0.169	0.247	0.695	1.000

Direction	Scenario	Evaluation domain	Method	Alpha	Worst accession	10th percentile	Median	Best accession
AisaFENIX to Headwall	PDS	Source	Standard	0.10	0.559	0.784	0.919	1.000
AisaFENIX to Headwall	PDS	Target	Standard	0.10	0.640	0.825	0.943	1.000
AisaFENIX to Headwall	PDS	Target	Importance weighted	0.10	0.640	0.825	0.943	1.000
AisaFENIX to Headwall	CORAL	Source	Standard	0.10	0.512	0.801	0.918	1.000
AisaFENIX to Headwall	CORAL	Target	Standard	0.10	0.552	0.727	0.933	1.000
AisaFENIX to Headwall	CORAL	Target	Importance weighted	0.10	0.552	0.727	0.933	1.000
Headwall to AisaFENIX	Direct transfer	Source	Standard	0.10	0.573	0.749	0.929	1.000
Headwall to AisaFENIX	Direct transfer	Target	Standard	0.10	0.060	0.126	0.585	1.000
Headwall to AisaFENIX	Direct transfer	Target	Importance weighted	0.10	0.073	0.195	0.637	1.000
Headwall to AisaFENIX	PDS	Source	Standard	0.10	0.573	0.749	0.929	1.000
Headwall to AisaFENIX	PDS	Target	Standard	0.10	0.166	0.456	0.779	1.000
Headwall to AisaFENIX	PDS	Target	Importance weighted	0.10	0.166	0.456	0.779	1.000
Headwall to AisaFENIX	CORAL	Source	Standard	0.10	0.553	0.746	0.925	1.000
Headwall to AisaFENIX	CORAL	Target	Standard	0.10	0.488	0.658	0.874	1.000
Headwall to AisaFENIX	CORAL	Target	Importance weighted	0.10	0.488	0.658	0.874	1.000

Panel D. Selective accuracy at 10%, 50%, and 100% retained fractions.

Direction	Scenario	Retained fraction	Selective accuracy mean	Selective accuracy SD	Selective risk mean	Mean confidence cut-off
AisaFENIX to Headwall	Direct transfer	10.0%	0.944	0.019	0.056	1.000
AisaFENIX to Headwall	Direct transfer	50.0%	0.668	0.020	0.332	0.873
AisaFENIX to Headwall	Direct transfer	100.0%	0.511	0.014	0.489	0.280
AisaFENIX to Headwall	PDS	10.0%	0.955	0.021	0.045	0.993
AisaFENIX to Headwall	PDS	50.0%	0.855	0.015	0.145	0.680
AisaFENIX to Headwall	PDS	100.0%	0.679	0.013	0.321	0.244
AisaFENIX to Headwall	CORAL	10.0%	0.982	0.012	0.018	0.998
AisaFENIX to Headwall	CORAL	50.0%	0.865	0.009	0.135	0.759
AisaFENIX to Headwall	CORAL	100.0%	0.693	0.009	0.307	0.252
Headwall to AisaFENIX	Direct transfer	10.0%	0.812	0.029	0.188	1.000
Headwall to AisaFENIX	Direct transfer	50.0%	0.503	0.022	0.497	0.795
Headwall to AisaFENIX	Direct transfer	100.0%	0.400	0.013	0.600	0.264

Direction	Scenario	Retained fraction	Selective accuracy mean	Selective accuracy SD	Selective risk mean	Mean confidence cut-off
Headwall to AisaFENIX	PDS	10.0%	0.914	0.029	0.086	0.982
Headwall to AisaFENIX	PDS	50.0%	0.656	0.019	0.344	0.687
Headwall to AisaFENIX	PDS	100.0%	0.534	0.016	0.466	0.249
Headwall to AisaFENIX	CORAL	10.0%	0.953	0.017	0.047	0.999
Headwall to AisaFENIX	CORAL	50.0%	0.826	0.013	0.174	0.776
Headwall to AisaFENIX	CORAL	100.0%	0.673	0.010	0.327	0.265

Panel E. Domain AUC, effective sample size, weight ranges, and truncation.

Direction	Scenario	Domain AUC (mean ± SD)	Calibration ESS (mean ± SD)	Calibration ESS fraction (mean ± SD)	Target ESS (mean ± SD)	Target ESS fraction (mean ± SD)	Calibration raw-weight range	Target raw-weight range	Calibration truncated fraction (mean ± SD)	Target truncated fraction (mean ± SD)
AisaFENIX to Headwall	Direct transfer	0.822 ± 0.002	431.3 ± 6.8	0.674 ± 0.011	601.7 ± 10.1	0.627 ± 0.011	0.052–3.075	0.036–14.511	0.000 ± 0.000	0.001 ± 0.001
AisaFENIX to Headwall	PDS	0.485 ± 0.005	640.0 ± 0.0	1.000 ± 0.000	960.0 ± 0.0	1.000 ± 0.000	1.000–1.000	1.000–1.000	0.000 ± 0.000	0.000 ± 0.000
AisaFENIX to Headwall	CORAL	0.485 ± 0.004	640.0 ± 0.0	1.000 ± 0.000	960.0 ± 0.0	1.000 ± 0.000	1.000–1.000	1.000–1.000	0.000 ± 0.000	0.000 ± 0.000
Headwall to AisaFENIX	Direct transfer	0.822 ± 0.002	164.2 ± 18.7	0.257 ± 0.029	521.1 ± 20.8	0.543 ± 0.022	0.072–27.492	0.324–19.216	0.002 ± 0.002	0.000 ± 0.000
Headwall to AisaFENIX	PDS	0.483 ± 0.006	640.0 ± 0.0	1.000 ± 0.000	960.0 ± 0.0	1.000 ± 0.000	1.000–1.000	1.000–1.000	0.000 ± 0.000	0.000 ± 0.000
Headwall to AisaFENIX	CORAL	0.485 ± 0.004	640.0 ± 0.0	1.000 ± 0.000	960.0 ± 0.0	1.000 ± 0.000	1.000–1.000	1.000–1.000	0.000 ± 0.000	0.000 ± 0.000

Note: All analyses used smoothed reflectance and separate 50/20/30 fitting, source-calibration, and test partitions rather than the primary 70/30 design. ECE used 10 equal-width confidence bins. Conformal results were source-calibrated at $\alpha = 0.10$ and are exploratory under target-domain shift; they do not provide a formal target-domain coverage guarantee. Uniform estimated weights after PDS or CORAL can arise from the alignment construction and do not establish that the domains are identical.

Table S7. Distribution of accession-specific recall across the 32 evaluated accessions.

Representation	Direction	Scenario	Accessions (n)	Mean	SD	Minimum	10th percentile	Median	90th percentile	Maximum
Smoothed reflectance	AisaFENIX to Headwall	Direct transfer	32	0.516	0.354	0.000	0.082	0.513	0.973	1.000
Smoothed reflectance	AisaFENIX to Headwall	PDS	32	0.677	0.188	0.253	0.441	0.707	0.934	0.999
Smoothed reflectance	AisaFENIX to Headwall	CORAL	32	0.695	0.215	0.247	0.390	0.706	0.971	1.000
Smoothed reflectance	Headwall to AisaFENIX	Direct transfer	32	0.407	0.327	0.000	0.003	0.366	0.979	1.000
Smoothed reflectance	Headwall to AisaFENIX	PDS	32	0.536	0.253	0.008	0.222	0.526	0.871	0.996
Smoothed reflectance	Headwall to AisaFENIX	CORAL	32	0.679	0.208	0.182	0.369	0.693	0.953	1.000
SNV	AisaFENIX to Headwall	Direct transfer	32	0.660	0.281	0.004	0.269	0.713	0.946	0.994
SNV	AisaFENIX to Headwall	PDS	32	0.716	0.229	0.147	0.314	0.741	0.944	0.990
SNV	AisaFENIX to Headwall	CORAL	32	0.735	0.211	0.206	0.507	0.792	0.947	0.984
SNV	Headwall to AisaFENIX	Direct transfer	32	0.657	0.302	0.000	0.160	0.685	0.979	1.000
SNV	Headwall to AisaFENIX	PDS	32	0.833	0.145	0.326	0.613	0.851	0.979	1.000
SNV	Headwall to AisaFENIX	CORAL	32	0.773	0.195	0.133	0.608	0.828	0.978	1.000
First derivative	AisaFENIX to Headwall	Direct transfer	32	0.409	0.297	0.000	0.050	0.398	0.857	0.979
First derivative	AisaFENIX to Headwall	PDS	32	0.591	0.276	0.023	0.203	0.670	0.930	0.999
First derivative	AisaFENIX to Headwall	CORAL	32	0.600	0.210	0.212	0.323	0.604	0.857	0.959

Representation	Direction	Scenario	Accessions (n)	Mean	SD	Minimum	10th percentile	Median	90th percentile	Maximum
First derivative	Headwall to AisaFE-NIX	Direct transfer	32	0.614	0.281	0.032	0.238	0.687	0.964	1.000
First derivative	Headwall to AisaFE-NIX	PDS	32	0.815	0.166	0.268	0.611	0.861	0.961	1.000
First derivative	Headwall to AisaFE-NIX	CORAL	32	0.806	0.177	0.180	0.579	0.857	0.973	1.000

Note: Recall summaries pool predictions across overlapping repeated splits of the 32 accession seed lots observed in this study.

Table S8. Fixed-split macro-F1 estimates, paired transfer gains, and exploratory calibration-pair comparisons.

Panel A. Observed macro-F1 and conditional paired-bootstrap intervals.

Representation	Direction	Scenario	Macro-F1	95% CI lower	95% CI upper	Test seeds (n)
Smoothed reflectance	AisaFENIX to Headwall	Direct transfer	0.471	0.444	0.495	960
Smoothed reflectance	AisaFENIX to Headwall	PDS	0.674	0.646	0.700	960
Smoothed reflectance	AisaFENIX to Headwall	CORAL	0.688	0.660	0.714	960
Smoothed reflectance	Headwall to AisaFENIX	Direct transfer	0.373	0.347	0.397	960
Smoothed reflectance	Headwall to AisaFENIX	PDS	0.533	0.502	0.560	960
Smoothed reflectance	Headwall to AisaFENIX	CORAL	0.670	0.643	0.695	960
SNV	AisaFENIX to Headwall	Direct transfer	0.644	0.618	0.667	960
SNV	AisaFENIX to Headwall	PDS	0.707	0.680	0.731	960
SNV	AisaFENIX to Headwall	CORAL	0.737	0.711	0.761	960
SNV	Headwall to AisaFENIX	Direct transfer	0.643	0.617	0.666	960
SNV	Headwall to AisaFENIX	PDS	0.834	0.811	0.854	960
SNV	Headwall to AisaFENIX	CORAL	0.771	0.747	0.794	960
First derivative	AisaFENIX to Headwall	Direct transfer	0.384	0.355	0.410	960
First derivative	AisaFENIX to Headwall	PDS	0.567	0.539	0.592	960
First derivative	AisaFENIX to Headwall	CORAL	0.586	0.557	0.614	960
First derivative	Headwall to AisaFENIX	Direct transfer	0.613	0.585	0.638	960
First derivative	Headwall to AisaFENIX	PDS	0.834	0.811	0.855	960
First derivative	Headwall to AisaFENIX	CORAL	0.819	0.796	0.841	960

Panel B. PDS and CORAL gains versus direct transfer with Holm adjustment.

Representation	Direction	Method	Difference (pp)	95% CI lower (pp)	95% CI upper (pp)	Bootstrap p	Holm-adjusted p	Significant at 0.05
Smoothed reflectance	AisaFENIX to Headwall	PDS	20.27	17.14	23.51	0.0002	0.0024	Yes
Smoothed reflectance	AisaFENIX to Headwall	CORAL	21.73	18.63	24.96	0.0002	0.0024	Yes
Smoothed reflectance	Headwall to AisaFENIX	PDS	15.94	12.64	19.22	0.0002	0.0024	Yes
Smoothed reflectance	Headwall to AisaFENIX	CORAL	29.67	26.60	32.93	0.0002	0.0024	Yes
SNV	AisaFENIX to Headwall	PDS	6.29	3.87	8.68	0.0002	0.0024	Yes
SNV	AisaFENIX to Headwall	CORAL	9.30	6.77	12.08	0.0002	0.0024	Yes

Representation	Direction	Method	Difference (pp)	95% CI lower (pp)	95% CI upper (pp)	Bootstrap p	Holm-adjusted p	Significant at 0.05
SNV	Headwall to AisaFENIX	PDS	19.07	16.62	21.74	0.0002	0.0024	Yes
SNV	Headwall to AisaFENIX	CORAL	12.84	10.38	15.48	0.0002	0.0024	Yes
First derivative	AisaFENIX to Headwall	PDS	18.38	15.14	21.76	0.0002	0.0024	Yes
First derivative	AisaFENIX to Headwall	CORAL	20.26	16.98	23.66	0.0002	0.0024	Yes
First derivative	Headwall to AisaFENIX	PDS	22.06	19.47	24.97	0.0002	0.0024	Yes
First derivative	Headwall to AisaFENIX	CORAL	20.55	17.94	23.48	0.0002	0.0024	Yes

Panel C. Same-seed versus within-accession-deranged PDS differences against an illustrative ±1-percentage-point band.

Representation	Direction	Same-seed PDS	Within-accession shuffled PDS	Difference (pp)	90% CI lower (pp)	90% CI upper (pp)	Illustrative band (pp)	90% interval within band?
Smoothed reflectance	AisaFENIX to Head-wall	0.674	0.667	0.65	-0.43	1.72	1.00	No
Smoothed reflectance	Headwall to AisaFE-NIX	0.533	0.528	0.48	-0.34	1.38	1.00	No
SNV	AisaFENIX to Head-wall	0.707	0.712	-0.55	-1.35	0.20	1.00	No
SNV	Headwall to AisaFE-NIX	0.834	0.832	0.16	-0.52	0.86	1.00	Yes
First derivative	AisaFENIX to Head-wall	0.567	0.579	-1.17	-2.06	-0.26	1.00	No
First derivative	Headwall to AisaFE-NIX	0.834	0.834	0.00	-0.51	0.52	1.00	Yes

Note: Intervals were obtained from 4,999 paired, accession-stratified bootstrap replicates based on one fixed, 960-seed test split. Panels A and B use 95% intervals, and Holm adjustment covers the 12 adaptation-versus-direct-transfer contrasts. With the plus-one convention, 0.0002 was the smallest attainable unadjusted p value. Panel C uses 90% intervals to display the magnitude of the same-seed-versus-deranged calibration difference against the illustrative ±1-percentage-point band; this is a separate exploratory comparison, not a confirmatory equivalence conclusion. The intervals do not account for a new split, derangement, seed lot, or instrument.

Table S9. Sensitivity of cross-instrument macro-F1 to numerical wavelength support and sampling interval.

Numerical grid	Coordinates (n)	Direction	Method	Macro-F1 mean	Macro-F1 SD	Difference from reference mean (pp)	Difference from reference SD (pp)
450–950 nm / 5 nm	101	AisaFENIX to Headwall	Direct transfer	0.471	0.011	0.00	0.00
450–950 nm / 5 nm	101	AisaFENIX to Headwall	PDS	0.674	0.012	0.00	0.00
450–950 nm / 5 nm	101	AisaFENIX to Headwall	CORAL	0.686	0.013	0.00	0.00
450–900 nm / 5 nm	91	AisaFENIX to Headwall	Direct transfer	0.423	0.013	-4.80	1.28
450–900 nm / 5 nm	91	AisaFENIX to Headwall	PDS	0.646	0.015	-2.77	1.08
450–900 nm / 5 nm	91	AisaFENIX to Headwall	CORAL	0.700	0.012	1.39	0.74
450–950 nm / 10 nm	51	AisaFENIX to Headwall	Direct transfer	0.467	0.011	-0.37	0.54
450–950 nm / 10 nm	51	AisaFENIX to Headwall	PDS	0.676	0.012	0.22	0.59
450–950 nm / 10 nm	51	AisaFENIX to Headwall	CORAL	0.685	0.012	-0.08	0.51
450–900 nm / 10 nm	46	AisaFENIX to Headwall	Direct transfer	0.421	0.014	-4.96	1.33
450–900 nm / 10 nm	46	AisaFENIX to Headwall	PDS	0.649	0.014	-2.52	1.03
450–900 nm / 10 nm	46	AisaFENIX to Headwall	CORAL	0.701	0.011	1.52	0.86
450–950 nm / 5 nm	101	Headwall to AisaFENIX	Direct transfer	0.367	0.009	0.00	0.00
450–950 nm / 5 nm	101	Headwall to AisaFENIX	PDS	0.522	0.012	0.00	0.00
450–950 nm / 5 nm	101	Headwall to AisaFENIX	CORAL	0.674	0.012	0.00	0.00
450–900 nm / 5 nm	91	Headwall to AisaFENIX	Direct transfer	0.401	0.013	3.43	0.96
450–900 nm / 5 nm	91	Headwall to AisaFENIX	PDS	0.529	0.016	0.72	0.96
450–900 nm / 5 nm	91	Headwall to AisaFENIX	CORAL	0.618	0.013	-5.63	0.89
450–950 nm / 10 nm	51	Headwall to AisaFENIX	Direct transfer	0.365	0.010	-0.17	0.31
450–950 nm / 10 nm	51	Headwall to AisaFENIX	PDS	0.464	0.011	-5.77	1.15
450–950 nm / 10 nm	51	Headwall to AisaFENIX	CORAL	0.674	0.012	0.01	0.35
450–900 nm / 10 nm	46	Headwall to AisaFENIX	Direct transfer	0.399	0.013	3.16	0.99
450–900 nm / 10 nm	46	Headwall to AisaFENIX	PDS	0.477	0.014	-4.46	1.36
450–900 nm / 10 nm	46	Headwall to AisaFENIX	CORAL	0.615	0.013	-5.84	0.88

Note: Values are means and descriptive SDs across 30 overlapping pair-preserving splits. The reference grid was 450–950 nm at 5 nm spacing. This sensitivity analysis changes numerical support and sampling only and does not harmonise optical resolution or spectral-response functions.

Table S10. Consolidated analysis parameters and numerical conventions.

Analysis component	Parameter	Implemented value
Preprocessing	Native smoothing polynomial order	3
Preprocessing	AisaFENIX native smoothing window	9 points (h = 4; approximately 15.48 nm)
Preprocessing	Headwall native smoothing window	21 points (h = 10; approximately 15.33 nm)
Preprocessing	Primary common grid	450–950 nm at 5 nm spacing (101 coordinates)
Preprocessing	First-derivative filter	Order 3; 7 points (h = 3); 5 nm increment
PCA–LDA	Retained variance and component limits	99.5%; minimum 2; maximum min(60, n − C, p)
PDS	Local half-window and ridge penalty	h = 2; $\gamma = 10^{-6}$
CORAL	Covariance ridge	$\varepsilon = 10^{-4}$
Repeated robustness analysis	Splits and partition	30 splits; 70 development and 30 test seeds per accession
Fixed-split inference	Development/test size and split seed	2,240/960 seeds; seed 20,960,726
Bootstrap inference	Replicates and seed	4,999; seed 20,960,727
Pairing control	Derangement and seed	Zero fixed points within accession; seed 20,960,728
Multiplicity	Contrasts and correction	12 adaptation-versus-direct-transfer contrasts; Holm adjustment
Exploratory pairing band	Interval and illustrative band	90% interval; ±0.010 macro-F1
Grid sensitivity	PDS half-window	h = 2 on 5 nm grids; h = 1 on 10 nm grids
Compact panels	Regions and source-panel separation	16 equal regions; at least 10 nm separation
Probability analysis	Fitting/calibration/test partition	50/20/30 seeds per accession
Conformal analysis	α, calibration size, and order statistic	0.10; n_cal = 640; k = 577
Calibration error	ECE bins	10 equal-width bins
Importance weighting	PCA components and folds	20 components; 5 folds
Importance weighting	Weight truncation	0.05–20
Software	R version	4.5.2
Software	readxl	1.4.5
Software	data.table	1.17.8
Software	matrixStats	1.5.0
Software	prospectr	0.2.8
Software	vegan	2.7-5
Software	MASS	7.3-65
Software	glmnet	4.1-10
Software	ggplot2	4.0.1
Figures	Python / Matplotlib	3.13 / 3.10.7

The parameters above apply to the primary analyses. Sections S1.7–S1.17 specify the additional analyses, sensitivity settings and comparators. Target-test labels were not used for preprocessing, adaptation, feature selection, or parameter estimation. Target-only and pooled baselines used labels from target calibration seeds, while the CORAL budget subsets were selected to be accession balanced; the additional unbalanced-panel sensitivity is specified in S1.14.

Table S11. Stability of accession geometry within and between instruments and after removal of the leading joint component.

Panel A. Split-half geometry from 50 distinct seeds per accession in each half.

Representation	Comparison	Mantel r, mean ± SD	Procrustes r	RV
Reflectance	Aisa half 1–2	0.985 ± 0.004	0.996	0.991
Reflectance	Headwall half 1–2	0.988 ± 0.003	0.997	0.994
Reflectance	Cross-sensor half 1	0.953 ± 0.006	0.979	0.959
Reflectance	Cross-sensor half 2	0.953 ± 0.006	0.979	0.960
SNV	Aisa half 1–2	0.997 ± 0.001	0.999	0.999
SNV	Headwall half 1–2	0.997 ± 0.001	0.999	0.999
SNV	Cross-sensor half 1	0.995 ± 0.001	0.997	0.996
SNV	Cross-sensor half 2	0.995 ± 0.001	0.997	0.996
First derivative	Aisa half 1–2	0.995 ± 0.001	0.997	0.996
First derivative	Headwall half 1–2	0.995 ± 0.001	0.992	0.996
First derivative	Cross-sensor half 1	0.980 ± 0.002	0.976	0.982
First derivative	Cross-sensor half 2	0.980 ± 0.002	0.977	0.982

Panel B. Cross-sensor geometry before and after removal of PC1 in a common basis.

Representation	Joint PC1 variance	All components, r	PC1 removed, r
Reflectance	70.9%	0.962	0.944
SNV	69.7%	0.996	0.987
First derivative	38.3%	0.985	0.960

Note: Values in Panel A are means and descriptive SDs across 30 overlapping partitions. Cross-sensor halves contain the same physical-seed membership under the fixed-position protocol. The within-sensor split-half comparisons assess accession-centroid stability across different seeds, not repeated measurements of one seed. Panel B uses joint scaling in its own PCA basis; its all-component reference need not equal Table S2C exactly. No significance tests are based on the repeated partitions.

Table S12. Same-seed calibration pairs versus five within-accession derangements per split for PDS.

Representation	Direction	Exact	Deranged	Difference (pp)	SD (pp)	Win rate
Reflectance	Aisa→Headwall	0.674	0.673	+0.12	0.48	0.60
Reflectance	Headwall→Aisa	0.522	0.509	+1.21	0.51	1.00
SNV	Aisa→Headwall	0.707	0.708	-0.12	0.31	0.37

Representation	Direction	Exact	Deranged	Difference (pp)	SD (pp)	Win rate
SNV	Headwall→Aisa	0.834	0.831	+0.21	0.33	0.73
First derivative	Aisa→Headwall	0.580	0.578	+0.23	0.40	0.73
First derivative	Headwall→Aisa	0.813	0.810	+0.26	0.30	0.80

Note: For each representation and direction, 30 overlapping development/test partitions each supplied five fixed-point-free within-accession derangements (150 evaluations). Differences are same-seed minus mean-deranged macro-F1, in percentage points. Descriptive SDs and win rates summarise split-level behaviour and are not inferential estimates from independent biological replicates. The comparison measures the change in PDS performance when calibration uses different seeds from the same accession instead of the same seeds.

Table S13. One-factor sensitivity of PDS, CORAL, and PCA settings across ten specified splits.

Representation	Direction	Method	Setting ID	Macro-F1 mean ± SD	PCs
Reflectance	Aisa→Headwall	CORAL	coral_r1e-2_v995	0.690 ± 0.011	5.0
Reflectance	Aisa→Headwall	CORAL	coral_r1e-4_v990	0.624 ± 0.011	4.0
Reflectance	Aisa→Headwall	CORAL	coral_r1e-4_v995	0.689 ± 0.013	5.0
Reflectance	Aisa→Headwall	CORAL	coral_r1e-4_v999	0.756 ± 0.007	8.1
Reflectance	Aisa→Headwall	CORAL	coral_r1e-6_v995	0.689 ± 0.013	5.0
Reflectance	Headwall→Aisa	CORAL	coral_r1e-2_v995	0.672 ± 0.013	5.0
Reflectance	Headwall→Aisa	CORAL	coral_r1e-4_v990	0.620 ± 0.013	4.0
Reflectance	Headwall→Aisa	CORAL	coral_r1e-4_v995	0.671 ± 0.012	5.0
Reflectance	Headwall→Aisa	CORAL	coral_r1e-4_v999	0.706 ± 0.014	6.0
Reflectance	Headwall→Aisa	CORAL	coral_r1e-6_v995	0.672 ± 0.012	5.0
SNV	Aisa→Headwall	CORAL	coral_r1e-2_v995	0.737 ± 0.013	9.0
SNV	Aisa→Headwall	CORAL	coral_r1e-4_v990	0.719 ± 0.011	14.7
SNV	Aisa→Headwall	CORAL	coral_r1e-4_v995	0.736 ± 0.010	22.5
SNV	Aisa→Headwall	CORAL	coral_r1e-4_v999	0.721 ± 0.016	49.1
SNV	Aisa→Headwall	CORAL	coral_r1e-6_v995	0.738 ± 0.013	26.9
SNV	Headwall→Aisa	CORAL	coral_r1e-2_v995	0.797 ± 0.011	11.0
SNV	Headwall→Aisa	CORAL	coral_r1e-4_v990	0.717 ± 0.016	5.0
SNV	Headwall→Aisa	CORAL	coral_r1e-4_v995	0.766 ± 0.013	7.0
SNV	Headwall→Aisa	CORAL	coral_r1e-4_v999	0.806 ± 0.008	13.0
SNV	Headwall→Aisa	CORAL	coral_r1e-6_v995	0.766 ± 0.013	7.0
First derivative	Aisa→Headwall	CORAL	coral_r1e-2_v995	0.590 ± 0.014	48.0
First derivative	Aisa→Headwall	CORAL	coral_r1e-4_v990	0.629 ± 0.007	55.0
First derivative	Aisa→Headwall	CORAL	coral_r1e-4_v995	0.612 ± 0.015	60.0
First derivative	Aisa→Headwall	CORAL	coral_r1e-4_v999	0.612 ± 0.015	60.0

Representation	Direction	Method	Setting ID	Macro-F1 mean ± SD	PCs
First derivative	Aisa→Headwall	CORAL	coral_r1e-6_v995	0.623 ± 0.009	60.0
First derivative	Headwall→Aisa	CORAL	coral_r1e-2_v995	0.800 ± 0.009	54.0
First derivative	Headwall→Aisa	CORAL	coral_r1e-4_v990	0.800 ± 0.010	23.0
First derivative	Headwall→Aisa	CORAL	coral_r1e-4_v995	0.802 ± 0.010	31.0
First derivative	Headwall→Aisa	CORAL	coral_r1e-4_v999	0.795 ± 0.011	50.0
First derivative	Headwall→Aisa	CORAL	coral_r1e-6_v995	0.802 ± 0.011	31.0
Reflectance	Aisa→Headwall	PDS	pds_h1_r1e-6_v995	0.687 ± 0.007	5.0
Reflectance	Aisa→Headwall	PDS	pds_h2_r1e-4_v995	0.675 ± 0.007	5.0
Reflectance	Aisa→Headwall	PDS	pds_h2_r1e-6_v990	0.617 ± 0.012	4.0
Reflectance	Aisa→Headwall	PDS	pds_h2_r1e-6_v995	0.675 ± 0.007	5.0
Reflectance	Aisa→Headwall	PDS	pds_h2_r1e-6_v999	0.694 ± 0.013	6.0
Reflectance	Aisa→Headwall	PDS	pds_h2_r1e-8_v995	0.675 ± 0.007	5.0
Reflectance	Aisa→Headwall	PDS	pds_h4_r1e-6_v995	0.647 ± 0.014	5.0
Reflectance	Headwall→Aisa	PDS	pds_h1_r1e-6_v995	0.486 ± 0.010	5.0
Reflectance	Headwall→Aisa	PDS	pds_h2_r1e-4_v995	0.516 ± 0.010	5.0
Reflectance	Headwall→Aisa	PDS	pds_h2_r1e-6_v990	0.498 ± 0.013	4.0
Reflectance	Headwall→Aisa	PDS	pds_h2_r1e-6_v995	0.522 ± 0.010	5.0
Reflectance	Headwall→Aisa	PDS	pds_h2_r1e-6_v999	0.561 ± 0.013	15.9
Reflectance	Headwall→Aisa	PDS	pds_h2_r1e-8_v995	0.522 ± 0.010	5.0
Reflectance	Headwall→Aisa	PDS	pds_h4_r1e-6_v995	0.575 ± 0.017	5.0
SNV	Aisa→Headwall	PDS	pds_h1_r1e-6_v995	0.717 ± 0.007	7.0
SNV	Aisa→Headwall	PDS	pds_h2_r1e-4_v995	0.710 ± 0.010	7.0
SNV	Aisa→Headwall	PDS	pds_h2_r1e-6_v990	0.658 ± 0.017	5.0
SNV	Aisa→Headwall	PDS	pds_h2_r1e-6_v995	0.710 ± 0.010	7.0
SNV	Aisa→Headwall	PDS	pds_h2_r1e-6_v999	0.709 ± 0.011	13.0
SNV	Aisa→Headwall	PDS	pds_h2_r1e-8_v995	0.710 ± 0.010	7.0
SNV	Aisa→Headwall	PDS	pds_h4_r1e-6_v995	0.704 ± 0.012	7.0
SNV	Headwall→Aisa	PDS	pds_h1_r1e-6_v995	0.819 ± 0.014	27.1
SNV	Headwall→Aisa	PDS	pds_h2_r1e-4_v995	0.831 ± 0.011	27.1
SNV	Headwall→Aisa	PDS	pds_h2_r1e-6_v990	0.826 ± 0.013	18.0
SNV	Headwall→Aisa	PDS	pds_h2_r1e-6_v995	0.830 ± 0.011	27.1
SNV	Headwall→Aisa	PDS	pds_h2_r1e-6_v999	0.840 ± 0.008	57.9
SNV	Headwall→Aisa	PDS	pds_h2_r1e-8_v995	0.830 ± 0.011	27.1
SNV	Headwall→Aisa	PDS	pds_h4_r1e-6_v995	0.817 ± 0.010	27.1

Representation	Direction	Method	Setting ID	Macro-F1 mean ± SD	PCs
First derivative	Aisa→Headwall	PDS	pds_h1_r1e-6_v995	0.540 ± 0.010	31.0
First derivative	Aisa→Headwall	PDS	pds_h2_r1e-4_v995	0.571 ± 0.014	31.0
First derivative	Aisa→Headwall	PDS	pds_h2_r1e-6_v990	0.573 ± 0.016	23.0
First derivative	Aisa→Headwall	PDS	pds_h2_r1e-6_v995	0.581 ± 0.011	31.0
First derivative	Aisa→Headwall	PDS	pds_h2_r1e-6_v999	0.522 ± 0.011	49.0
First derivative	Aisa→Headwall	PDS	pds_h2_r1e-8_v995	0.581 ± 0.011	31.0
First derivative	Aisa→Headwall	PDS	pds_h4_r1e-6_v995	0.634 ± 0.012	31.0
First derivative	Headwall→Aisa	PDS	pds_h1_r1e-6_v995	0.810 ± 0.011	60.0
First derivative	Headwall→Aisa	PDS	pds_h2_r1e-4_v995	0.787 ± 0.008	60.0
First derivative	Headwall→Aisa	PDS	pds_h2_r1e-6_v990	0.811 ± 0.010	57.0
First derivative	Headwall→Aisa	PDS	pds_h2_r1e-6_v995	0.811 ± 0.011	60.0
First derivative	Headwall→Aisa	PDS	pds_h2_r1e-6_v999	0.811 ± 0.011	60.0
First derivative	Headwall→Aisa	PDS	pds_h2_r1e-8_v995	0.811 ± 0.011	60.0
First derivative	Headwall→Aisa	PDS	pds_h4_r1e-6_v995	0.812 ± 0.011	60.0

Note: The evaluated splits were 1, 4, 7, 11, 14, 17, 21, 24, 27, and 30. One setting was varied at a time around the primary setting: PDS half-window 1/2/4, PDS ridge $10^{-8}/10^{-6}/10^{-4}$, CORAL ridge $10^{-6}/10^{-4}/10^{-2}$, and PCA retained-variance target 0.990/0.995/0.999. Values are mean ± descriptive SD of macro-F1 across overlapping splits. These results do not identify a uniquely optimal setting or support a formal PDS–CORAL ranking.

Table S14. Full-budget transfer comparators including simple reflectance alignment and paired ridge direct standardisation.

Representation	Direction	Direct	PDS	CORAL	Per-band	Gain/offset	Ridge-DS
Reflectance	Aisa→Headwall	0.471	0.674	0.686	0.669	0.579	0.718
Reflectance	Headwall→Aisa	0.367	0.522	0.674	0.659	0.512	0.755
SNV	Aisa→Headwall	0.646	0.707	0.733	—	—	0.743
SNV	Headwall→Aisa	0.629	0.834	0.770	—	—	0.874
First derivative	Aisa→Headwall	0.379	0.580	0.605	—	—	0.752
First derivative	Headwall→Aisa	0.608	0.813	0.805	—	—	0.867

Note: Values are mean macro-F1 scores across 30 overlapping splits with 70 target calibration seeds per accession where required. Per-band matching and global gain/offset were evaluated only for smoothed reflectance; dashes in the SNV and first-derivative cells indicate that those combinations were not evaluated. Paired ridge-DS (primary ridge 10^{-4}) uses 2,240 source–target calibration pairs and is a high-calibration benchmark motivated by Refs. [15,26]. The comparisons apply to the listed architectures and calibration requirements. The DS sensitivity ridges were 10^{-6} and 10^{-2}.

Table S15. Target-calibration budget and labelled baselines for reflectance and SNV.

Representation	Direction	Target seeds/accession	PDS	CORAL	Target-only	Pooled
Reflectance	Aisa→Headwall	1	0.496 ± 0.056	0.659 ± 0.027	not estimable	0.483 ± 0.011
Reflectance	Aisa→Headwall	2	0.554 ± 0.053	0.673 ± 0.018	0.623 ± 0.023	0.495 ± 0.011
Reflectance	Aisa→Headwall	5	0.626 ± 0.028	0.680 ± 0.015	0.696 ± 0.023	0.520 ± 0.011
Reflectance	Aisa→Headwall	10	0.652 ± 0.020	0.684 ± 0.015	0.721 ± 0.014	0.554 ± 0.013
Reflectance	Aisa→Headwall	20	0.666 ± 0.018	0.683 ± 0.015	0.734 ± 0.013	0.598 ± 0.012
Reflectance	Aisa→Headwall	40	0.673 ± 0.014	0.686 ± 0.014	0.743 ± 0.013	0.642 ± 0.011
Reflectance	Aisa→Headwall	70	0.674 ± 0.012	0.686 ± 0.013	0.749 ± 0.011	0.681 ± 0.012
Reflectance	Headwall→Aisa	1	0.407 ± 0.064	0.615 ± 0.037	not estimable	0.385 ± 0.011
Reflectance	Headwall→Aisa	2	0.459 ± 0.047	0.642 ± 0.035	0.578 ± 0.048	0.398 ± 0.010
Reflectance	Headwall→Aisa	5	0.503 ± 0.032	0.657 ± 0.025	0.652 ± 0.037	0.430 ± 0.013
Reflectance	Headwall→Aisa	10	0.506 ± 0.029	0.662 ± 0.027	0.686 ± 0.031	0.472 ± 0.013
Reflectance	Headwall→Aisa	20	0.515 ± 0.016	0.673 ± 0.014	0.709 ± 0.029	0.533 ± 0.016
Reflectance	Headwall→Aisa	40	0.519 ± 0.016	0.674 ± 0.013	0.725 ± 0.014	0.596 ± 0.016
Reflectance	Headwall→Aisa	70	0.522 ± 0.012	0.674 ± 0.012	0.728 ± 0.015	0.639 ± 0.015
SNV	Aisa→Headwall	1	0.616 ± 0.040	0.669 ± 0.020	not estimable	0.606 ± 0.038
SNV	Aisa→Headwall	2	0.663 ± 0.030	0.696 ± 0.016	0.593 ± 0.037	0.603 ± 0.019
SNV	Aisa→Headwall	5	0.689 ± 0.018	0.714 ± 0.015	0.744 ± 0.022	0.688 ± 0.013
SNV	Aisa→Headwall	10	0.698 ± 0.020	0.719 ± 0.015	0.795 ± 0.017	0.733 ± 0.013
SNV	Aisa→Headwall	20	0.701 ± 0.017	0.727 ± 0.013	0.828 ± 0.011	0.777 ± 0.008
SNV	Aisa→Headwall	40	0.704 ± 0.016	0.733 ± 0.014	0.845 ± 0.009	0.812 ± 0.012
SNV	Aisa→Headwall	70	0.707 ± 0.014	0.733 ± 0.012	0.852 ± 0.009	0.831 ± 0.008
SNV	Headwall→Aisa	1	0.721 ± 0.062	0.717 ± 0.026	not estimable	0.667 ± 0.015
SNV	Headwall→Aisa	2	0.772 ± 0.034	0.744 ± 0.019	0.691 ± 0.037	0.696 ± 0.016
SNV	Headwall→Aisa	5	0.810 ± 0.017	0.760 ± 0.015	0.774 ± 0.018	0.755 ± 0.018
SNV	Headwall→Aisa	10	0.824 ± 0.012	0.763 ± 0.015	0.802 ± 0.015	0.807 ± 0.016
SNV	Headwall→Aisa	20	0.831 ± 0.011	0.766 ± 0.013	0.813 ± 0.011	0.841 ± 0.011
SNV	Headwall→Aisa	40	0.833 ± 0.009	0.768 ± 0.013	0.819 ± 0.011	0.858 ± 0.011
SNV	Headwall→Aisa	70	0.834 ± 0.011	0.770 ± 0.013	0.822 ± 0.013	0.871 ± 0.011

Note: Each value is the mean ± descriptive SD of macro-F1 over 30 overlapping splits. Target-only and pooled PCA–LDA use target calibration labels; CORAL uses accession-balanced target calibration subsets but no labels in the alignment objective; PDS uses matched source–target calibration pairs. No target-test labels were used for fitting. Target-only PCA–LDA with one labelled seed per accession is not estimable because 32 observations for 32 classes leave zero residual degrees of freedom; this cell is not zero.

Table S16. Accession geometry and transfer sensitivity restricted to 30 Phaseolus accessions.

Panel A. Cross-sensor accession geometry after exclusion of A27 and A28.

Representation	Mantel r	Procrustes r	RV
Reflectance	0.956	0.980	0.963
SNV	0.997	0.998	0.997
First derivative	0.984	0.980	0.985

Panel B. Same-sensor reference and cross-sensor macro-F1 after exclusion.

Representation	Direction	Scenario	Macro-F1 mean ± SD
Reflectance	Aisa→Headwall	Within sensor	0.733 ± 0.015
Reflectance	Aisa→Headwall	Direct	0.483 ± 0.011
Reflectance	Aisa→Headwall	PDS	0.668 ± 0.011
Reflectance	Aisa→Headwall	CORAL	0.691 ± 0.014
Reflectance	Headwall→Aisa	Within sensor	0.751 ± 0.012
Reflectance	Headwall→Aisa	Direct	0.393 ± 0.011
Reflectance	Headwall→Aisa	PDS	0.482 ± 0.011
Reflectance	Headwall→Aisa	CORAL	0.674 ± 0.012
SNV	Aisa→Headwall	Within sensor	0.783 ± 0.012
SNV	Aisa→Headwall	Direct	0.602 ± 0.010
SNV	Aisa→Headwall	PDS	0.666 ± 0.014
SNV	Aisa→Headwall	CORAL	0.731 ± 0.016
SNV	Headwall→Aisa	Within sensor	0.844 ± 0.009
SNV	Headwall→Aisa	Direct	0.657 ± 0.020
SNV	Headwall→Aisa	PDS	0.825 ± 0.011
SNV	Headwall→Aisa	CORAL	0.745 ± 0.014
First derivative	Aisa→Headwall	Within sensor	0.919 ± 0.009
First derivative	Aisa→Headwall	Direct	0.378 ± 0.016
First derivative	Aisa→Headwall	PDS	0.573 ± 0.015
First derivative	Aisa→Headwall	CORAL	0.609 ± 0.014
First derivative	Headwall→Aisa	Within sensor	0.854 ± 0.007
First derivative	Headwall→Aisa	Direct	0.654 ± 0.016
First derivative	Headwall→Aisa	PDS	0.824 ± 0.013
First derivative	Headwall→Aisa	CORAL	0.815 ± 0.014

Note: A27 (Vigna) and A28 (Cajanus) were excluded; the remaining 30 Phaseolus accessions constitute the taxonomic sensitivity subset. Panel B values are means ± descriptive SD across 30 overlapping splits. Both adaptation scenarios exceeded direct transfer in every representation and direction, although individual mean macro-F1 values changed by up to approximately 0.046.

Table S17. Accession–lot geometry and sensitivity analyses.

A. Observed full-domain geometry and seed-bootstrap intervals.

Representation	Distance correlation ρ [95% CI]	Procrustes r [95% CI]	RV coefficient [95% CI]
Reflectance	0.958 [0.943, 0.964]	0.981 [0.974, 0.983]	0.963 [0.949, 0.969]
SNV	0.996 [0.994, 0.996]	0.997 [0.996, 0.997]	0.997 [0.995, 0.997]
First derivative	0.982 [0.977, 0.983]	0.979 [0.973, 0.980]	0.984 [0.978, 0.985]

B. Wavelength-region and taxonomic-subset sensitivity.

Representation	Accession set	Interval (nm)	Coordinates	Distance ρ	Procrustes r	RV
Reflectance	All 32	450–950	101	0.958	0.981	0.963
Reflectance	All 32	450–700	51	0.978	0.993	0.986
Reflectance	All 32	700–950	51	0.906	0.968	0.942
Reflectance	Phaseolus 30	450–950	101	0.956	0.980	0.963
Reflectance	Phaseolus 30	450–700	51	0.979	0.993	0.987
Reflectance	Phaseolus 30	700–950	51	0.878	0.967	0.941
SNV	All 32	450–950	101	0.996	0.997	0.997
SNV	All 32	450–700	51	0.995	0.997	0.996
SNV	All 32	700–950	51	0.993	0.996	0.994
SNV	Phaseolus 30	450–950	101	0.997	0.998	0.997
SNV	Phaseolus 30	450–700	51	0.996	0.998	0.997
SNV	Phaseolus 30	700–950	51	0.994	0.996	0.994
First derivative	All 32	450–950	101	0.982	0.979	0.984
First derivative	All 32	450–700	51	0.973	0.983	0.979
First derivative	All 32	700–950	51	0.961	0.964	0.971
First derivative	Phaseolus 30	450–950	101	0.984	0.980	0.985
First derivative	Phaseolus 30	450–700	51	0.977	0.984	0.981
First derivative	Phaseolus 30	700–950	51	0.962	0.967	0.971

Note: Aisa denotes AisaFENIX throughout Tables S17–S25. Panel A gives observed coefficients with 95% percentile intervals from 999 seed-bootstrap samples stratified by accession; each sampled index was applied to both sensors. Intervals are conditional on the same accession–lots and acquisition setting. Panel B recomputes wavelength-wise standardisation after selecting the region and accession set. Phaseolus 30 excludes A27 and A28. The two narrower regions share the 700 nm boundary and are not independent datasets. Distance ρ is the Spearman correlation of corresponding inter-accession distances; these rows do not add new permutation tests.

Table S18. Cross-accession derangement control for PDS.

Representation	Direction	Exact PDS mean ± SD	Cross-accession PDS mean ± SD	Exact minus control mean ± SD (pp)
Reflectance	Aisa → Headwall	0.674 ± 0.012	0.008 ± 0.004	66.65 ± 1.20
Reflectance	Headwall → Aisa	0.522 ± 0.012	0.008 ± 0.003	51.39 ± 1.36
SNV	Aisa → Headwall	0.707 ± 0.014	0.010 ± 0.005	69.75 ± 1.46
SNV	Headwall → Aisa	0.834 ± 0.011	0.010 ± 0.003	82.38 ± 1.04
First derivative	Aisa → Headwall	0.580 ± 0.012	0.004 ± 0.002	57.62 ± 1.19

Representation	Direction	Exact PDS mean ± SD	Cross-accession PDS mean ± SD	Exact minus control mean ± SD (pp)
First derivative	Headwall → Aisa	0.813 ± 0.011	0.006 ± 0.003	80.68 ± 1.10

Note: Each of 30 pair-preserving 70:30 splits used five independently generated cross-accession derangements. All calibration spectra were used once per draw, and every source seed was matched to a target seed from a different accession. Control results were first averaged over the five draws within each split; SDs then describe the 30 overlapping split-level means. Same-seed PDS uses the identical development and test partitions. The control tests the effect of destroying accession correspondence; the incremental effect of same-seed calibration within an accession is examined separately in Tables S4 and S12. SDs are descriptive; pp denotes macro-F1 percentage points.

Table S19. Within-sensor shrinkage-LDA sensitivity.

Representation	Sensor	Shrinkage λ	Macro-F1 mean ± SD	Central 95% range
Reflectance	AisaFENIX	0.01	0.897 ± 0.010	[0.873, 0.923]
Reflectance	AisaFENIX	0.1	0.843 ± 0.014	[0.801, 0.871]
Reflectance	AisaFENIX	0.5	0.759 ± 0.015	[0.725, 0.779]
Reflectance	Headwall	0.01	0.871 ± 0.009	[0.856, 0.884]
Reflectance	Headwall	0.1	0.838 ± 0.010	[0.821, 0.859]
Reflectance	Headwall	0.5	0.772 ± 0.013	[0.746, 0.805]
SNV	AisaFENIX	0.01	0.943 ± 0.006	[0.933, 0.956]
SNV	AisaFENIX	0.1	0.929 ± 0.007	[0.919, 0.946]
SNV	AisaFENIX	0.5	0.887 ± 0.010	[0.870, 0.907]
SNV	Headwall	0.01	0.865 ± 0.009	[0.848, 0.883]
SNV	Headwall	0.1	0.867 ± 0.009	[0.849, 0.883]
SNV	Headwall	0.5	0.837 ± 0.007	[0.822, 0.850]
First derivative	AisaFENIX	0.01	0.932 ± 0.007	[0.918, 0.950]
First derivative	AisaFENIX	0.1	0.926 ± 0.007	[0.910, 0.938]
First derivative	AisaFENIX	0.5	0.909 ± 0.009	[0.892, 0.927]
First derivative	Headwall	0.01	0.849 ± 0.009	[0.833, 0.867]
First derivative	Headwall	0.1	0.851 ± 0.009	[0.834, 0.870]
First derivative	Headwall	0.5	0.835 ± 0.009	[0.821, 0.855]

Note: The analysis used the same 30 pair-preserving 70:30 splits. Feature-scaling parameters, class means and the pooled within-class covariance were estimated using development seeds only. Covariance shrinkage targets a scaled identity matrix, with λ = 0.01, 0.1 or 0.5; priors are equal across the 32 accessions. These fixed settings form a sensitivity grid, not a test-set-selected optimum. Ranges are empirical 2.5th–97.5th percentiles across overlapping splits and are descriptive, not confidence intervals. Shrinkage LDA provides a within-sensor reference for the PCA–LDA transfer comparisons.

Table S20. Descriptive distributions of repeated-split transfer gains.

Representation	Direction	Method	Gain over direct mean ± SD (pp)	Central 95% range (pp)
Reflectance	Aisa → Headwall	PDS	20.32 ± 1.26	[17.96, 22.51]
Reflectance	Aisa → Headwall	CORAL	21.51 ± 1.47	[18.85, 24.56]
Reflectance	Headwall → Aisa	PDS	15.43 ± 1.19	[13.32, 17.85]
Reflectance	Headwall → Aisa	CORAL	30.66 ± 1.32	[27.73, 33.72]
SNV	Aisa → Headwall	PDS	6.08 ± 1.52	[2.30, 8.61]
SNV	Aisa → Headwall	CORAL	8.66 ± 1.51	[4.84, 11.37]
SNV	Headwall → Aisa	PDS	20.40 ± 1.46	[18.17, 24.12]

Representation	Direction	Method	Gain over direct mean ± SD (pp)	Central 95% range (pp)
SNV	Headwall → Aisa	CORAL	14.01 ± 1.58	[11.47, 17.43]
First derivative	Aisa → Headwall	PDS	20.11 ± 1.33	[18.06, 23.20]
First derivative	Aisa → Headwall	CORAL	22.54 ± 1.96	[19.05, 27.42]
First derivative	Headwall → Aisa	PDS	20.43 ± 2.07	[16.70, 24.02]
First derivative	Headwall → Aisa	CORAL	19.63 ± 2.05	[15.97, 24.46]

Note: For each of the 30 overlapping pair-preserving splits, macro-F1 for direct transfer was subtracted from the adapted result on the identical held-out seeds. Ranges are the empirical 2.5th–97.5th percentiles of these paired differences and are descriptive, not confidence intervals. The overlapping splits are not independent replications. Fixed-split paired-bootstrap inference is reported separately in Table 3 and Table S8.

Table S21. Low-budget CORAL composition, covariance and parameter sensitivity.

A. Balanced and random target-calibration subsets.

Direction	Subset design	Target spectra	Macro-F1 mean ± SD	Accessions mean [range]	Rank range	Mean regularised condition number
Aisa → Headwall	Balanced	32	0.655 ± 0.019	32.00 [32]	31	1.05×10^6
Aisa → Headwall	Random	32	0.510 ± 0.036	20.44 [17–24]	31	1.04×10^6
Aisa → Headwall	Balanced	64	0.671 ± 0.013	32.00 [32]	63	1.01×10^6
Aisa → Headwall	Random	64	0.592 ± 0.019	27.98 [25–30]	63	1.01×10^6
Aisa → Headwall	Balanced	160	0.682 ± 0.014	32.00 [32]	101	8.77×10^5
Aisa → Headwall	Random	160	0.647 ± 0.015	31.86 [31–32]	101	8.73×10^5
Headwall → Aisa	Balanced	32	0.618 ± 0.018	32.00 [32]	31	5.24×10^5
Headwall → Aisa	Random	32	0.496 ± 0.036	20.44 [17–24]	31	5.35×10^5
Headwall → Aisa	Balanced	64	0.639 ± 0.017	32.00 [32]	63	5.15×10^5
Headwall → Aisa	Random	64	0.562 ± 0.027	27.98 [25–30]	63	5.16×10^5
Headwall → Aisa	Balanced	160	0.652 ± 0.017	32.00 [32]	101	5.12×10^5
Headwall → Aisa	Random	160	0.615 ± 0.019	31.86 [31–32]	101	5.04×10^5

B. One-factor sensitivity with one target spectrum per accession.

Direction	CORAL ε	PCA target (%)	Macro-F1 mean ± SD	Retained PCs range	Mean regularised condition number
Aisa → Headwall	1e-04	99.5	0.658 ± 0.022	5	9.98×10^5
Aisa → Headwall	1e-06	99.5	0.658 ± 0.022	5	9.98×10^7
Aisa → Headwall	1e-02	99.5	0.661 ± 0.022	5	9.98×10^3
Aisa → Headwall	1e-04	99.0	0.594 ± 0.035	4	9.98×10^5
Aisa → Headwall	1e-04	99.9	0.716 ± 0.027	7–9	9.98×10^5
Headwall → Aisa	1e-04	99.5	0.622 ± 0.040	4–5	5.53×10^5

Direction	CORAL ε	PCA target (%)	Macro-F1 mean ± SD	Retained PCs range	Mean regularised condition number
Headwall → Aisa	1e-06	99.5	0.622 ± 0.040	4–5	5.53×10^7
Headwall → Aisa	1e-02	99.5	0.641 ± 0.021	4–5	5.53×10^3
Headwall → Aisa	1e-04	99.0	0.596 ± 0.021	4	5.53×10^5
Headwall → Aisa	1e-04	99.9	0.692 ± 0.021	6–7	5.53×10^5

Note: Smoothed-reflectance analyses use ten prescribed outer splits. Panel A uses five draws per split; macro-F1 is first averaged over draws and then summarised as mean ± SD across the ten split means. Accession counts, covariance ranks and condition numbers summarise all 50 draws. Balanced subsets contain 1, 2 or 5 spectra from every accession; random subsets contain the same total without accession balancing. Ranks refer to the target covariance after pooled scaling and before regularisation, and condition numbers refer to that covariance after adding εI, with $\varepsilon = 10^{-4}$. Rank-deficient unregularised covariances are singular. Panel B uses the first balanced 32-spectrum draw per split and varies one setting at a time around $\varepsilon = 10^{-4}$ and a 99.5% retained-variance target. These settings are sensitivities, not parameters selected on the target test set. All SDs are descriptive. The balanced 32-spectrum result in Table S4 uses one subset in each of 30 outer splits, whereas Panel A here averages five draws in each of ten outer splits; their means therefore summarise different resampling designs.

Table S22. Held-out linear domain discrimination.

Direction	Scenario	Held-out AUC mean ± SD	Central 95% range	Development CV AUC	Splits with constant scores
Aisa → Headwall	Direct	0.822 ± 0.006	[0.809, 0.838]	0.822	0%
Aisa → Headwall	PDS	0.500 ± 0.000	[0.500, 0.500]	0.488	100%
Aisa → Headwall	CORAL	0.500 ± 0.000	[0.500, 0.500]	0.489	100%
Headwall → Aisa	Direct	0.822 ± 0.006	[0.809, 0.838]	0.822	0%
Headwall → Aisa	PDS	0.500 ± 0.000	[0.500, 0.500]	0.487	100%
Headwall → Aisa	CORAL	0.500 ± 0.000	[0.500, 0.500]	0.489	100%

Note: For each of 30 pair-preserving 70:30 splits, adaptation and domain-classifier preprocessing were fitted on development spectra, and domain AUC was then evaluated on 960 held-out physical pairs (1,920 spectra). The classifier uses a development-fitted standardised PCA space and regularised logistic regression. Ranges across overlapping splits are descriptive. Every post-PDS and post-CORAL fit returned a constant probability, producing AUC = 0.500 by tied ranks; this is a limitation of the fitted linear diagnostic and does not establish domain equivalence. The unadapted domain problem is the same when the source/target labels are reversed, explaining the identical direct AUC summaries.

Table S23. Sensitivity to native-axis smoothing span.

A. Native-axis Savitzky–Golay windows.

Sensor	Requested span (nm)	Window points	Median native step (nm)	Points × step (nm)	Approx. first-to-last span (nm)
AisaFENIX	5	5	1.72	8.60	6.88
Headwall	5	7	0.73	5.11	4.38
AisaFENIX	15	9	1.72	15.48	13.76
Headwall	15	21	0.73	15.33	14.60
AisaFENIX	25	15	1.72	25.80	24.08
Headwall	25	35	0.73	25.55	24.82

B. Macro-F1 after changing the requested native-axis smoothing span.

Representation	Direction	Scenario	5 nm mean ± SD	15 nm mean ± SD	25 nm mean ± SD
Reflectance	Aisa → Headwall	Direct	0.472 ± 0.012	0.470 ± 0.013	0.470 ± 0.013
Reflectance	Aisa → Headwall	PDS	0.681 ± 0.010	0.675 ± 0.007	0.669 ± 0.010

Representation	Direction	Scenario	5 nm mean ± SD	15 nm mean ± SD	25 nm mean ± SD
Reflectance	Aisa → Headwall	CORAL	0.686 ± 0.015	0.689 ± 0.013	0.692 ± 0.013
Reflectance	Headwall → Aisa	Direct	0.418 ± 0.011	0.368 ± 0.009	0.368 ± 0.008
Reflectance	Headwall → Aisa	PDS	0.493 ± 0.014	0.522 ± 0.010	0.533 ± 0.012
Reflectance	Headwall → Aisa	CORAL	0.673 ± 0.012	0.671 ± 0.012	0.672 ± 0.012
SNV	Aisa → Headwall	Direct	0.575 ± 0.015	0.648 ± 0.014	0.658 ± 0.013
SNV	Aisa → Headwall	PDS	0.681 ± 0.010	0.710 ± 0.010	0.714 ± 0.010
SNV	Aisa → Headwall	CORAL	0.725 ± 0.013	0.736 ± 0.010	0.719 ± 0.014
SNV	Headwall → Aisa	Direct	0.665 ± 0.014	0.627 ± 0.014	0.657 ± 0.017
SNV	Headwall → Aisa	PDS	0.829 ± 0.010	0.830 ± 0.011	0.824 ± 0.011
SNV	Headwall → Aisa	CORAL	0.761 ± 0.012	0.766 ± 0.013	0.772 ± 0.013
First derivative	Aisa → Headwall	Direct	0.334 ± 0.014	0.379 ± 0.014	0.412 ± 0.009
First derivative	Aisa → Headwall	PDS	0.500 ± 0.007	0.581 ± 0.011	0.584 ± 0.011
First derivative	Aisa → Headwall	CORAL	0.611 ± 0.017	0.612 ± 0.015	0.629 ± 0.015
First derivative	Headwall → Aisa	Direct	0.596 ± 0.015	0.608 ± 0.019	0.610 ± 0.017
First derivative	Headwall → Aisa	PDS	0.801 ± 0.009	0.811 ± 0.011	0.811 ± 0.011
First derivative	Headwall → Aisa	CORAL	0.798 ± 0.009	0.802 ± 0.010	0.803 ± 0.010

Note: The same ten prescribed pair-preserving 70:30 splits were evaluated at all three spans. Cubic Savitzky–Golay smoothing used an odd window of at least five points, so requested and achieved spans differ, particularly for AisaFENIX at 5 nm. Spectra were then resampled to the reference 450–950 nm, 5 nm grid; SNV and the seven-point first derivative were recomputed. The 15 nm settings exactly reproduce the corresponding reference feature matrices and prediction metrics. SDs describe variation across overlapping splits. This numerical preprocessing sensitivity does not estimate or equalise optical FWHM.

Table S24. Minimum-redundancy maximum-relevance wavelength-panel comparator.

Direction	Bands	Splits	Direct mean ± SD [range]	PDS mean ± SD [range]	CORAL mean ± SD [range]
Aisa → Headwall	8	10	0.390 ± 0.014 [0.368, 0.409]	0.627 ± 0.034 [0.541, 0.676]	0.650 ± 0.012 [0.633, 0.673]
Aisa → Headwall	16	30	0.456 ± 0.035 [0.376, 0.495]	0.631 ± 0.025 [0.565, 0.657]	0.676 ± 0.015 [0.651, 0.699]
Aisa → Headwall	32	10	0.473 ± 0.012 [0.455, 0.496]	0.657 ± 0.013 [0.639, 0.677]	0.682 ± 0.011 [0.668, 0.699]
Headwall → Aisa	8	10	0.321 ± 0.025 [0.294, 0.374]	0.581 ± 0.017 [0.562, 0.610]	0.602 ± 0.020 [0.577, 0.637]
Headwall → Aisa	16	30	0.376 ± 0.033 [0.289, 0.416]	0.588 ± 0.039 [0.499, 0.643]	0.647 ± 0.028 [0.589, 0.678]
Headwall → Aisa	32	10	0.382 ± 0.012 [0.358, 0.403]	0.579 ± 0.016 [0.553, 0.606]	0.663 ± 0.014 [0.633, 0.686]

Note: mRMR uses the mutual-information-difference criterion with empirical decile discretisation fitted to pooled source- and target-development spectra and categorical accession labels. Both directions share the same development-only ranking. Target test spectra and labels were excluded from ranking, binning and fitting. The 16-band comparator uses the same 30 compact-panel splits; the 8- and 32-band sensitivities use the same ten prescribed split IDs. Brackets give central 95% empirical ranges across overlapping splits, not confidence intervals. PDS neighbourhoods follow adjacent selected columns. No band count or selection parameter was chosen on the basis of target test performance.

Table S25. Variability of calibration, prediction-set coverage and selective accuracy.

A. Classification and calibration.

Direction	Scenario	ECE mean ± SD [range]	Accuracy mean ± SD [range]	Macro-F1 mean ± SD [range]
Aisa → Headwall	Direct	0.296 ± 0.016 [0.271, 0.328]	0.511 ± 0.014 [0.487, 0.539]	0.465 ± 0.017 [0.430, 0.491]
Aisa → Headwall	PDS	0.041 ± 0.011 [0.025, 0.072]	0.679 ± 0.013 [0.657, 0.703]	0.676 ± 0.014 [0.655, 0.703]
Aisa → Headwall	CORAL	0.054 ± 0.009 [0.037, 0.072]	0.693 ± 0.009 [0.678, 0.711]	0.684 ± 0.011 [0.665, 0.704]
Headwall → Aisa	Direct	0.365 ± 0.015 [0.344, 0.395]	0.400 ± 0.013 [0.377, 0.419]	0.362 ± 0.014 [0.337, 0.384]
Headwall → Aisa	PDS	0.166 ± 0.015 [0.139, 0.199]	0.534 ± 0.016 [0.494, 0.565]	0.518 ± 0.018 [0.474, 0.554]
Headwall → Aisa	CORAL	0.082 ± 0.010 [0.059, 0.101]	0.673 ± 0.010 [0.650, 0.693]	0.668 ± 0.010 [0.647, 0.690]

B. Prediction-set coverage and selective accuracy.

Direction	Scenario	Standard coverage mean ± SD [range]	Weighted coverage mean ± SD [range]	Accuracy at 50% retention mean ± SD [range]
Aisa → Headwall	Direct	0.679 ± 0.023 [0.628, 0.723]	0.665 ± 0.022 [0.628, 0.709]	0.668 ± 0.020 [0.637, 0.704]
Aisa → Headwall	PDS	0.910 ± 0.015 [0.879, 0.936]	0.910 ± 0.015 [0.879, 0.936]	0.855 ± 0.015 [0.832, 0.887]
Aisa → Headwall	CORAL	0.900 ± 0.017 [0.870, 0.929]	0.900 ± 0.017 [0.870, 0.929]	0.865 ± 0.009 [0.842, 0.883]
Headwall → Aisa	Direct	0.559 ± 0.030 [0.491, 0.636]	0.596 ± 0.068 [0.491, 0.754]	0.503 ± 0.022 [0.469, 0.549]
Headwall → Aisa	PDS	0.751 ± 0.026 [0.700, 0.813]	0.751 ± 0.026 [0.700, 0.813]	0.656 ± 0.019 [0.617, 0.691]
Headwall → Aisa	CORAL	0.844 ± 0.019 [0.809, 0.877]	0.844 ± 0.019 [0.809, 0.877]	0.826 ± 0.013 [0.805, 0.858]

C. Paired weighted-minus-standard coverage difference.

Direction	Scenario	Coverage difference mean ± SD (pp)	Central 95% range (pp)
Aisa → Headwall	Direct	−1.43 ± 1.54	[−5.62, 0.63]
Aisa → Headwall	PDS	0.00 ± 0.00	[0.00, 0.00]
Aisa → Headwall	CORAL	0.00 ± 0.00	[0.00, 0.00]
Headwall → Aisa	Direct	3.74 ± 5.43	[−5.21, 14.72]
Headwall → Aisa	PDS	0.00 ± 0.00	[0.00, 0.00]
Headwall → Aisa	CORAL	0.00 ± 0.00	[0.00, 0.00]

Note: These smoothed-reflectance analyses use the separate 50/20/30 seed-per-accession design: source fitting, source conformal calibration and held-out testing, respectively. Both measurements of calibration and test seeds were excluded from adaptation. All entries summarise 30 overlapping pair-preserving splits; bracketed 2.5th–97.5th percentile ranges and SDs are descriptive, not confidence intervals. Prediction-set coverage has a nominal target of 0.90 but no target-domain guarantee. Selective accuracy is evaluated among the 50% most confident target seeds. Panel C pairs the two coverage procedures within each split. Zero differences after PDS/CORAL reflect the fitted nearly uniform importance weights.

Table S26. Within-accession cross-sensor associations in mean reflectance and PC1, with order-sensitivity summaries.

Panel A. Same-seed correlations summarised across the 32 accessions.

Summary	Median r	r (Q1–Q3)	Permutation p	Median ρ	Permutation p (ρ)
Mean reflectance	-0.016	-0.080 to 0.056	0.4700	-0.011	0.6302
PC1	-0.008	-0.074 to 0.059	0.7084	-0.009	0.6933

Panel B. Same-seed correlations within each accession.

Accession	Mean reflectance: r	Mean reflectance: p	Mean reflectance: Holm-adjusted p	PC1 r	PC1 p	PC1: Holm-adjusted p
A01	-0.061	0.5460	1.000	-0.039	0.6994	1.000
A02	0.159	0.1170	1.000	0.153	0.1314	1.000
A03	-0.016	0.8770	1.000	-0.019	0.8510	1.000
A04	0.007	0.9465	1.000	-0.000	0.9982	1.000
A05	0.126	0.2108	1.000	0.123	0.2240	1.000
A06	-0.082	0.4188	1.000	-0.081	0.4237	1.000
A07	-0.047	0.6459	1.000	-0.065	0.5176	1.000
A08	-0.113	0.2660	1.000	-0.114	0.2647	1.000
A09	0.046	0.6511	1.000	0.055	0.5878	1.000
A10	-0.131	0.2022	1.000	-0.138	0.1754	1.000
A11	0.001	0.9893	1.000	-0.006	0.9557	1.000
A12	-0.005	0.9564	1.000	0.007	0.9422	1.000
A13	-0.019	0.8530	1.000	-0.023	0.8247	1.000
A14	0.081	0.4287	1.000	0.070	0.4919	1.000
A15	-0.233	0.0195	1.000	-0.227	0.0226	1.000
A16	-0.016	0.8710	1.000	-0.006	0.9521	1.000
A17	-0.029	0.7740	1.000	-0.034	0.7364	1.000
A18	-0.172	0.0852	1.000	-0.168	0.0941	1.000
A19	0.033	0.7528	1.000	0.031	0.7629	1.000
A20	0.033	0.7431	1.000	0.025	0.8067	1.000
A21	-0.138	0.1708	1.000	-0.115	0.2514	1.000
A22	-0.018	0.8583	1.000	-0.011	0.9078	1.000
A23	0.157	0.1260	1.000	0.161	0.1168	1.000
A24	0.121	0.2246	1.000	0.129	0.1986	1.000
A25	-0.153	0.1337	1.000	-0.141	0.1659	1.000
A26	0.194	0.0512	1.000	0.194	0.0488	1.000
A27	-0.078	0.4518	1.000	-0.054	0.5976	1.000
A28	0.057	0.5743	1.000	0.036	0.7294	1.000
A29	0.054	0.6004	1.000	0.062	0.5468	1.000
A30	-0.036	0.7249	1.000	-0.033	0.7503	1.000
A31	-0.095	0.3489	1.000	-0.094	0.3547	1.000
A32	0.082	0.4098	1.000	0.093	0.3557	1.000

Panel C. Accession-median correlations under index shifts and reversal.

Offset / order	n per accession	Mean reflectance: median r	Mean reflectance: adjusted p	PC1 median r	PC1 adjusted p
-10	90	0.040	0.8667	0.031	0.9922
-9	91	0.021	1.0000	0.014	1.0000
-8	92	0.011	1.0000	0.012	1.0000
-7	93	-0.012	1.0000	-0.016	1.0000
-6	94	-0.023	0.9998	-0.037	0.9363
-5	95	-0.010	1.0000	-0.009	1.0000
-4	96	-0.022	1.0000	-0.022	1.0000
-3	97	0.048	0.6073	0.049	0.5813
-2	98	0.011	1.0000	0.009	1.0000
-1	99	-0.020	1.0000	-0.024	0.9998
+0	100	-0.016	1.0000	-0.008	1.0000
+1	99	-0.016	1.0000	-0.008	1.0000
+2	98	-0.006	1.0000	-0.001	1.0000
+3	97	0.010	1.0000	0.009	1.0000
+4	96	0.011	1.0000	0.020	1.0000
+5	95	-0.038	0.9167	-0.039	0.9039
+6	94	-0.008	1.0000	-0.009	1.0000
+7	93	0.021	1.0000	0.014	1.0000
+8	92	0.029	0.9958	0.030	0.9947
+9	91	0.070	0.0562	0.067	0.0838
+10	90	0.022	1.0000	0.021	1.0000
Reversed	100	-0.014	1.0000	0.001	1.0000

Note: Mean reflectance and sensor-specific covariance PC1 were computed from smoothed reflectance at the 101 common wavelengths, without SNV or row standardisation. Panel A summarises Pearson r across the 32 accessions and includes the median Spearman correlation as a sensitivity analysis. Panel B reports Pearson r for the 100 same-seed pairs within each accession; Holm adjustment covers the 64 metric-by-accession comparisons. In Panel C, offset k pairs AisaFENIX index i with Headwall index i + k using 100 − |k| overlapping observations; reversal pairs i with 101 − i. These perturbed orders are sensitivity controls. Two-sided permutation p values use 9,999 within-accession permutations; max-statistic p values adjust jointly for the 44 metric-by-order combinations of the accession-median statistic. The minimum attainable unadjusted p value is 0.0001.

PC1 explained 78.480% and 78.546% of the variance in AisaFENIX and Headwall reflectance, respectively, and correlated with each sensor's mean reflectance at r = 0.989 and 0.990. Across accessions, median same-seed Pearson correlations were −0.016 for mean reflectance and −0.008 for PC1 (two-sided permutation p = 0.4700 and 0.7084). Corresponding median Spearman correlations were −0.011 and −0.009 (p = 0.6302 and 0.6933). All 64 zero-offset Holm-adjusted p values were 1.0000. The largest positive accession-median correlations occurred at offset +9: 0.070 and 0.067, with max-statistic-adjusted p = 0.0562 and 0.0838 for mean reflectance and PC1, respectively. Reversal yielded median correlations of −0.014 and 0.001, both with adjusted p = 1.0000. Across all 1,408 accession-by-metric-by-order comparisons, the smallest Holm-adjusted p value was 0.2816. These results show weak within-accession cross-sensor association for these two magnitude summaries; the PC1 results largely reflect the same brightness variation as mean reflectance.
